# The Genome of a Pathogenic *Rhodococcus*: Cooptive Virulence Underpinned by Key Gene Acquisitions

**DOI:** 10.1371/journal.pgen.1001145

**Published:** 2010-09-30

**Authors:** Michal Letek, Patricia González, Iain MacArthur, Héctor Rodríguez, Tom C. Freeman, Ana Valero-Rello, Mónica Blanco, Tom Buckley, Inna Cherevach, Ruth Fahey, Alexia Hapeshi, Jolyon Holdstock, Desmond Leadon, Jesús Navas, Alain Ocampo, Michael A. Quail, Mandy Sanders, Mariela M. Scortti, John F. Prescott, Ursula Fogarty, Wim G. Meijer, Julian Parkhill, Stephen D. Bentley, José A. Vázquez-Boland

**Affiliations:** 1 Microbial Pathogenesis Unit, Centres for Infectious Diseases and Immunity, Infection, and Evolution, University of Edinburgh, Edinburgh, United Kingdom; 2 Irish Equine Centre, Johnstown, Naas, Ireland; 3 Department of Pathobiology, University of Guelph, Guelph, Canada; 4 Division of Genetics and Genomics, Roslin BioCentre, University of Edinburgh, Edinburgh, United Kingdom; 5 Pathogen Genomics, Wellcome Trust Sanger Institute, Cambridge, United Kingdom; 6 School of Biomolecular and Biomedical Sciences, University College Dublin, Dublin, Ireland; 7 Oxford Gene Technology, Begbroke Science Park, Oxford, United Kingdom; 8 Departamento de Biología Molecular, Universidad de Cantabria, Santander, Spain; 9 Departamento de Bioquímica y Biología Molecular IV, Universidad Complutense, Madrid, Spain; 10 Grupo de Patogenómica Bacteriana, Universidad de León, León, Spain; Universidad de Sevilla, Spain

## Abstract

We report the genome of the facultative intracellular parasite *Rhodococcus equi*, the only animal pathogen within the biotechnologically important actinobacterial genus *Rhodococcu*s. The 5.0-Mb *R. equi* 103S genome is significantly smaller than those of environmental rhodococci. This is due to genome expansion in nonpathogenic species, via a linear gain of paralogous genes and an accelerated genetic flux, rather than reductive evolution in *R. equi*. The 103S genome lacks the extensive catabolic and secondary metabolic complement of environmental rhodococci, and it displays unique adaptations for host colonization and competition in the short-chain fatty acid–rich intestine and manure of herbivores—two main *R. equi* reservoirs. Except for a few horizontally acquired (HGT) pathogenicity loci, including a cytoadhesive pilus determinant (*rpl*) and the virulence plasmid *vap* pathogenicity island (PAI) required for intramacrophage survival, most of the potential virulence-associated genes identified in *R. equi* are conserved in environmental rhodococci or have homologs in nonpathogenic *Actinobacteria*. This suggests a mechanism of virulence evolution based on the cooption of existing core actinobacterial traits, triggered by key host niche–adaptive HGT events. We tested this hypothesis by investigating *R. equi* virulence plasmid-chromosome crosstalk, by global transcription profiling and expression network analysis. Two chromosomal genes conserved in environmental rhodococci, encoding putative chorismate mutase and anthranilate synthase enzymes involved in aromatic amino acid biosynthesis, were strongly coregulated with *vap* PAI virulence genes and required for optimal proliferation in macrophages. The regulatory integration of chromosomal metabolic genes under the control of the HGT–acquired plasmid PAI is thus an important element in the cooptive virulence of *R. equi*.

## Author Summary

*Rhodococcus* is a prototypic genus within the *Actinobacteria*, one of the largest microbial groups on Earth. Many of the ubiquitous rhodococcal species are biotechnologically useful due to their metabolic versatility and biodegradative properties. We have deciphered the genome of a facultatively parasitic *Rhodococcus*, the animal and human pathogen *R. equi*. Comparative genomic analyses of related species provide a unique opportunity to increase our understanding of niche-adaptive genome evolution and specialization. The environmental rhodococci have much larger genomes, richer in metabolic and degradative pathways, due to gene duplication and acquisition, not genome contraction in *R. equi*. This probably reflects that the host-associated *R. equi* habitat is more stable and favorable than the chemically diverse but nutrient-poor environmental niches of nonpathogenic rhodococci, necessitating metabolically more complex, expanded genomes. Our work also highlights that the recruitment or cooption of core microbial traits, following the horizontal acquistion of a few critical genes that provide access to the host niche, is an important mechanism in actinobacterial virulence evolution. Gene cooption is a key evolutionary mechanism allowing rapid adaptive change and novel trait acquisition. Recognizing the contribution of cooption to virulence provides a rational framework for understanding and interpreting the emergence and evolution of microbial pathogenicity.

## Introduction

*Rhodococcus* bacteria belong to the mycolic acid-containing group of actinomycetes together with other major genera such as *Corynebacterium*, *Mycobacterium* and *Nocardia* [1]. The genus *Rhodococcus* comprises more than 40 species widely distributed in the environment, many with biotechnological applications as diverse as the biodegradation of hydrophobic compounds and xenobiotics, the production of acrylates and bioactive steroids, and fossil fuel desulfurization [2]. The rhodococci also include an animal pathogen, *Rhodococcus equi*, the genome of which we report here.

*R. equi*, a strictly aerobic coccobacillus, is a multihost pathogen that causes purulent infections in various animal species. In horses, it is the etiological agent of “rattles”, a lung disease with a high mortality in foals [3]. *R. equi* lives in soil, uses manure as growth substrate, and is transmitted by the inhalation of contaminated soil dust or the breath of infected animals. Pathogen ingestion may result in mesenteric lymphadenitis and typhlocolitis, and multiplication in the fecal content of the intestine contributes to dissemination in the environment. *R. equi* causes chronic pyogranulomatous adenitis in pigs and cattle and severe opportunistic infections in humans, often in HIV-infected and immunosuppressed patients. Human rhodococcal lung infection resembles pulmonary tuberculosis and has a high case-fatality rate [3], [4].

*R. equi* parasitizes macrophages and, like *Mycobacterium tuberculosis* (Mtb), replicates within a membrane-bound vacuole. A 80–90 kb virulence plasmid confers the ability to arrest phagosome maturation, survive and proliferate in macrophages *in vitro* and mouse tissues *in vivo*, and to cause disease in horses. Virulence-associated protein A (VapA), a major plasmid-encoded surface antigen, is thought to mediate these effects [5]–[7]. The *vapA* gene is located within a horizontally-acquired pathogenicity island (PAI) together with several other *vap* genes [8]. Equine, porcine and bovine isolates carry specific virulence plasmid types differing in PAI structure and *vap* multigene complement, suggesting a role for *vap* PAI components in *R. equi* host tropism [8], [9].

Apart from the key role of the plasmid *vap* PAI, little is known about the pathogenic mechanisms of *R. equi*. We investigated the biology and virulence of this pathogenic actinomycete by sequencing an analysing the genome of strain 103S, a prototypic clinical isolate. With its dual lifestyle as a soil saprotroph and intracellular parasite, *R. equi* offers an attractive model for evolutionary genomics studies of niche breadth in *Actinobacteria*. The comparative genomic analysis of *R. equi* and closely related environmental rhodococi reported here provides insight into the mechanisms of niche-adaptive genome plasticity and evolution in this bacterial group. The *R. equi* genome also provides fundamental clues to the shaping of virulence in *Actinobacteria*.

## Results/Discussion

### General genome features

The genome of *R. equi* 103S consists of a circular chromosome of 5,043,170 bp with 4,525 predicted genes (Figure S1) and a circular virulence plasmid of 80,610 bp containing 73 predicted genes [8]. Overall G+C content is 68.76%. Table 1 summarizes the main features of the *R. equi* genome.

**Table 1:** General features of the genomes of *R. equi* 103S and the environmental species, *R. jostii* RHA1.

	Replicon	Size (bp)	Topology	GC %	No. of CDS	Pseudo-genes	Coding %	Coding density (average CDS length in bp)	rRNA clusters	tRNAs
***R. equi*** ** 103S**	Chromosome	5,043,170	Circular	68.82	4,525	14	90.3	0.89 (1009)	4	51
	pVAPA1037	80,610	Circular	64.61	73	8^a^	72.7	0.81 (901)	0	0
***R. jostii*** ** RHA1**	Chromosome	7,804,765	Linear	67.52	7,211	5	91.2	0.92 (987)	4	50
	pRHL1	1,123,075	Linear	65.05	1,146	2	82.1	1.02 (805)	0	2
	pRHL2	442,536	Linear	64.01	454	4	83.7	1.03 (816)	0	0
	pRHL3	332,361	Linear	64.91	334	0	84.9	1.00 (845)	0	0

See http://www.nite.go.jp/index-e.html and Table S3 for data from two other sequenced genomes from environmental *Rhodococcus* spp., *R. erythropolis* PR4 and *R. opacus* B4 (released online by NITE, the Japanese National Institute for Technology and Evaluation).

^**a**^Of which seven in the HGT *vap* PAI.

#### Comparative analysis

**Figure 1:**
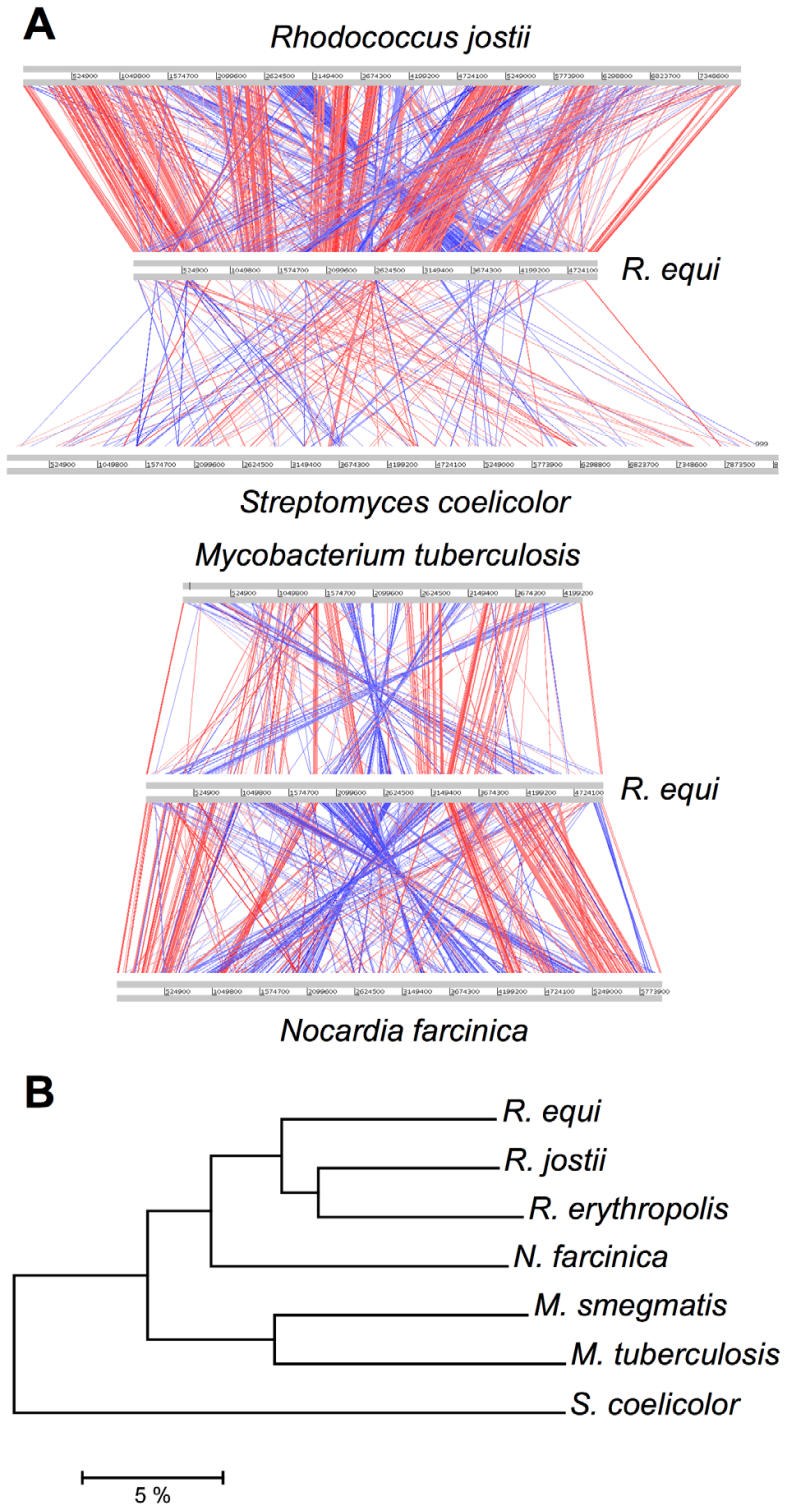
Comparative genomics and phylogenomics of *R. equi* 103S. (A) Pairwise chromosome alignments of *R. equi* 103S, *R. jostii* RHA1, *N. farcinica* IFM10152, *M. tuberculosis* (Mtb) H37Rv and *S. coelicolor* A3(2) genomes. Performed with Artemis Comparison Tool (ACT), see Table S12. Red and blue lines connect homologous regions (tBLASTx) in direct and reverse orientation, respectively. Mean identity of shared core orthologs between *R. equi* and: *R. jostii* RHA1, 75.08%; *N. farcinica*, 72.1%; Mtb, 64.6% (see also Figures S1, S2, and S5). (B) Phylogenomic analysis of *Rhodococcus* spp. and four other representative actinobacterial species. Unrooted neighbor-joining tree based on percent amino-acid identity of a sample of 665 shared core orthologs. The scale shows similarity distance in percentage.

Orthology analyses (Figure S1) and multiple alignments (Figure 1A) with representative published actinobacterial genomes showed the highest degree of homology and synteny conservation with *Rhodococcus jostii* RHA1 [10]. Next in overall genome similarity was *Nocardia farcinica*, followed by *Mycobacterium* spp., whereas *Streptomyces coelicolor* appeared much more distantly related, consistent with 16S rRNA-derived actinobacterial phylogenies. Some phylogenetic studies have been inconclusive, positioning *R. equi* either with the nocardiae or rhodococci [1], [11]. Our genome-wide comparative and phylogenomic analyses indicate this species is a *bona fide* member of the genus *Rhodococcus* (Figure 1, Figure S2).

Interestingly, *R. equi* has a substantially smaller genome than the soil-restricted versatile biodegrader *R. jostii* RHA1 (9.7 Mb) [10] and two recently sequenced environmental rhodococci, *Rhodococcus erythropolis* PR4 (6.89 Mb) and *Rhodococcus opacus* B4 (8.17 Mb) (see http://www.nite.go.jp/index-e.html). The rhodococcal genomes also differ in structure: *R. equi* and *R. erythropolis* have covalently closed chromosomes, whereas those of *R. jostii* and *R. opacus* are linear (Table 1, Figure S2). Chromosome topology does not seem to correlate with phylogeny, as *R. equi* and *R. erythropolis* belong to different subclades, and the latter is the prototype of the “*erythropolis* subgroup”, which includes *R. opacus* [11]. Streptomycetes also have large linear (>8.5 Mb) chromosomes [12], so linearization appears to have occurred independently in different actinobacterial lineages during evolution, apparently in association with increasing genome size.

#### Overview of functional content

The functional content of the *R. equi* 103S genome is summarized in Figure S3A. About one quarter of the genome corresponds to coding sequences (CDS) involved in central and intermediate metabolism (*n* = 1,108) and another quarter corresponds to surface/extracellular proteins (*n* = 1,073). “Regulators” is the next most populated functional category (*n* = 464, 10.3%). After adjusting for genome size, the number of membrane-associated proteins is average, but the regulome and secretome are clearly larger than in other *Actinobacteria* (Figure S4A, S4B, S4C), possibly reflecting specific needs associated with the habitat diversity of *R. equi*, from soil and feces to the macrophage vacuole. *R. equi* has 23 two-component regulatory systems, more than twice as many as host-restricted Mtb [13], and more regulators as a function of genome size than *S. coelicolor* [12] (Figure S4B). About 29% of the genome encodes products of unknown function. This percentage rises to 44.5% for secreted products (Figure S3B), 13% of which are unique to *R.equi*. Ortholog comparisons with representative closely related mycolata (*R. jostii*, *N. farcinica* and Mtb) showed *R. equi* to have the highest proportion of species-specific surface/extracellular proteins, consistent with its large secretome. By contrast, *R. jostii* RHA1 has the largest proportion of unique metabolic genes (Figure S5), consistent with its catabolic versatility [10]. Indeed, *R. jostii* RHA1 is unique among *Actinobacteria* in its unusual overrepresentation of metabolic genes (Figure S4D).

### Expansive evolution of rhodococcal genomes

The 5.0 Mb *R. equi* chromosome contains relatively few pseudogenes (*n* = 14, Table 1), most associated with horizontally acquired regions (*n* = 10, including two degenerate DNA mobility genes), consistent with a slow “core” gene decay rate. This suggests that the differences in chromosome size between rhodococci result mainly from genome expansion in environmental species rather than contraction in *R. equi*.

#### Gene duplication versus HGT

**Figure 2:**
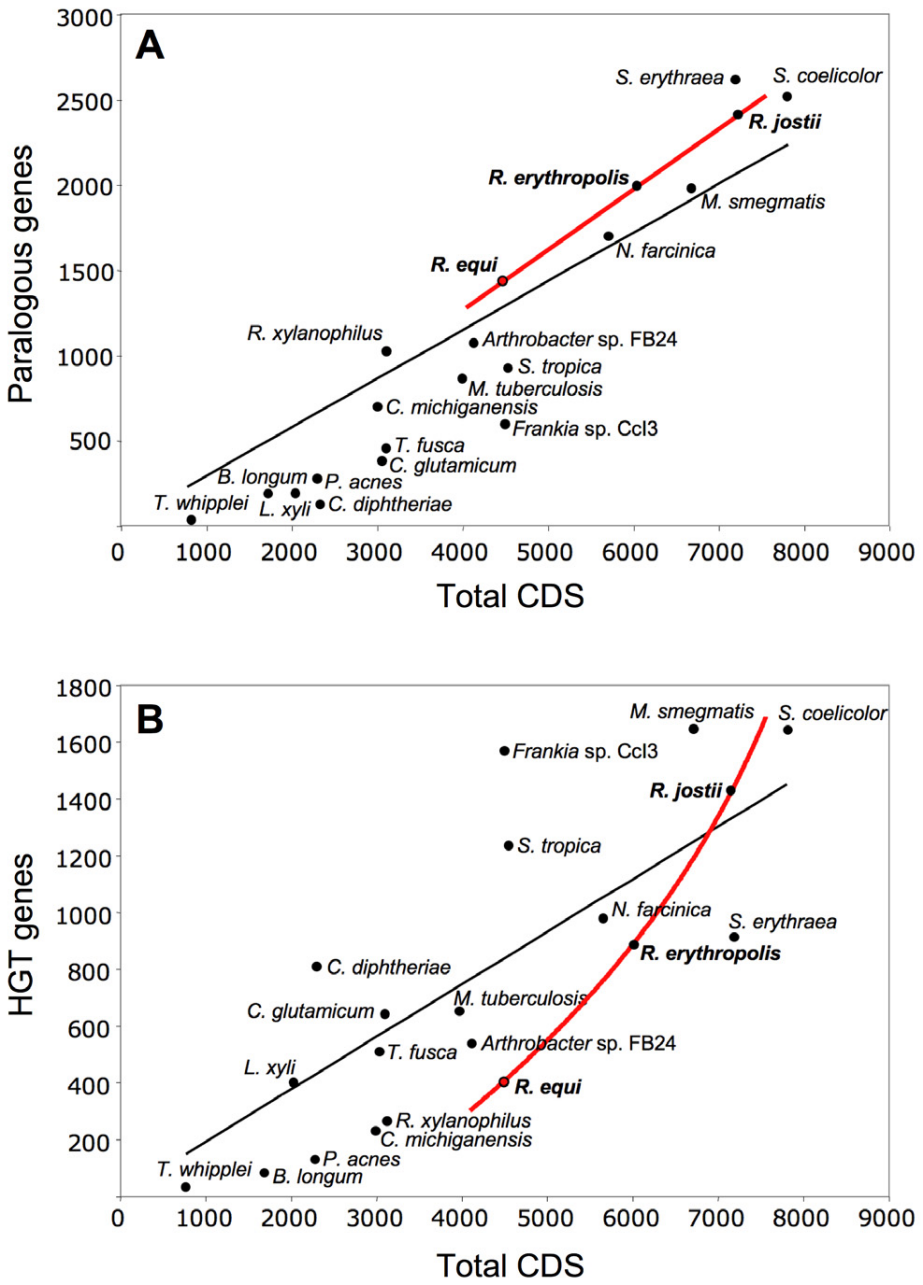
Role of gene duplication and horizontal gene transfer (HGT) in rhodococcal genome evolution. Scatter plots of (A) duplicated (paralogous) genes and (B) HGT genes versus the total number of genes in rhodococcal and actinobacterial genomes (curve fits of rhodococcal data in red, general trendline in black). HGT genes were excluded from the paralogy analyses.

We analyzed the paralogous families and local DNA compositional biases to assess the impact of gene duplication (GD) and horizontal gene transfer (HGT) in rhodococcal genome evolution (Tables S1, S2). As expected, both contributed to the chromosome size increase, but with different patterns: linear for GD (i.e. similar percentage of duplicated genes, 32.1, 33.2 and 33.6%, in *R. equi*, *R. erythropolis* and *R. jostii*, respectively), and exponential for HGT (9.5, 14.8 and 19.5%, respectively) (Figure 2). A possible explanation is that HGT involves the simultaneous acquisition of several genes (mean no. of genes per HGT “island” in rhodococci, 8.2 to 10.6). The probability of HGT in rhodococci also increases with chromosome size, as indicated by the mean frequencies of HGT events (1 every 87.0, 67.0 and 54.2 genes in *R. equi*, *R. erythropolis* and *R. jostii*, respectively) (Table S1). Moreover, recently acquired HGT islands, mostly containing “non-adapted” DNA dispensable in the short term in the new host species, are likely to evolve more freely and to tolerate further HGT insertions. This may be the case for two large chromosomal HGT “archipelagos” of ≈90 and 190 Kb in 103S, which probably were generated by an accumulation of HGT events. The mosaic structure of these HGT regions and the diversity of source species, as indicated by reciprocal BLASTP best-hit analysis, suggest that they are a composite of several independent HGT events rather than the result of a single “en-block” acquisition (Figures S1 and S6). Rhodococcal genome expansion also involves a linear increase in the number of paralogous families (with larger numbers of paralogs per family) and non-duplicated genes (Table S2), and an increasing number of unique hypothetical proteins (e.g. 164 in *R. equi*, 408 in *R. jostii*). Thus, genome expansion in rhodococci involves greater functional redundancy, diversity and innovation.

About 20% of *R. equi* HGT islands (Figure S1) are located close to tRNA genes, suggesting the involvement of phages or integrative plasmids in their acquisition. However, almost no DNA mobilization genes or remnants thereof were found associated with HGT regions, suggesting that the lateral gene acquisitions in the *R. equi* chromosome are evolutionarily ancient. Most HGT genes (52.5%) probably originated from other *Actinobacteria*, 3.5% of the best hits were from other bacteria, and 44% had no homologs in the databases. Only four integrase genes, one of them degenerate, and an IS1650-type transposase pseudogene were identified in the 103S chromosome. *R. equi* seems therefore to be genetically stable in terms of mobile DNA element-mediated rearrangements. DNA mobility genes —mostly associated with HGT regions and increasing in abundance with genome size— are more numerous in environmental rhodococci (Table S3). Thus, increasing genetic flux and plasticity are associated with increasing chromosome size in rhodococci.

#### Role of plasmids

Rhodococcal genome expansion can be largely attributed to extrachromosomal elements. *R. equi* has a single 80 Kb circular plasmid whereas environmental rhodococci have three to five plasmids, including large linear replicons up to 1,123 Kb in size, accounting for a substantial fraction of the genome (e.g. ≈20% in *R. jostii* RHA1) (Table 1, Table S3). Thus, as observed for chromosomal HGT DNA, the amount of plasmid increases exponentially with genome size. Indeed, one third of the plasmid DNA was HGT-acquired (32.4%, range 19.35–49.7 vs 14.5%, range 9.5–19.5 for the chromosomes), and plasmids may themselves be considered potentially mobilizable DNA. Rhodococcal plasmids also have a much higher density of DNA mobilization genes (Table S3), pseudogenes (Table 1), unique species-specific genes (mean 44.3±16.0% vs 3.6% to 5.6%), and niche-specific determinants (e.g. the intracellular survival *vap* PAI in *R. equi* [8] and 11 of the 26 peripheral aromatic clusters in *R. jostii* [10]) than the corresponding chromosomes. Rhodococcal plasmids are therefore clearly under less stringent selection and are key players in rhodococcal genome plasticity and niche adaptability.

### Niche-adaptive features

#### Basic nutrition and metabolism

No genes with an obvious role in carbohydrate transport were identified in 103S, consistent with the reported inability of *R. equi* to utilize sugars [14], confirmed here by Phenotype MicroArray (PMA) screens [15] and growth experiments in chemically defined mineral medium (MM) (Figure S7A). By contrast, *R. jostii*, *R. erythropolis* and *R. opacus* can grow on carbohydrates [16]–[18] and their genomes encode sugar transporters, including phosphoenolpyruvate-carbohydrate phosphotransferase system (PTS) permeases. Interestingly, the intracellular pathogens *R. equi*, Mtb and *Tropheryma whipplei* are the only mesophilic *Actinobacteria* lacking PTS sugar permeases (Table S4). However, Mtb grows on carbohydrates transported via non-PTS permeases. As the PTS is widespread in *Actinobacteria*, including nonpathogenic rhodococci and mycobacteria, the absence of PTS components in *R. equi*, Mtb and the genome-reduced obligate endocellular parasite *T. whipplei* probably results from gene loss.

The PMA and MM experiments showed that the only carbon sources used by *R. equi* 103S were organic acids (acetate, lactate, butyrate, succinate, malate, fumarate; but not pyruvate) and fatty acids (palmitate and the long-chain fatty acid-containing lipids Tween 20, 40 and 80) (Figure S7A). In addition to monocarboxylate and dicarboxylate transporters, the 103S genome encodes an extensive lipid metabolic network, with 36 lipases (16 of which secreted) and many fatty acid β-oxidation enzymes, with 40 acyl-CoA synthetases, 48 putative acyl-CoA dehydrogenases, and 23 enoyl-CoA hydratases/isomerases. Thus, *R. equi* seems to assimilate carbon principally through lipid metabolism. A mutant in the glyoxylate shunt enzyme isocitrate lyase (REQ38290) [19], required for anaplerosis during growth on fatty acids [20], has severely impaired intramacrophage replication and virulence [21], indicating that, as reported for Mtb [22], lipids are a major growth substrate for *R. equi* during infection *in vivo*.

**Figure 3:**
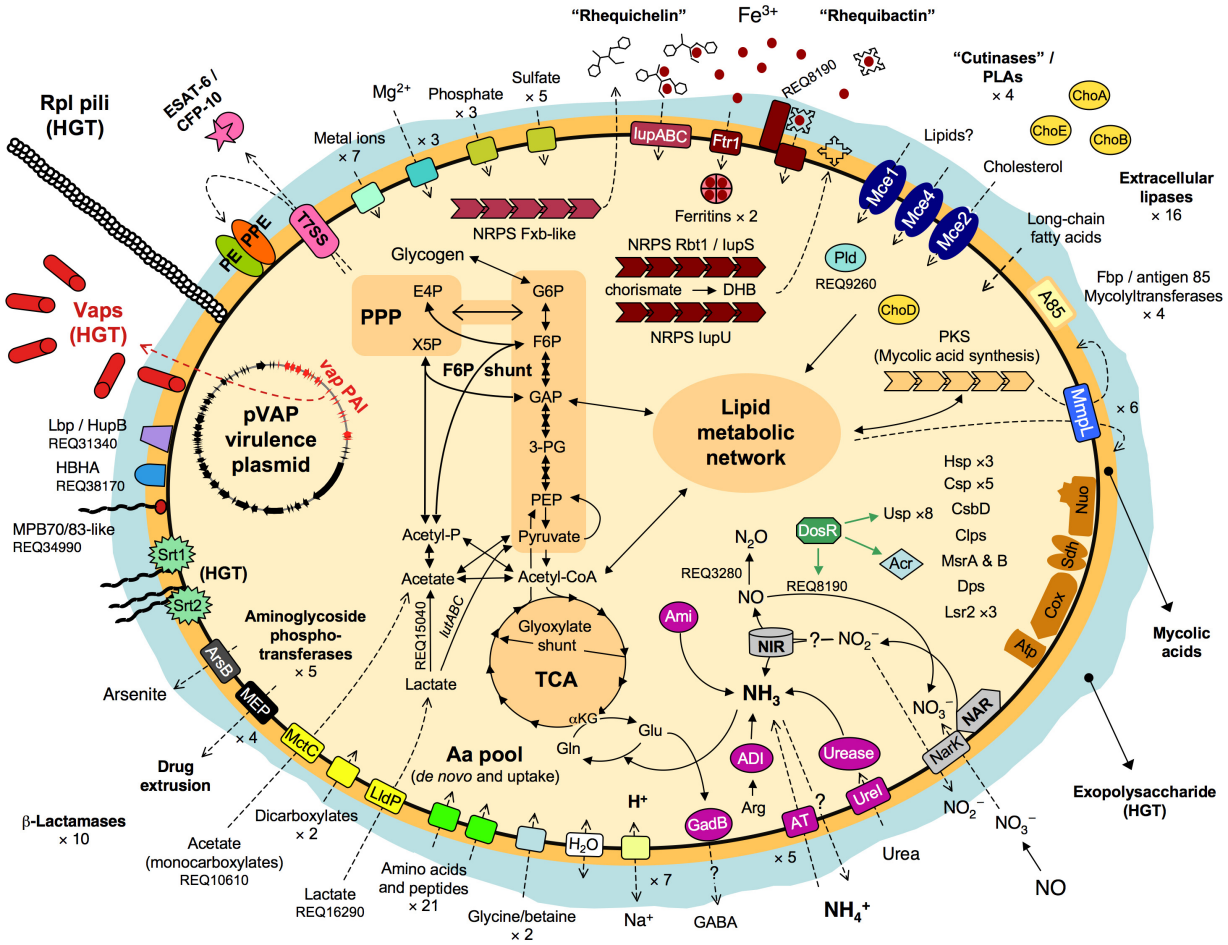
Schematic overview of relevant metabolic and virulence-related features of *R. equi* 103S. Complete glycolytic, PPP, and TCA cycle pathways, and all components for aerobic respiration, are present. The TCA cycle incorporates the glyoxylate shunt, which diverts two-carbon metabolites for biosynthesis. The methylcitrate pathway enzymes (*pprCBD*, REQ09040-60) are also present. The *lutABC* operon may take over the function of the D-lactate dehydrogenase (cytochrome) REQ00650, which is a pseudogene in 103S. REQ15040 (L-lactate 2-monoxygenase) and REQ27530 (pyruvate dehydrogenase [cytochrome]) can directly convert lactate and pyruvate into acetate. Unlike Mtb and other actinomycete pathogens, *R. equi* 103S has no secreted phospholipase C (Plc), only a cytosolic phospholipase D (Pld, REQ09260); a secreted Plc is however encoded in the genomes of environmental *Rhodococcus* spp. Rbt1/IupS (REQ08140-60) is a dimodular BhbF-like siderophore synthase [90]. Rbt1 rhequibactins are synthesized from (iso)chorismate via 2,3-dihydroxybenzoate (DHB) as for enterobactin or bacillibactin (REQ08130-100 encode homologs of Ent/DhbCAEB) [90], [91]. Two MFS transporters and a siderophore binding protein (REQ08180-200) encoded downstream from *iupS* may be involved in rhequibactin export/uptake. There is also a putative Ftr1-family iron permease (REQ12610). *R. equi* may store intracellular iron via two bacterioferritins (REQ01640-50) and the Dps/ferritin-like protein (REQ14900). IdeR- (REQ20130), DtxR- (REQ19260) and Fur- (REQ04740-*furA*, REQ29130-*furB*)-like regulators may contribute to iron/metal ion regulation. Homologs of the Mtb DosR (dormancy) regulon are also present in the *R. equi* genome (Table S6).

The 103S genome encodes 21 putative amino acid/oligopeptide transporters, and PMA screens and MM growth assays confirmed that *R. equi* uses several amino acids (tryptophan, tyrosine, phenylalanine, cysteine, methionine) and dipeptides as sources of nitrogen. However, 103S also has pathways for the *de novo* synthesis of all essential amino acids, consistent with the ability of *R. equi* to grow in MM containing only an inorganic nitrogen source (Figure S7A). Thus, *R. equi* can flexibly adapt to fluctuating conditions of amino-acid availability and grow in amino acid-deficient environments, as typically encountered in the infected host by intracellular pathogens [23]. See Figure 3 for a schematic overview of *R. equi* 103S nutrition and metabolism.

#### Thiamine auxotrophy

*R. equi* strains cannot grow without thiamine and an analysis of the loci involved in its biosynthesis revealed that *thiC* is absent from 103S, probably due to an HGT event affecting the *thiCD* genes (Figure S7B, S7C, S7D). The auxotrophic mutation is probably irrelevant for *R. equi* in the intestine and manure-rich soil owing to the availability of microbially synthesized thiamine. Host-derived thiamine is also probably available to *R. equi* during infection.

#### Specialized metabolism

We investigated the nutritional and metabolic aspects of rhodococcal niche adaptation by comparing the metabolic network of *R. equi* with that of *R. jostii* RHA1, the only other rhodococcal species for which a detailed manually annotated genome is available. RHA1 originated from lindane-contaminated soil and was identified by screening for biodegradative capabilities on multiple aromatic compounds, including polychlorinated biphenyls and steroids. Not surprisingly, its genome has an abundance of aromatic degradation pathways and oxygenases involved in aromatic ring cleavage [10]. *R. equi* is also soil-dwelling but is primarily isolated from clinical specimens and manure-rich environments, involving clearly different selection criteria and habitat conditions. We used reciprocal best-match BLASTP comparisons to identify the species-specific metabolic gene complements, in which the catabolic specialization is likely concentrated. The related pathogenic *Actinobacteria*, *N. farcinica* (which shares a dual soil saprophytic/parasitic lifestyle with *R. equi*) and Mtb (quasiobligate parasite) were also included in the analyses. *R. jostii* RHA1 contains a disproportionately larger number of unique metabolic genes than *R. equi*, *N. farcinica* and Mtb (*n* = 1,260 or 47.2% of total metabolic CDS vs only 326 to 375 or 22.9 to 29.2%, respectively) (Figure S8). The oversized metabolic network of RHA1 results from an expansion in the number and gene content of paralogous families (Table S5) and nonparalogous genes (643 CDS in RHA1 vs 209 to 288). Only three of the 29 aromatic gene clusters present in *R. jostii* [10] were identified in the 103S genome. *R. equi* therefore has a much smaller metabolic network than, and essentially lacks the vast aromatic catabolome of, *R. jostii* RHA1.

*R. equi* resembles other environmental *Actinobacteria* in being able to produce oligopeptide secondary metabolites. The 103S genome encodes 11 large non-ribosomal peptide synthetases (NRPS), including three involved in siderophore formation (see below). The only polyketide synthase (REQ02050) is involved in the synthesis of mycolic acids. By contrast, RHA1 has 24 NRPS and seven polyketide synthases [10]. Thus, genome expansion in *R. jostii* has been accompanied by an extensive amplification of secondary metabolism.

#### Other metabolic traits

*R. equi* reduces nitrates to nitrites [14] through a NarGHIJ nitrate reductase (REQ04200-30). There is also a NirBD nitrite reductase (REQ32900-30), a NarK nitrate/nitrite transporter (REQ32940) and a putative nitric oxide (NO) reductase (REQ03280) (Figure 3). *nirBD* is conserved in environmental rhodococci whereas *narGHIJ* and REQ03280 are not, indicating that *R. equi* is potentially well equipped for anaerobic respiration via denitrification, a useful trait for survival in microaerobic environments, as typically found in necrotic pyogranulomatous tissue [24], the intestine or manure. A *narG* mutation has been shown to attenuate *R. equi* virulence in mice [25], consistent with the bacteria encountering hypoxic conditions during infection, although this may also reflect defective nitrate assimilation *in vivo* [26].

Intriguingly, *R. equi* possesses a D-xylulose 5-phosphate (X5P)/D-fructose 6-phoshate (F6P) phosphoketolase (Xfp, REQ21880), the key enzyme of the “*Bifidobacterium*” F6P shunt, which converts glucose into acetate and pyruvate and is the main hexose fermentation pathway in bifidobacteria [27]. Unexpected fermentative metabolism has been detected in some strictly aerobic bacteria, such as *Pseudomonas* and *Arthrobacter* [28], but no NAD^+^ (anaerobic)-dependent lactate dehydrogenase or other obvious pyruvate fermentation enzyme was identified in 103S. As *R. equi* does not use sugars, a catabolic role for the F6P shunt is possible only if fed via gluconeogenesis/glycogenolysis. Alternatively, the F6P shunt may function in reverse (anabolic) mode in *R. equi*, in parallel to gluconeogenesis, directing excess acetate and glyceraldehyde-3-phosphate (GAP), generated from lipid metabolism, into the pentose phosphate pathway (PPP) (Figure 3). *R. equi* 103S has a *lutABC* operon (REQ16290-320), recently implicated in lactate utilization via pyruvate in *Bacillus* [29].

#### Alkaline optimal pH

*R. equi* tolerates a wide pH range, but growth is optimal between pH 8.5 and 10 (Figure S9). This alkaline pH is similar to that of untreated manure, potentially providing a selective advantage for colonization of the farm habitat. The 103S genome encodes a urease (REQ45360-410), an arginine deiminase (REQ11880), an AmiE/F aliphatic amidase/formamidase (REQ26530, next to REQ26520 encoding a UreI-like urea/amide transporter in an HGT island) and other amidases which, by releasing ammonia [30], may favor *R. equi* growth in acidic host habitats such as the macrophage vacuole (pH≤5.5), the airways or the intestine (typical pH values in horse, 5.3–5.7 and 6.4–6.7, respectively [31], [32]).

#### Stress tolerance

Like other soil bacteria [33], *R. equi* encodes a large number of σ factors (21 σ^70^) and stress proteins (e.g. eight universal stress family proteins [Usp], five cold shock proteins, three heat shock proteins and several Clp proteins). It also synthesizes the ppGpp alarmone involved in adaptation to amino acid starvation [34]. *R. equi* is transmitted by soil dust in hot, dry weather [3] and must therefore resist low water availability and desiccation-associated oxidative damage. There are two ABC glycine betaine/choline transporters (REQ00540-70 and REQ14620-60), an aquaporin (REQ29580), and genes for the synthesis of an exopolysaccharide (see below) and the osmolytes ectoine (*ectABC*, REQ07850), hydroxyectoine (*ectD*, REQ07850) and trehalose (REQ27400-30), potentially important for osmoprotection and water stress tolerance. *R. equi* is well equipped to face oxidative stress, with four catalases, four superoxide dismutases, six alkyl hydroperoxide reductases and two thiol peroxidases. It also synthesizes the unique actinobacterial redox-storage thiol compound, mycothiol [35], the antioxidant thioredoxin (REQ47340-50), and the protein-repairing peptide-methionine sulfoxide reductases MsrA (REQ01570) and MsrB (REQ20650) [36]. Three homologs of the virulence-associated mycobacterial histone-like protein Lsr2 [37] (one plasmid *vap* PAI-encoded [8], REQ03140 and 05980 chromosomal), and a Dps family protein [38] (REQ14900, cotranscribed with REQ14890 encoding a CsbD-like putative stress protein [39]), may protect against oxidative DNA damage. NO reductase REQ03280 and a putative NO dioxygenase (REQ10890) may confer resistance to nitrosative stress (Figure 3).

#### “Innate” drug resistance

*R. equi* 103S showed a degree of resistance to many antibiotics in the PMA screens, including 13 aminoglycosides, nine sulfonamides, six tetracyclines, 10 quinolones, 18 β-lactams and chloramphenicol. Standard susceptibility tests confirmed the resistance of 103S to a number of clinically relevant antibiotics (Table S7). This correlates with the presence in 103S of an array of antibiotic resistance determinants, including five aminoglycoside phosphotransferases, 10 β-lactamases and four multidrug efflux systems. Except for β-lactamase REQ26610, none of the resistance genes are associated with HGT regions or DNA mobility genes, suggesting they are ancient traits selected to confer resistance to naturally occurring antimicrobials rather than recent acquisitions associated with the medical use of antibiotics. Soil organisms tend to carry multiple drug resistance determinants [40], and homologs of most *R. equi* resistance genes are present in the genomes of environmental rhodococci, at the same chromosomal location in some cases (Figure S10).

### Virulence

Potential virulence-associated determinants were identified *in silico* based on (i) homology with known microbial virulence factors, (ii) literature mining for Mtb virulence mechanisms, (iii) automated genome-wide screening for virulence-associated motifs [41] and (iv) systematic inspection of HGT genes, the secretome, and of genes shared with pathogenic actinomycetes but absent from nonpathogenic species.

#### Mycobacterial gene families

The 103S genome harbors three complete *mce* (mammalian cell entry) clusters. Despite their name, the mechanisms by which these clusters contribute to mycobacterial pathogenesis remain unclear [42]. The *mce4* operon from *R. jostii* and its homolog *mce2* in *R. equi* have recently been shown to mediate cholesterol uptake, consistent with emerging evidence that *mce* clusters constitute a new subfamily of ABC importers [43], [44]. The recently reported lack of effect of an *mce2* mutation on *R. equi* survival in cultured macrophages [43] does not exclude a role in cholesterol utilization *in vivo* or in IFNγ-activated macrophages, as shown for an Mtb mutant in the homologous *mce* operon [45]. The surface-exposed PE and PPE proteins account for ≈7% of the coding capacity of the Mtb genome due to massive gene duplication, and are thought to play an important role in mycobacterial pathogenesis [46]. The *R. equi* genome also harbors PE/PPE genes, although only a single copy of each (Figure S11A). They lie adjacent in an operon (REQ01750-60) with the PE gene first, as frequently observed in Mtb, possibly reflecting the functional interdependence of the PE and PPE proteins [47]. REQ35460-550 is identical in structure to ESX-4, one of the five Mtb ESX clusters, and to the single ESX cluster present in *Corynebacterium diphtheriae*. ESX loci encode two small proteins, ESAT-6 (REQ35460) and CFP-10 (REQ35440), and their type VII secretion apparatus, which also mediates the export of PE and PPE proteins. ESAT-6 and CFP-10 form heterodimeric complexes and are major T-cell antigens and key virulence factors in Mtb [48]. *R. equi* possesses six *mmpL* genes, encoding members of the “mycobacterial membrane protein large” family of transmembrane proteins, which are involved in complex lipid and surface-exposed polyketide secretion, cell wall biogenesis and virulence [49]. There are also four Fbp/antigen 85 homologs (REQ01990, 02000, 08890, 20840), involved in Mtb virulence as fibronectin-binding proteins and through their mycolyltransferase activity, required for cord factor formation and integrity of the bacterial envelope [50].

#### Cytoadhesive pili

**Figure 4:**
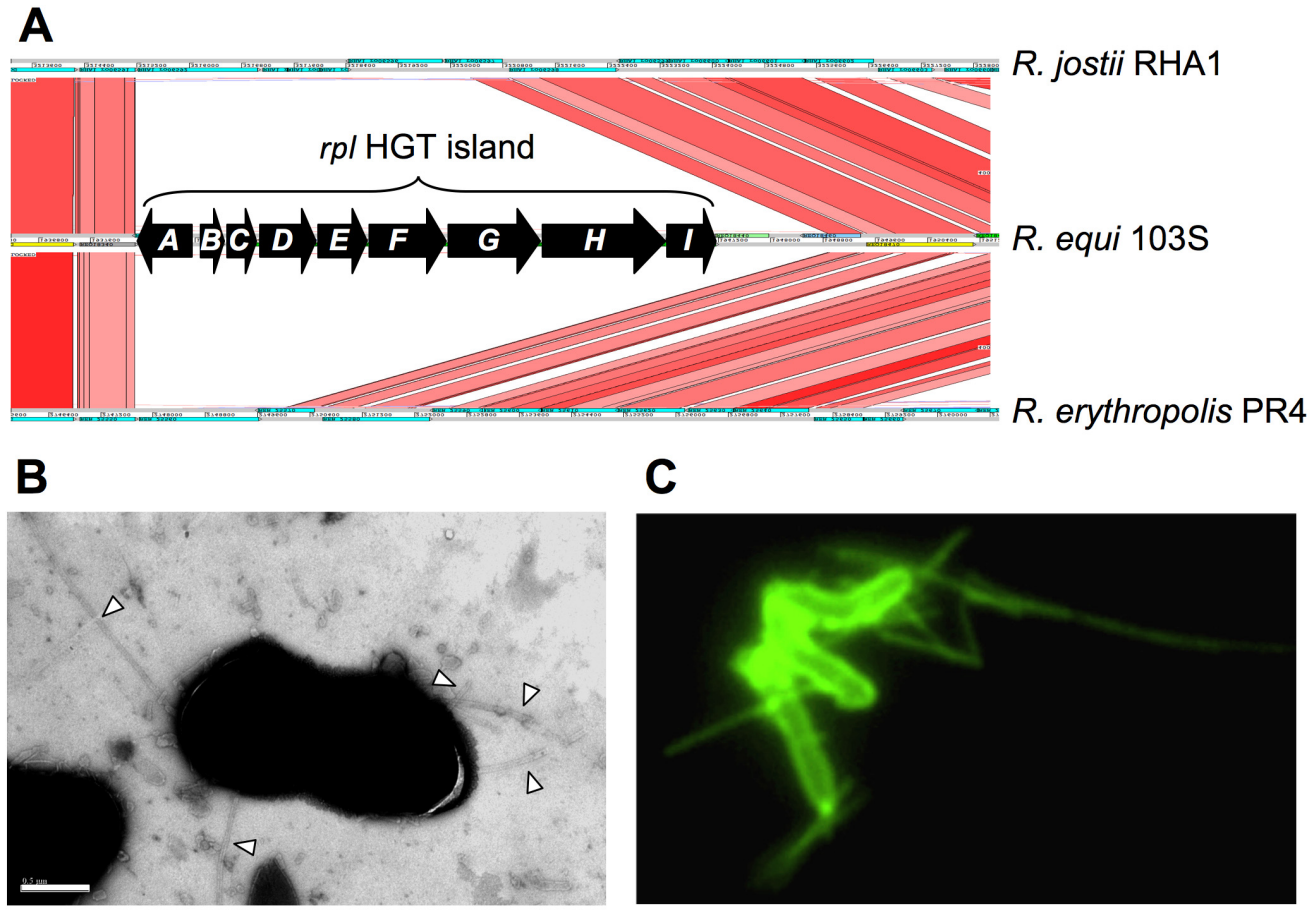
*R. equi* pilus locus (*rpl*). (A) The 9 Kb *rpl* HGT island (REQ18350-430) is absent from nonpathogenic *Rhodococcus* spp. *rpl* genes have been detected in all *R. equi* clinical isolates (P. Gonzalez *et al.*, manuscript in preparation). Putative *rpl* gene products: A, prepilin peptidase; B, pilin subunit; C, TadE minor pilin; D, putative lipoprotein; E, CpaB pilus assembly protein; F, CpaE pilus assembly protein; GHI, Tad transport machinery [51]. (B) Electron micrograph of *R. equi* 103S pili (indicated by arrowheads; generally 2–4 per bacterial cell). Bar = 0.5 µm. (C) *R. equi* 103S pili visualized by immunofluorescence microscopy (×1,000 magnification).

A nine-gene HGT island (REQ18350-430) encodes the biogenesis of Flp-subfamily type IVb pili, recently described in Gram-negative bacteria [51]. We confirmed the presence of pilus appendages in 103S (Figure 4). Gene deletion and complementation analysis demonstrated that the identified *R. equi* pili (Rpl) mediated attachment to macrophages and epithelial cells (P. González *et al.*, manuscript in preparation). The *rpl* island is absent from environmental rhodococci and is unrelated to the pilus determinants recently identified in Mtb and *C. diphtheriae* [52], [53].

#### Other putative virulence factors

*R. equi* is thought to produce capsular material [7], [54], and an HGT region encompassing REQ40580-780 contains genes potentially responsible for extracellular polysaccharide synthesis. Two other HGT islands encode sortases, transpeptidases that attach surface proteins covalently to the peptidoglycan and which are important for virulence in Gram-positive bacteria [55]. Both *srt* islands encode the putative substrates for the sortases (secreted proteins of unknown function) (Figure S11B).

Several secreted products are putative membrane-damaging or lipid-degrading factors, including a transmembrane protein with a putative hemolysin domain (REQ12980), three cholesterol oxidases (REQ06750, REQ26800, and REQ43910/ChoE [56]), four “cutinases”/serine esterases (REQ00480, REQ02020, REQ08540, REQ46060) with potential phospholipase A activity [57], and 16 lipases. REQ34990 encodes a secreted lipoprotein homologous to MBP70 and MPB83, two major mycobacterial antigens strongly expressed in *Mycobacterium bovis* BCG [58]. The REQ34990 product has a FAS1/BigH3 domain involved in cell adhesion via integrins [59]. There are also homologs of two mycobacterial cytoadhesins, the heparan sulfate-binding hemagglutinin HbhA (Rv0475) involved in Mtb dissemination (REQ38170), and the multifunctional histone-like/laminin- and glycosaminoglycan-binding protein Lbp/Hlp (REQ31340) [60] (Figure 3).

Iron is essential for microbial growth and the ability to acquire ferric iron from the host is directly related to virulence. Two NRPS, Rbt1/IupS (bimodular, REQ08140-60) and IupU (REQ23810), are involved in the formation of catecholic siderophores [61] or “rhequibactins”. A third NRPS homologous to *Mycobacterium smegmatis* Fxb (REQ07630) may be involved in the formation of an oligopeptide ferriexochelin-like extracellular siderophore. This “rhequichelin” is probably transported by the *iupABC* (REQ24080-100)-encoded putative siderophore ABC permease [61], homologous to the *M. smegmatis* FxuABC ferriexochelin transporter [62] (Figure 3). The redundancy of iron acquisition systems may explain the lack of effect on virulence of individual *iupU*, *rbt1*/*iupS* and *iupABC* mutations [61].

#### Virulence gene acquistion versus cooption

Only a few species-specific putative virulence loci were found in the 103S genome, all in HGT islands (e.g. the plasmid *vap* PAI or the chromosomal *rpl* locus). Most (≈90%) of the potential virulence-related determinants identified in *R. equi* were present in the environmental *Rhodococcus* spp. and/or had homologs in nonpathogenic *Actinobacteria* (Table 2, Table S8). These included orthologs of many experimentally-determined Mtb virulence genes, most of which (≈84%) are conserved among nonpathogenic mycobaceria or have close homologs in environmental actinomycetes (Table S9). The case of the *mce*, ESX, and PE/PPE loci is illustrative. Initially thought to be *Mycobacterium*-specific virulence traits, members of these multigene families are present in *R. equi* and in nonpathogenic rhodococci (Table S8), consistent with growing evidence that they are actually widely distributed among high-G+C gram-positives, whether environmental or pathogenic [42], [63], [64]. Notwithstanding that some of the unknown function genes of the 103S genome may encode novel, previously uncharacterized pathogenic traits, these observations are consistent with a scenario in which *R. equi* virulence largely involves the “appropriation” or cooption of core actinobacterial functions, originally selected in a non-host environment. Gene cooption (also known as preadaptation or exaptation) is a key evolutionary process by which traits that have evolved for one purpose are employed in a new context and acquire new roles, thus allowing rapid adaptive changes [65]–[67]. Cooptive evolution operates through critical modifications in gene expression and function [65]. These changes are particularly feasible in the larger genomes of soil bacteria, with a characteristic profusion of regulators and functionally redundant paralogs [68], [69]. Without the need for major changes, stress-enduring mechanisms and other housekeeping components, such as the cell envelope mycolic acids or the bacterial metabolic network, may directly contribute to virulence by affording nonspecific resistance or by enabling the organism to feed on host components. We suggest that a few decisive niche (host)-adaptive HGT events in a direct ancestor of *R. equi*, such as acquisition of the plasmid *vap* “intramacrophage survival” PAI [8] and the *rpl* “host colonization” HGT island (Figure 4), triggered the rapid conversion of a “preparasitic” commensal organism into a pathogen via the cooption of preexisting bacterial functions.

**Table 2:** Bacterial groups in which homologs of potential *R. equi* virulence-associated genes were identified.

Categories	No. of genes	%
*Actinobacteria* (shared by pathogenic and non-pathogenic spp.)	228	84.75
No significant match (*R. equi*-specific)	25^a^	9.29
*Rhodococcus* (non-pathogenic spp.)	10	3.71
Mtb and/or pathogenic mycobacteria, *Nocardia farcinica*	2	0.74
*Chloroflexi*	2	0.74
*Cyanobacteria*	1	0.37
*Proteobacteria*	1	0.37
Total	269^b^^,^ c	

Homology cutoff, ≥30% identity over 70% of sequence length. Mutually exclusive allocation to each category based on BLASTP best match. See Table S8 for complete list of genes.

**^a^**All in HGT islands, of which 76% in the virulence plasmid *vap* PAI.

**^b^**72.0% present in Mtb.

**^c^**50.9% of the encoded products are surface proteins/extracellular proteins.

### Virulence plasmid–chromosome crosstalk

Based on the well-established principle that coexpression with pathogenicity determinants is a strong indicator of involvement in virulence [70], [71], we sought to identify novel *R. equi* virulence-associated chromosomal factors through their coregulation with the plasmid virulence genes. The expression profiles of 103S and an isogenic plasmid-free derivative (103S^P−^) were compared, using a custom-designed genomic microarray and *in vitro* conditions known to activate (37°C pH 6.5) or downregulate (30°C pH 8.0) the virulence genes of the plasmid *vap* PAI [72], [73]. The plasmid had little effect on the chromosome in *vap* gene-downregulating conditions, but significantly altered expression was observed for numerous genes in *vap* gene-activating conditions (*n* = 88 with ≥2 fold change) (Table S10). Most of the differentially expressed genes (68%) were upregulated in the presence of the plasmid. These data suggest that the virulence plasmid activates the expression of a number of chromosomal genes, but whether this upregulation involves direct, specific (potentially virulence related) interactions or incidental pleiotropic effects is unclear.

#### Network analysis

To define the extent and nature of the virulence plasmid-chromosome crosstalk, we subjected the microarray expression data to network analysis. Unlike classical pairwise comparisons, the network approach captures higher-order functional linkages between genes, facilitating the graphic visualization of gene interconnections. It is thus more powerful for biological inference and gene prioritization for experimental validation. Noisy data also tend to be randomly distributed in the network structure [74]. We used BioLayout Express^3D^, an application that constructs three-dimensional networks from microarray data by measuring the Pearson correlation coefficients between the expression profiles of every gene in the dataset. This is followed by graph clustering using the Markov Clustering (MCL) algorithm to divide the network graph into discrete modules with similar expression profiles [75]. Microarray data of 103S bacteria exposed to various combinations of temperature (20°C, 30°C and 37°C) and pH (5.5, 6.5 and 8) were included in the computations to control for the excessive weight of the variable presence/absence of plasmid and strengthen the correlation analysis.

**Figure 5:**
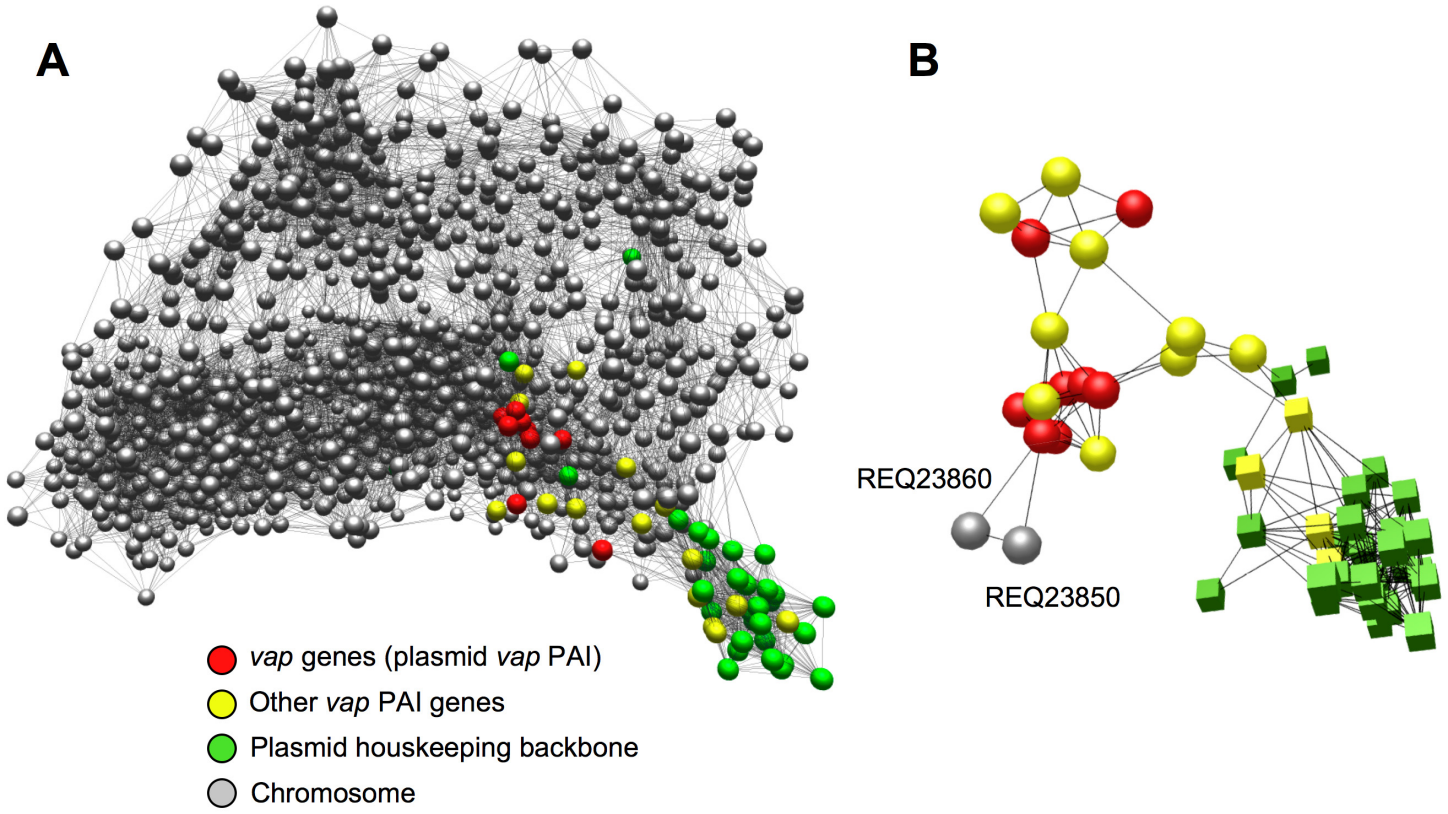
Network analysis of virulence plasmid–chromosome regulatory crosstalk. (A) Integration of the virulence plasmid *vap* PAI in the *R. equi* regulatory network. 3D graph of the *R. equi* 103S transcriptome (see text for experimental conditions) constructed with BioLayout Express^3D^, an application for the visualization and cluster analysis of coregulated gene networks [74], [75]. Settings used: Pearson correlation threshold, 0.85; Markov clustering (MCL) algorithm inflation, 2.2.; smallest cluster allowed, 3; edges/node filter, 10; rest of settings, default. Network graph viewable in Dataset S1. Each gene is represented by a node (sphere) and the edges (lines) represent gene expression interrelationships above the selected correlation threshold; the closer the nodes sit in the network the stronger the correlation in their expression profile. Note that the plasmid *vap* PAI genes (red spheres) are embedded within, and establish multiple functional connections with, chromosomal nodes (see also Figure S12A) whereas those of the plasmid housekeeping backbone lie outside the main network, reflecting an independent regulatory pattern. (B) Isolated subgraph of the *R. equi* transcription network obtained with *r* = 0.95 Pearson correlation threshold, showing the coregulation of the chromosomal genes REQ23860 (putative AroQ chorismate mutase) and REQ23850 (putative TrpEG-like bifunctional anthranilate synthase) (see Figure 7) with the virulence plasmid *vap* PAI genes. Color codes for nodes as indicated in (A) (spheres, *vap* PAI-coregulated cluster; cubes, plasmid housekeeping backbone cluster). MCL inflation, 2.2, smallest cluster allowed, 3; rest of settings, default. See Dataset S3.

Figure 5A shows a network representation of the functional connections detected in the *R. equi* transcriptome with a Pearson correlation threshold *r* ≥0.85. The graph model grouped the virulence plasmid genes into two distinct coregulated modules or clusters: one comprised 36 of the 73 plasmid genes, alsmost all from the housekeeping backbone (replication and conjugal transfer functions) [8]; the other contained 15 of the 26 *vap* PAI genes together with a number of chromosomal genes (Table S11). The plasmid housekeeping backbone nodes clustered together outside the main regulation network, reflecting functional independence from the rest of the regulome, as would be expected from the autonomous nature of the extrachromosomal replicon (Figure 5A). This indicates that the graph structure is biologically significant and reflects actual functional relationships, validating the network model. By contrast, the *vap* PAI nodes were clearly embedded in the network and established multiple connections with chromosomal nodes (Figure 5A, Figure S12A), suggesting that the plasmid virulence genes have undergone a process of regulatory integration with the host *R. equi* genome. About half of the predicted products of the chromosomal *vap* PAI-coregulated cluster genes are metabolic enzymes, the others being transcriptional regulators and transporters (Table S11C). Raising the correlation threshold to a highly stringent *r*≥0.95 disintegrated the network graph into a multitude of discrete, unconnected subgraphs (see Dataset S2). This did not substantially alter the structure of the two plasmid gene-containing clusters, but isolated two chromosomal genes, REQ23860 and REQ23850, as the most significantly and strongly coregulated with the *vap* PAI genes (Figure 5B), suggesting a direct regulatory interaction [76].

The genes from the plasmid backbone cluster were expressed constitutively in the conditions tested, whereas those from the *vap* PAI-coregulated cluster responded strongly to temperature, with activation at 37°C. Chromosomal genes in this cluster, particularly REQ23860 and REQ23850, displayed the same pattern, with downregulation in 103S^P−^ at 37°C, suggesting that plasmid factors are required for their induction at high temperature (Figure S12B, Table S11B). The *vap* PAI encodes two transcription factors, VirR (*orf4*) and an orphan two-component regulator (*orf8*) [8], both of which have been shown to influence *vap* gene expression [77], [78] and could be involved in the observed plasmid-mediated thermoregulation of the *vap* PAI-coexpressed cluster.

#### REQ23860 and REQ23850 are required for efficient intracellular proliferation in macrophages

**Figure 6:**
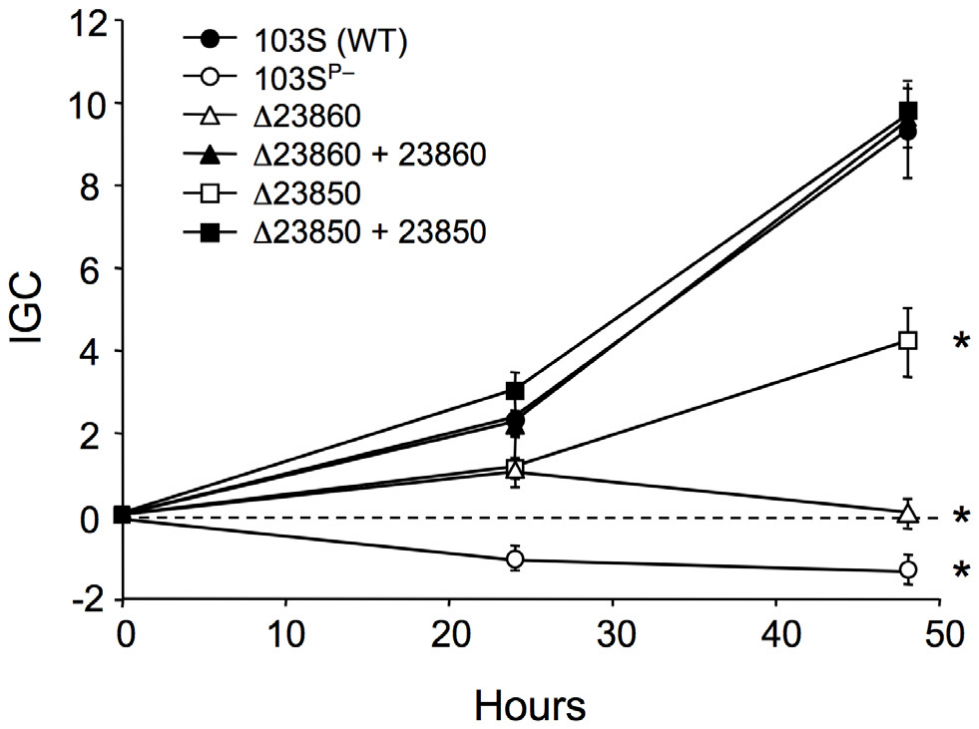
Intracellular growth kinetics of ΔREQ23860 and ΔREQ23850 mutants in J774 macrophages. Data were normalized to the initial bacterial counts at *t* = 0 using an intracellular growth coefficient (IGC); see Materials and Methods. Positive IGC indicates proliferation, negative values reflect decrease in the intracellular bacterial population. Bacterial counts per well at *t* = 0: 103S (wild type), 9.84±0.55×10^4^; 103S^P−^, 4.67±0.62×10^4^; ΔREQ23860 (putative CM), 11.26±2.78×10^4^; complemented ΔREQ23860, 4.24±0.10×10^4^; ΔREQ23850 (putative AS), 9.67±0.12×10^4^; complemented ΔREQ23850, 8.29±0.22×10^4^. Means of at least three independent duplicate experiments ±SE. Asterisks denote significant differences from wild type with *P*≤0.001 (two-tailed Student's *t* test). Except for the intracellular proliferation defect, the two mutants were phenotypically indistinguishable from the wild-type parental strain 103S, including growth kinetics in broth medium.

**Figure 7:**
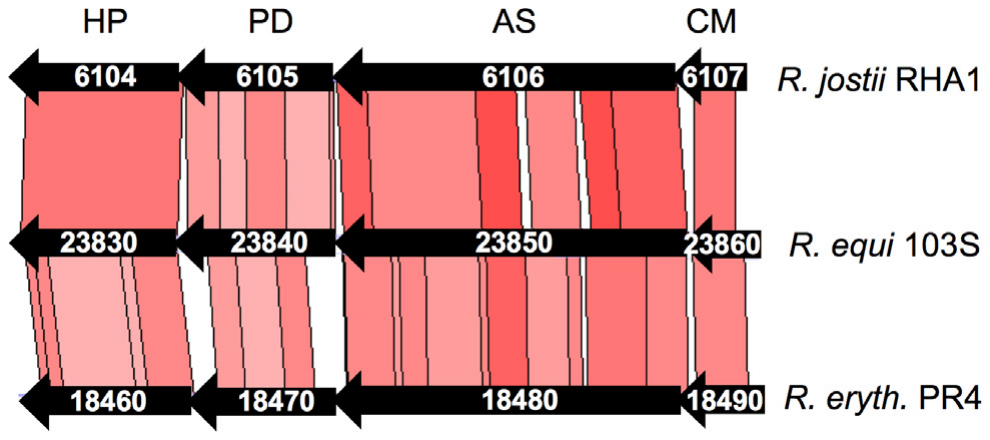
Structure of the chromosomal locus of the putative chorismate mutase (CM) and anthranilate synthase (AS) genes REQ23860 and REQ23850. The locus contains two additional genes, REQ23840 and REQ23830, encoding a putative prephenate dehydrogenase (PD) and a hypothetical protein (HP), respectively. The four genes are conserved at the same chromosomal location in the environmental *Rhodococcus* spp (CDS numbers indicated), including *R. opacus* B4.

REQ23860 and REQ23850 null mutants were constructed and tested in J774 macrophages to determine whether the observed coregulation with the plasmid *vap* PAI correlates with a role in virulence. The plasmidless derivative 103S^P−^, unable to proliferate intracellularly [5], was used as an avirulent control. The two mutants had a significantly attenuated capacity to grow in macrophages, restored to wild-type levels upon complementation with the deleted genes (Figure 6), indicating that REQ23860 and REQ23850 are required for optimal intramacrophage proliferation. The mutated genes encode an AroQ (type II) chorismate mutase (CM) and a bifunctional anthranilate synthase (AS) with fused TrpE and TrpG subunits, respectively, two key metabolic enzymes catalyzing the initial committed steps in aromatic amino-acid biosynthesis. CM generates prephenate, the first intermediate in the pathway leading to phenylalanine and tyrosine, whereas AS catalyzes the first reaction in tryptophan biosynthesis [79]. Downstream at the same locus, REQ23840 encodes a prephenate dehydrogenase (Figure 7), which catalyzes the oxidative decarboxylation of prephenate to the tyrosine precursor 4-hydroxyphenylpyruvate [79]. The intracellular growth defect caused by the mutations may therefore be related to a diminished capacity for *de novo* synthesis of aromatic amino acids. The *R. equi* genome encodes four other CM enzymes (including one in the *vap* PAI [8]) and an additional AS (bipartite, one subunit encoded in a *trpECBA* operon and the other by a solitary *trpG* gene elsewhere in the chromosome). Through their coregulation with the plasmid *vap* PAI, the redundant REQ23850-60-encoded chorismate-utilizing enzymes may be important for *R. equi* intracellular fitness and full proliferation capacity, by enhancing the *de novo* supply of aromatic amino acids, which generally appear to be present at limiting concentrations in the *in vivo* replication niche of intramacrophage vacuole-residing microbial pathogens [23], [80], [81].

### Conclusions

Somewhat counterintuitively for an organism with a dual lifestyle as a soil saprotroph and intracellular parasite, the *R. equi* genome is significantly smaller than those of environmental rhodococci. This may reflect that the main *R. equi* habitats –herbivore intestine, manure and animal tissues– provide a richer and more stable environment than the chemically diverse and probably nutrient-scarce environments of the nonpathogenic species. In nutrient-poor conditions, the simultaneous use of all available compounds as sources of carbon and energy may offer a competitive advantage, driving the selection of expanded genomes with greater metabolic versatility [10], [68]. Indeed, the much larger genome of the polychlorinated biphenyl-biodegrading *R. jostii* RHA1 encodes a disproportionately large metabolic network [10], with a wider diversity of paralogous families, unique metabolic genes and catabolic pathways. The relatively small number of pseudogenes and virtual lack of DNA mobilization genes in *R. equi* suggests that this species has not experienced a sudden evolutionary bottleneck with a concomitant relaxation of selective pressure and increase in mutation fixation [82]. The “coprophilic” and parasitic lifestyle specialization of *R. equi* seems to result from a “non-traumatic” adaptive process in an organism that, despite having suffered some specific functional losses (e.g. sugar utilization, thiamine synthesis), remains an “average” soil actinomycete with a normal-sized genome under strong selection. The greater genomic complexity of the environmental *Rhodococcus* spp. may reflect a “multi-substrate” niche specialization necessarily linked to the strict selection criteria —for unusual metabolic versatility— under which these species are generally isolated, [10]. Our analyses show that genome expansion in the environmental rhodococci has involved a linear gain of paralogous genes and an accelerated pattern of gene acquisition through HGT and extrachromosomal replicons, which evolve more rapidly and clearly play a critical role in rhodococcal niche specialization.

The lipophilic, asaccharolytic metabolic profile and capacity for assimilating inorganic nitrogen may be key traits for proliferation in herbivore intestine and feces, which are rich in volatile fatty acids [3], and in the macrophage vacuole and chronic pyogranulome, presumably poor in amino acids and rich in membrane-derived lipids [20], [23]. The potential for anaerobic respiration via denitrification may be critical for survival in the anoxic intestine or, as suggested for Mtb [83], [84], in necrotic granulomatous tissue. The inability to use sugars, unique among related actinomycetes, may confer a competitive advantage in the intestine and feces, dominated by carbohydrate-fermenting microbiota generating large amounts of short-chain fatty acids, which *R. equi* use as main carbon source. Alkalophily is probably an advantage in fresh manure, a major *R. equi* reservoir. *R. equi* is also well equipped to survive desiccation, important for dustborne dissemination in hot, dry weather, when rhodococcal foal pneumonia is transmitted [3], [4].

*R. equi* infections are notoriously difficult to treat due to the intracellular localization of the pathogen, compounded by a lack of susceptibility to antibiotics (e.g. penicillins, cephalosporins, sulfamides, quinolones, tetracyclines, clindamycin, and chloramphenicol) (Table S7 and refs. therein). With its panoply of drug resistance determinants, the 103S genome illustrates how naturally selected resistance traits, typically abundant in soil organisms, may have an important impact on the clinical management of microbial infections [40].

Finally, our analyses suggest that the appropriation of preexisting core actinobacterial components and functions are key events in the evolution of rhodococcal virulence. Although the underlying notion may be intuitively apparent when considering, for example, the contribution of housekeeping genes to bacterial virulence [85], here we are identifying it specifically as “gene cooption”, a key mechanism enabling rapid adaptive evolution and the emergence of new traits [65]–[67]. Underpinned by a few critical “host niche-accessing” HGT events, such as acquisition of the “intracellular survival” plasmid *vap* PAI or the “cytoadhesion” chromosomal *rpl* locus, this evolutionary mechanism is likely to have facilitated the rapid conversion of what was probably an animal-associated commensal into the pathogenic *R. equi*. Given the pervasive distribution of the “virulence-associated” gene pool among nonpathogenic species (Tables S8, S9), the notion of cooptive virulence is possibly applicable to all pathogenic actinomycetes and, indeed, universally to bacterial pathogens. The incorporation of adaptive changes in the regulation of the “appropriated” genes is a key mechanism in genetic cooption [65]. Our genome-wide microarray experiments and transcription network analyses indicate that the plasmid *vap* PAI, essential for intracellular survival and pathogenicity, has recruited housekeeping genes from the rhodococcal core genome under its regulatory influence. Among these are two chromosomal genes encoding key metabolic enzymes involved in aromatic amino-acid biosynthesis, coexpressed with the virulence genes of the *vap* PAI in response to an increase in temperature to 37°C (the body temperature of the warm-blooded host). These two metabolic genes are required by *R. equi* for full proliferation capacity in macrophages, providing supporting experimental evidence for the cooptive nature of *R. equi* virulence. A cooptive virulence model is consistent with the sporadic isolation of “nonpathogenic” (pre-parasitic) *Actinobacteria*, including environmental rhodococci (e.g. *R. erythropolis* [86]), as causal agents of opportunistic infections. An appreciation of the importance of gene cooption in the acquisition of pathogenicity provides a conceptual framework for better understanding and guiding research into bacterial virulence evolution.

## Materials and Methods

### Genome sequencing and analysis

We sequenced the original stock of the foal clinical isolate 103, designated clone 103S, to avoid mutations associated with prolonged subculturing *in vitro*. Strain 103 belongs to one of the two major *R. equi* genogroups (DNA macrorestriction analysis, unpublished data), is genetically manipulable, and is regularly used for virulence studies [25], [56]. Random genomic libraries in pUC19 were pair-end sequenced using dye terminator chemistry on ABI3700 instruments, with subsequent manual gap closure of shotgun assemblies and sequence finishing, as previously described [8]. The 103S genome sequence was manually curated and annotated with the software and databases listed in Table S12. A conservative annotation approach was used to limit informational noise [8]. For phylogenomic analyses, putative core ortholog genes were identified by reciprocal FASTA using a minimum cutoff of 50% amino acid similarity over 80% or more of the sequence. A similarity distance matrix was built with the average percentage amino acid sequence identity obtained by pairwise BLASTP comparisons (distance = 100 − average percent identity of 665 loci) and used to infer a neighbor-joining tree with the Phylip package [87]. The accession numbers of the genome sequences used in comparative analyses are listed in Table S13.

The sequence from the *R. equi* 103S genome has been deposited in the EMBL/GenBank database under accession no. FN563149.

### Phenotype analysis and microscopy

The nutritional and metabolic profile of *R. equi* 103S and its susceptibility to various drugs were analysed in Phenotype MicroArray screens (Biolog Inc., http://www.biolog.com) [15]. Substrate utilization was validated in supplemented mineral medium (MM) containing salts, trace elements, and ammonium chloride as the sole nitrogen source [19] (see Figure S7). For electron microscopy, a bacterial cell suspension in 0.1 M Tris-HCl (pH 7.5) was negatively stained with 1% uranyl acetate and observed at 80.0 kV in a Phillips CM120 BioTwin instrument (University of Edinburgh). Fluorescence microscopy was carried out on paraformaldehyde-fixed bacteria with an *R. equi* whole-cell rabbit polyclonal antiserum and Alexa Fluor 488-conjugated secondary antibodies (both diluted 1∶1000 in 0.1% BSA).

### Microarray expression profiling and network analysis

Total RNA was obtained from logarithmically growing *R. equi* bacteria (OD_600_ = 0.8) in Luria-Bertani (LB) medium, by homogenization in guanidinium thiocyanate-phenol-chloroform (Tri reagent, Sigma) with FastPrep-24 lysing matrix and a FastPrep apparatus (MP bio), followed by chloroform-isopropanol extraction, DNAase treatment (Turbo DNA-free, Ambion) and purification with RNeasy kit (Qiagen). RNA quantity and quality were determined with a Nanodrop (Thermo Scientific) and 2100 Bioanalyzer with RNA 6000 Nano assay (Agilent). RNA samples (500 ng) were amplified with the MessageAmp II-bacteria kit and 5-(3-amionallyl)-UTP (Ambion), labeled with Cy3 or Cy5 NHS-ester reactive dyes (GE Healthcare), and purified with RNeasy MinElute (Qiagen). Whole-genome 8×15K custom microarrays with up to four different 60-mer oligonucleotides per CDS (13,823 probes for the chromosome, 201 for the virulence plasmid) (Agilent) were hybridized in Surehyb DNA chambers (Agilent) with 300 ng of Cy3/Cy5-labeled aRNA, using Gene Expression Hybridisation and Wash Buffer kits (Agilent). Three experimental replicates per condition were analyzed, one with dye swap. The hybridization signals were captured and linear intensity-normalized, with Agilent's DNA microarray scanner and Feature Extraction software. Data were subsequently LOESS-normalized by intensity and probe location and analyzed with Genespring GX 10 software (Agilent). Network analysis of microarray expression data was carried out with Biolayout Express^3D^ 3.0 software [74], using log base 2 normalized ratios of Cy3/Cy5 signals and methods described in detail elsewhere [75]. Biolayout Express^3D^ is freely available at http://www.biolayout.org/.

### Mutant construction and complementation

In-frame deletion mutants of REQ23860 and REQ23850 were constructed by homologous recombination [56], using the suicide vector pSelAct for positive selection of double recombinants on 5-fluorocytosine (5-FC) [43]. Briefly, oligonucleotide primer pairs CMDEL1/CMDEL2 and CMDEL3/CMDEL4 were used for PCR amplification of two DNA fragments of ≈1.5 Kb corresponding to the seven 3′- and six 5′-terminal codons plus adjacent downstream and upstream regions of REQ23860. The CMDEL2 and CMDEL3 primers are complementary and were used to join the two amplicons by overlap extension. The PCR product carrying the ΔREQ23860 allele was inserted into pSelAct, using SpeI and XbaI restriction sites; the resulting plasmid was introduced into 103S by electroporation and transformants were selected on LB agar supplemented with 80 µg/ml apramycin. The same procedure was followed for ΔREQ23860, with primers ASDEL 1 to 4. Allelic exchange double recombinants were selected as previously described [43], [56]. For complementation, the REQ23860-50 genes plus the entire upstream intergenic region were amplified by PCR with CACOMP1 and 2 primers and stably inserted into the *R. equi* chromosome, using the integrative vector pSET152 [88]. PCR was carried out with high-fidelity PfuUltra II fusion HS DNA polymerase (Stratagene). The primers used are shown in Table S14.

### Macrophage infection assays

Low-passage (<20) J774A.1 macrophages (ATCC) were cultured in 24-well plates at 37°C, under 5% CO_2_ atmosphere, in DMEM supplemented with 2mM L-glutamine (Gibco) and 10% fetal bovine serum (Lonza) until confluence (≈2×10^5^ cells/well). J774A.1 monolayers were inoculated at 10∶1 MOI with washed *R. equi* from an exponential culture at 37°C in brain-heart infusion (BHI, OD_600_≈1.0). Infected cell monolayers were immediately centrifuged for 3 min at 172×g and room temperature, incubated for 45 min at 37°C, washed three times with Dulbecco's PBS to remove nonadherent bacteria, and incubated in DMEM supplemented with 5µg/µl vancomycin to prevent extracellular growth. After 1 h of incubation with vancomycin (*t* = 0) and at specified time points thereafter, cell monolayers were washed twice with PBS, detached with a rubber policeman and lysed by incutation for 3 min with 0.1% Triton X-100. Intracellular bacterial counts were determined by plating appropriate dilutions of cell lysates onto BHI. The presence of the virulence plasmid was checked by PCR on a random selection of colonies, using *traA*- and *vapA*- specific primers [9] to exclude the possibility of intracellular growth defects being due to plasmid loss. As the intracellular bacterial population at a given time point depends on initial numbers, bacterial intracellular kinetics data are expressed as a normalized “Intracellular Growth Coefficient” [89] according to the formula IGC = (IB*_t_*_ = *n*_−IB*_t_*_ = 0_)/IB*_t_*_ = 0_, where IB*_t_*_ = *n*_ and IB*_t_*_ = 0_ are the intracellular bacterial numbers at a specific time point, *t* = *n*, and *t* = 0, respectively.

## Supporting Information

Dataset S1 Layout file of expression network analysis with *r* = 0.85. Viewable with Biolayout Express 3D (http://www.biolayout.org/).
(0.34 MB ZIP)

Dataset S2 Layout file of expression network analysis with *r* = 0.95. Viewable with Biolayout Express 3D (http://www.biolayout.org/).
(0.06 MB ZIP)

Dataset S3 Layout file of expression network analysis with *r* = 0.95 (nodes not belonging to plasmid gene-containing clusters have been removed). Viewable with Biolayout Express 3D (http://www.biolayout.org/).
(0.03 MB ZIP)

Figure S1 Circular diagram of the *R. equi* 103S genome (chromosome and virulence plasmid). Outer two rings, coding sequences in the forward and reverse strand colored according to functional class (see Figure S3). Left, *R. equi* 103S chromosome with ortholog comparison and horizontally acquired (HGT) islands. Ortholog plots from 13 actinobacterial genomes are shown concentrically (outside to inside, from more to less related: *R. jostii* RHA1, *Nocardia farcinica* IFM10152, *Mycobacterium smegmatis* MC2 155, *Streptomyces coelicolor* A3(2), *Mycobacterium tuberculosis* H37Rv, *Arthrobacter* sp. FB24, *Corynebacterium glutamicum* ATCC 13032, *Thermobifida fusca* YX, *Frankia* sp. CcI3, *Corynebacterium diphtheriae* NCTC 13129, *Propionibacterium acnes* KPA171202, *Bifidobacterium longum* NCC2705 and *Tropheryma whipplei* TW08 27; see Table S13 for accession nos.). HGT DNA identified by Alien Hunter [92] is shown in red (HGT “archipelagos” 1 and 2 boxed; see Figure S6). The HGT islands tend to coincide with void areas in the ortholog plots, indicating they are species-specific DNA regions; note that they are regulary distributed across the genome. Inner plots: G+C % (gray) and G+C skew (violet/yellow, origin of replication is clearly detectable). Right, circular diagram of the pVAPA1037 virulence plasmid (not represented to scale); the *vap* PAI (HGT-acquired) is indicated by a thick black line. A detailed annotation and analysis of pVAP1037 has been published elsewhere [8].
(0.93 MB PDF)

Figure S2 Pairwise ACT alignments of rhodococcal chromosomes (*R. equi* 103S, *R. jostii* RHA1, *R. opacus* B4 and *R. erythropolis* PR4); see Figure 1A for interpretation. *R. opacus* has a large (7.25 Mb) linear chromosome like *R. jostii* (Table 1). The chromosome of *R. erythropolis* (6.52 Mb) is circular, as in *R. equi*. The four rhodococcal species sequenced to date share a common core of 2,674 orthologs. Mean identity of shared core orthologs between *R. equi* and: *R. opacus*, 75.08%; *R. erythropolis*, 73.8. Between *R. jostii* RHA1 and: *R. erythropolis* PR4, 76.88%; *R. opacus*, 94.87%. The chromosomes of *R. jostii* and *R. opacus* are highly homologous and syntenic and share 72% of the coding sequences (CDS). Based on the number of shared orthologs, average percent identity among shared core genes, and overall genome homology, *R. equi* appears to be phylogenetically equidistant to *R. erythropolis*, *R. jostii* and *R. opacus*, while the last two species are clearly very closely related (see also Figure 1B). *R. jostii* RHA1 genome published in [10], *R. opacus* B4 and *R. erythropolis* PR4 genomes published online by NITE, the Japanese National Institute for Technology and Evaluation (http://www.nite.go.jp/index-e.html; accession nos. in Table S13).
(3.23 MB PNG)

Figure S3 Functional classification of *R. equi* 103S genome. According to the Ecocyc classification scheme [93]. (A) Functional categories of *R. equi* 103S genes. “Surface/extracellular proteins” includes products with a signal sequence and/or transmembrane domain not allocated to another main functional category (e.g. central metabolism, degradation of small molecules, regulators, etc.). About 17% of *R. equi* CDSs correspond to “hypothetical proteins” or “conserved hypothetical” proteins. In addition to the 517 annotation entries as “putative membrane protein”, “integral membrane protein” or “secreted protein”, 28.5% of the *R. equi* genome products are of unknown function. (B) Functional categories of *R. equi* 103S secretome. The *R. equi* secretome comprises 736 CDSs, of which 44.5% encode proteins of unknown function, 20.3% correspond to transporters, 17.1% to lipoproteins, and 10.3% to extracellular enzymes possibly involved in nutrient breakdown and assimilation.
(0.17 MB PDF)

Figure S4 Scatter plots of selected functional categories vs genome size (≥4 Mb) of *R. equi* 103S and 10 other representative *Actinobacteria*. Data were inferred using the Comprehensive Microbial Resource (http://cmr.jcvi.org/) and the available genomes (Data Release 23.0). See Table S13 for accession nos. Membrane-associated and secreted proteins, as determined from TMHMM and SignalP outputs (see Materials and Methods). The number of regulators per genome has been calculated from keyword parsing of protein annotation. (A) Membrane-associated proteins. (B) Regulators. (C) Secreted proteins. (D) Metabolic proteins.
(0.11 MB PDF)

Figure S5 Species-specific gene complements of *R. equi* 103S, *R. jostii* RHA1, *N. farcinica* IFM10152, and *M. tuberculosis* H37Rv. The Venn diagram shows the number of chromosomal CDSs shared within a particular relationship (in brackets those unique to that relationship) as determined by ortholog comparisons (reciprocal FASTA best hits). Below the name of each species, the total number of genes in the genome is shown. The pie charts show the functional classification of the CDSs unique to each species and the shared common core.
(0.35 MB PDF)

Figure S6 Genetic structure of the two large chromosomal HGT regions in *R. equi* 103S. The position of these regions on the chromosome is indicated in Figure S1. Functional categories of the genes are indicated in color code as in Figure S3. Alien Hunter [92] HGT hits are indicated as black bars in the center. HGT region 1 (positions 1,684,996-1,775,619, REQ16110-770) encompasses 68 CDSs and is rich in genes encoding nucleases, helicases and restriction enzymes. HGT region 2 (positions 2,734,493-2,848,474, REQ25610-26970) encompasses 132 CDSs with a diversity of functional categories but mostly involved in metabolism. It also includes three of the 14 pseudogenes found on the *R. equi* 103S chromosome. The mosaic structure of these regions and the diversity of source species, as indicated by reciprocal BLASTP best-hit analysis, suggest they are a composite of several independent HGT events rather than the result of a single “en block” acquisition.
(1.14 MB PNG)

Figure S7 *R. equi* nutrition and metabolism. (A) Carbon source utilization. Growth assays of *R. equi* 103S in mineral medium (MM) [19] at 37°C. MM was supplemented (unless otherwise stated) with 20 mM of the indicated carbon sources and bacterial growth was monitored at OD_600_ every 30 min in a Fluostar Omega plate reader (BMG Labtech). Growth was detected only with lactate and acetate (mean of three experiments ±SD). Chemicals were purchased from Sigma. The nutritional and metabolic profile of *R. equi* (and its susceptibility to various chemicals and antibiotics) was initially investigated with Phenotype MicroArray (PMA) screens [15]. In the PMA plates PM1 and PM2 (carbon sources), certain substrates (e.g. glucose, arabinose, ribose, xylose, D-glucosamine, dihydroxyacetone and lyxose) sometimes give false positive results due to abiotic dye reduction (source: Michael Ziman, Biolog Inc). Experiments in MM confirmed that *R. equi* 103S does not utilize these substrates as sole carbon source. (B) ACT pairwise comparison of the thiamine biosynthesis gene clusters *thiCD* and *thiGSOE* in *R. equi* 103S and environmental rhodococci. In *R. equi*, the *thiC* gene has been replaced by an HGT region (black bar in the center) encoding proteins of unknown function. (C) Thiamine auxotrophy. Growth assay of *R. equi* 103S in 20 mM lactate MM medium. HMP, 4-amino-5-hydroxymethyl-pyrimidine phosphate (5% v/v of the crude preparation described in [94]). Negative control: no supplement. Most (∼80%) of the *R. equi* strains displayed thiamine auxotrophy. Experimental conditions as described in the legend to (A). (D) Diagram of the rhodococcal thiamine biosynthesis pathway. The *thiCD* genes are required for the production of 4-amino-5-hydroxymethyl-2-methylpyrimidine pyrophosphate; *thiGSOM* are involved in the generation of 4-methyl-5-(β-hydroxyethyl) thiazole phosphate, the second substrate required for the *thiE*-mediated synthesis of thiamine phosphate. Thiamine phosphate is ultimately phosphorylated by the product of the *thiL* gene to generate the biologically active thiamine pyrophosphate. As shown in (C), HMP did not support *R. equi* 103S growth, indicating that the thiamine biosynthetic pathway of *R. equi* 103S is also functionally affected downstream from *thiC*.
(0.61 MB PDF)

Figure S8 Species-specific metabolic gene complements of *R. equi* 103S, *R. jostii* RHA1, *N. farcinica* IFM10152, and *M. tuberculosis* H37Rv. Determined by ortholog comparison (reciprocal FASTA best hits). As the functional categories used for the annotation of the four genomes were not directly comparable, we first extracted the metabolism-related CDSs manually, on the basis of their predicted function. The Venn diagram shows the number of CDSs shared within a particular relationship (in brackets those unique to that relationship). Below the name of the species, the total number of metabolic genes present in the genome is shown. See Table S5 for paralogy analysis of the species-specific metabolic gene complements.
(0.36 MB PNG)

Figure S9 Optimal growth pH of *R. equi* 103S. Phenotype MicroArray [15] output of the relevant wells of plate PM10. Incubation was for 48 h at 37°C in an OmniLog instrument with readings taken every 15 minutes. Data were analyzed with OmniLog PM software. Consensus phenotypes for at least two replicas were determined based on the area difference under the kinetic curve of dye formation. Reported optimal pH values for other rhodococcal species: *R. imtechensis* 7.0 [95], *R. koreensis* 7.0–7.8 [96], *R. kroppenstedtii* 8.0 [97], *R. kunmingensis* 7.0–7.5 [98], *R. kyotonensis* 7.0 [99], *R. percolatus* 7.0–7.5 [100], *R. pyridinivorans* 7.5–8.5 [101], *R. tukisamuensis* 5.5–8.5 [102], *R. yunnanensis* 7.0–8.0 [103].
(0.23 MB PNG)

Figure S10 Examples of antibiotic resistance determinants located at the same chromosomal position in *R. equi* and two environmental *Rhodococcus* spp. Homologous resistance determinants indicated by yellow stripes in the ACT alignments.
(0.49 MB PNG)

Figure S11 Virulence-related loci of *R. equi* 103S. (A) PE/PPE locus and corresponding chromosomal regions in *R. jostii* RHA1, *R. erythropolis* PR4, *N. farcinica* IFM10152 and *M. tuberculosis* H37Rv. Arrows in ACT alignments indicate PE and PPE genes. The PE gene is of the “short” subclass (only a conserved N-terminal PE module of 99 to 102 residues); the PPE gene is of the “unique C-terminal domain” subclass [104]. The *R. equi* PE/PPE locus is inserted at the same chromosomal position in the nonpathogenic *Rhodococcus* spp. and in *N. farcinica*; no PE/PPE genes are present at the corresponding chromosomal region of Mtb, other mycobacteria and corynebacteria, indicating this PE/PPE locus is specific to the *Nocardiaceae* within the *Corynebacterinae*. The PE/PPE genes are fused in *R. jostii* RHA1. (B) Sortase HGT islands *srt1* and *srt2* of *R. equi* 103S. ACT comparisons of *srt1* (above) and *srt2* (below) and corresponding regions of *R. jostii* RHA1 and *R. erythropolis* PR4. Alien Hunter [92] outputs indicated as black bars in the center. *srt1* is unique to *R. equi* among the sequenced *Rhodococcus* spp., including *R. opacus* B4) (not shown). The *srt2* island is conserved in *R. erythropolis* but at a different chromosomal location and encoding only one of the two putative sortase substrates (surface protein RER_38400, which like its *R. equi* homolog REQ27480 contains an LPVTG sorting motif). Apart from a serine peptidase encoded by the *esx* locus (REQ35490), no proteins with the typical hallmarks of sortase substrates, i.e. a C-terminal membrane-spanning region preceded by a sortase recognition motif LPXTG, or a variant thereof) [105], are encoded outside the two *srt* islands.
(0.75 MB PDF)

Figure S12 Network analysis of *R. equi* microarray expression data. (A) Detail of the network graph of Figure 5A showing the web of functional linkages (edges) between the *vap* PAI-coregulated cluster (red nodes) and direct neighbor clusters (green nodes, plasmid backbone cluster; other clusters represented in different colors; individual directly connected nodes are in gray regardless of whether they belong to a larger cluster; chromosomal nodes are represented as spheres, plasmid nodes as cubes). All other nodes have been removed. Predominant functional classes among neighbor clusters (*n* = 129 nodes): Central and energy metabolism 27.1%, Membrane-associated/surface proteins/transporters 23.3%, Hypothetical proteins 18.6%, Regulators 9.3%, Degradation of small molecules 7.75%. Metabolism-related products encoded by direct neighbor nodes include enzymes of the shikimate pathway/biosynthesis of aromatic amino acids (prephenate dehydrogenase REQ02960, prephenate dehydratase REQ01720); porphyrin metabolism (magnesium chelatase REQ18110) and cobalamin biosynthesis (uroporphyrinogen-III C-methyltransferase REQ02960, CobB homolog REQ28830); synthesis of cysteine, activated sulfate (*cysB*, *D*, *G*, *K/M*, *Q* and *N/C* homologs); and mycothiol (mycothiol ligase MshC REQ22990), urease (UreA, C, D, F, and G homologs), and nitrite reductase NirB1 (REQ32930). (B) Representative expression profiles of the plasmid gene-containing clusters identified with *r* = 0.85 Pearson correlation threshold (see Table S11). Maroon lines, *vap* PAI-coregulated cluster (red and yellow nodes in Figure 5A); green lines, plasmid backbone cluster (green nodes in Figure 5A). The individual profiles of three biological replicates per test condition are plotted. Note that the *vap* PAI-coexpressed cluster, which includes chromosomal genes, is activated by both plasmid and temperature (37°C) whereas the plasmid backbone cluster is expressed constitutively in the same conditions. Common reference: average signal of 103S at 37°C pH 6.5.
(2.47 MB PNG)

Table S1 Statistics of horizontal gene acquisition (HGT) in actinobacterial chromosomes. HGT DNA was identified with the Alien Hunter program (http://www.sanger.ac.uk/Software/analysis/), which identifies horizontally acquired DNA by reliably capturing local compositional biases based on a variable-order motif distributions method [92]. The thick gray line delimits the genomes with chromosomes of less than and more than 4 Mb in size. Accession nos. of the genomes used are shown in Table S13.
(0.09 MB PDF)

Table S2 Chromosomal gene duplication and paralogous families in *R. equi* 103S and 19 other representative *Actinobacteria*. Paralogous families were identified by clustering of proteomes with BLASTClust (see Table S12).
(0.07 MB PDF)

Table S3 DNA mobility genes in *R. equi* 103S and environmental *Rhodococcus* spp genomes. Identified by keyword parsing of protein annotation; in brackets, genes associated with HGT regions. Plasmids from *R. erythropolis* PR4 published in [106].
(0.09 MB PDF)

Table S4 Phosphoenolpyruvate-sugar phosphotransferase system (PTS) components in a selection of actinobacterial genomes. Identified using motif search in Pfam database (Pfam motif identifiers indicated in footnotes).
(0.10 MB PDF)

Table S5 Ranking of the ten most populated paralogous metabolic gene families of *R. equi* 103S, *R. jostii* RHA1, *N. farcinica* IFM10152, and *M. tuberculosis* H37Rv. Determined by BLASTCLUST analysis. In brackets, number of paralogs within the family.
(0.09 MB PDF)

Table S6 Putative DosR/DevR boxes and corresponding transcriptional units in *R. equi* 103S ^a^. Identified with CLC Main Workbench (http://www.clcbio.com/) and the 20-bp consensus DosR/DevR box 5′-NNNGGGHCNWWNGNCCCBNN-3′ (N = any nucleotide, H = A/C/T, B = C/G/T, W = A/T) defined by Park et al. [70] and modified according to [107], [108]. Accuracy cutoff ≥85%, intergenic position relative to start codon ≤150 nt. The conserved DosR motif is boxed, the invariant G6 and C8 positions and matching nucleotides at the opposite half-site of the palindrome are shaded in black, deviations from the consensus motif are shown in lower case.
(0.12 MB PDF)

Table S7 Minimal inhibitory concentrations (MIC) of *R. equi* 103S to various antibiotics. Determined by the broth microdilution method. The data are consistent with previously reported antimicrobial susceptibility studies of *R. equi* isolates [111]–[116].
(0.06 MB PDF)

Table S8 Potential virulence-associated genes of *R. equi* 103S identified by bioinformatic mining of the genome and homologs in other pathogenic and nonpathogenic *Actinobacteria*.
(0.13 MB XLS)

Table S9 Experimentally determined virulence-associated genes of *M. tuberculosis* and homologs in nonpathogenic *Actinobacteria*.
(0.08 MB XLS)

Table S10 Virulence plasmid-chromosome crosstalk. Gobal microarray expression analysis of *R. equi* 103S and an isogenic plasmid-cured derivative (103S^P−^) during exponential growth in LB medium (OD_600_ = 0.8) in the indicated conditions (part A of table, 30°C-pH 8.0 = *vap* PAI gene-downregulating conditions; part B of table, 37°C-pH 6.5 = *vap* PAI gene-activating conditions [72], [73]). Chromosomal genes differentially expressed with *P*≤0.05 and fold-change cutoff ≥2 are listed. Expression data are presented as average fold-change of 103S relative to 103S^P−^; positive values indicate upregulation in the presence of the plasmid.
(0.12 MB PDF)

Table S11 Plasmid gene-containing coregulated clusters. Gene allocation defined by graph clustering of the transcription network shown in Figure 5A. (A) Plasmid backbone cluster. Shown for each gene, average pairwise comparison ratios of normalized microarray expression data from exponential cultures of *R. equi* 103S in LB medium (OD_600_ = 0.8) at 37°C relative to 20°C (pH 6.5). This cluster contains only plasmid genes, virtually all from the housekeeping backbone and mostly constitutively expressed in the experimental conditions tested (see Figure S12B). (B) Same information as in (A) but for the plasmid *vap* PAI-coexpressed cluster ^a^, in the indicated conditions. P versus NP, pairwise comparison of *R. equi* 103S and its isogenic plasmidless derivative 103S^P−^ in *vap* gene-activating conditions [72], [73]. In bold, fold change differences ≥1.5 and P≤0.05. (C) Short list of *vap* PAI-coexpressed chromosomal genes and putative functions. Genes from part B not showing significant differential regulation by both temperature (at least one experimental condition) and plasmid in pairwise comparisons have been excluded (fold-change ≥1.5, P≤0.05 two-tailed Student's t test).
(0.16 MB PDF)

Table S12 Software and databases used to annotate and analyze the *R. equi* 103S genome.
(0.06 MB PDF)

Table S13 GenBank accession nos. of the genomes used in this study. *R. erythropolis* PR4 and *R. opacus* B4 genomes published online by NITE, the Japanese National Institute for Technology and Evaluation (http://www.nite.go.jp/index-e.html).
(0.08 MB PDF)

Table S14 Oligonucleotide primers used for mutant construction and complementation. SpeI, XbaI and EcoRV restriction sites used for the cloning of PCR products are underlined.
(0.05 MB PDF)
