# Supplementary figures and images for: The Genome of a Pathogenic Rhodococcus: Cooptive Virulence Underpinned by Key Gene Acquisitions
Source: PLoS Genet. 2010 Sep 30;6(9):e1001145. doi: 10.1371/journal.pgen.1001145 (PMC2947987; doi:10.1371/journal.pgen.1001145)

Fig S1

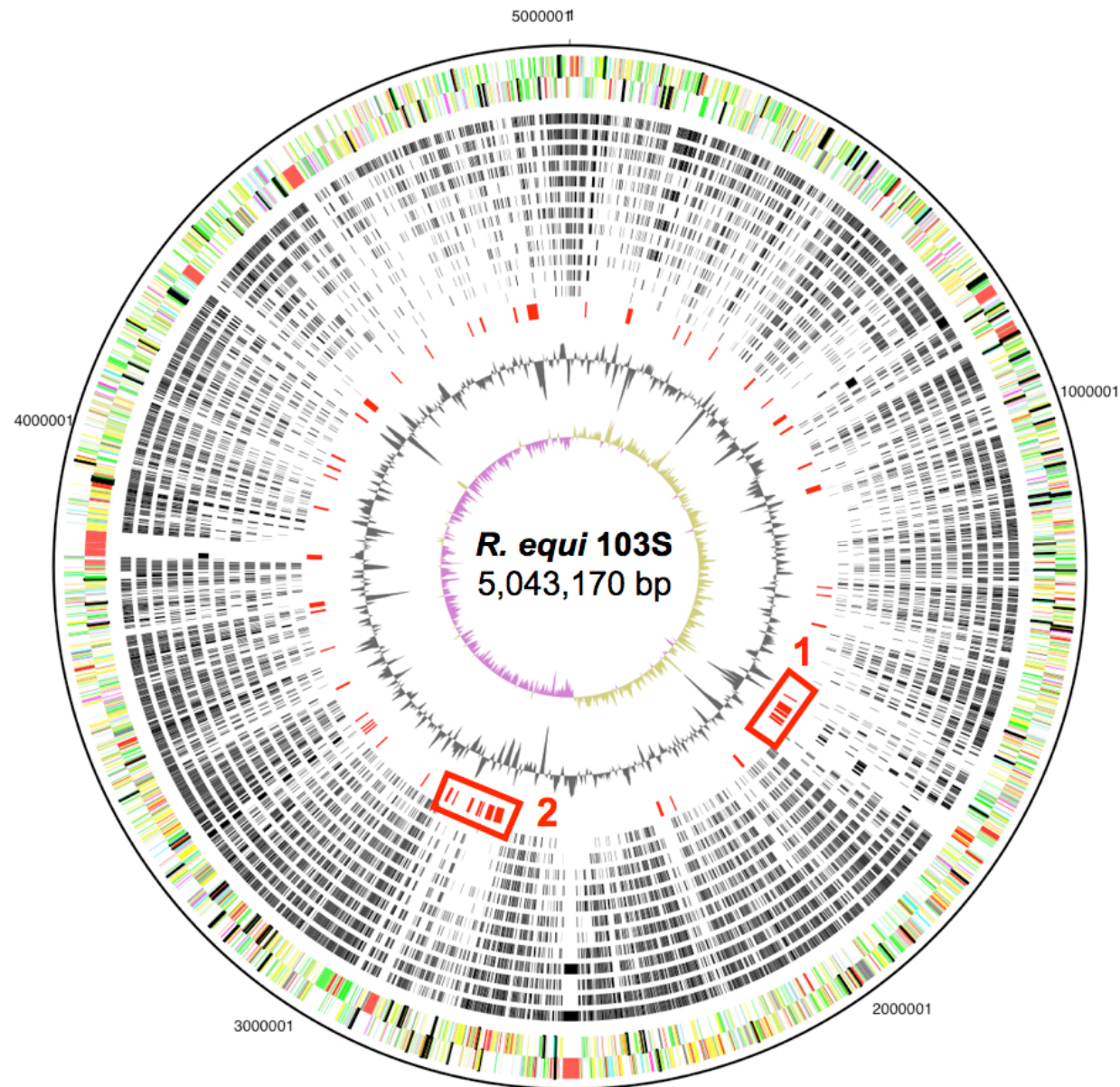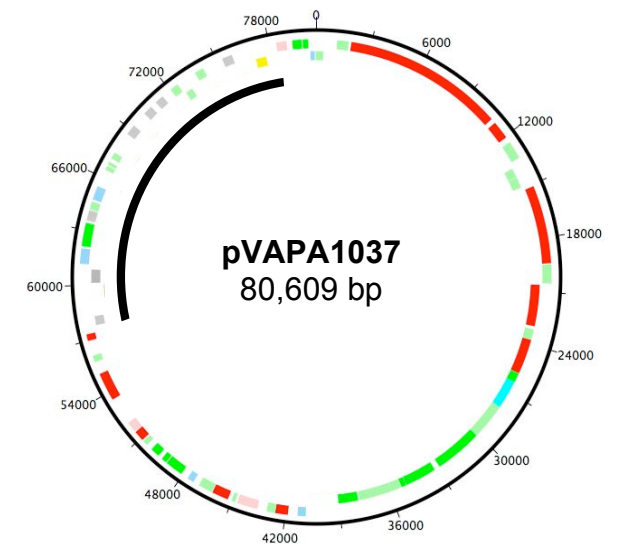

Supplement: Figure S1 — Circular diagram of the R. equi 103S genome (chromosome and virulence plasmid). Outer two rings, coding sequences in the forward and reverse strand colored according to functional class (see Figure S3). Left, R. equi 103S chromosome with ortholog comparison and horizontally acquired (HGT) islands. Ortholog plots from 13 actinobacterial genomes are shown concentrically (outside to inside, from more to less related: R. jostii RHA1, Nocardia farcinica IFM10152, Mycobacterium smegmatis MC2 155, Streptomyces coelicolor A3(2), Mycobacterium tuberculosis H37Rv, Arthrobacter sp. FB24, Corynebacterium glutamicum ATCC 13032, Thermobifida fusca YX, Frankia sp. CcI3, Corynebacterium diphtheriae NCTC 13129, Propionibacterium acnes KPA171202, Bifidobacterium longum NCC2705 and Tropheryma whipplei TW08 27; see Table S13 for accession nos.). HGT DNA identified by Alien Hunter [92] is shown in red (HGT “archipelagos” 1 and 2 boxed; see Figure S6). The HGT islands tend to coincide with void areas in the ortholog plots, indicating they are species-specific DNA regions; note that they are regulary distributed across the genome. Inner plots: G+C % (gray) and G+C skew (violet/yellow, origin of replication is clearly detectable). Right, circular diagram of the pVAPA1037 virulence plasmid (not represented to scale); the vap PAI (HGT-acquired) is indicated by a thick black line. A detailed annotation and analysis of pVAP1037 has been published elsewhere [8]. (0.93 MB PDF) [file pgen.1001145.s004.pdf]

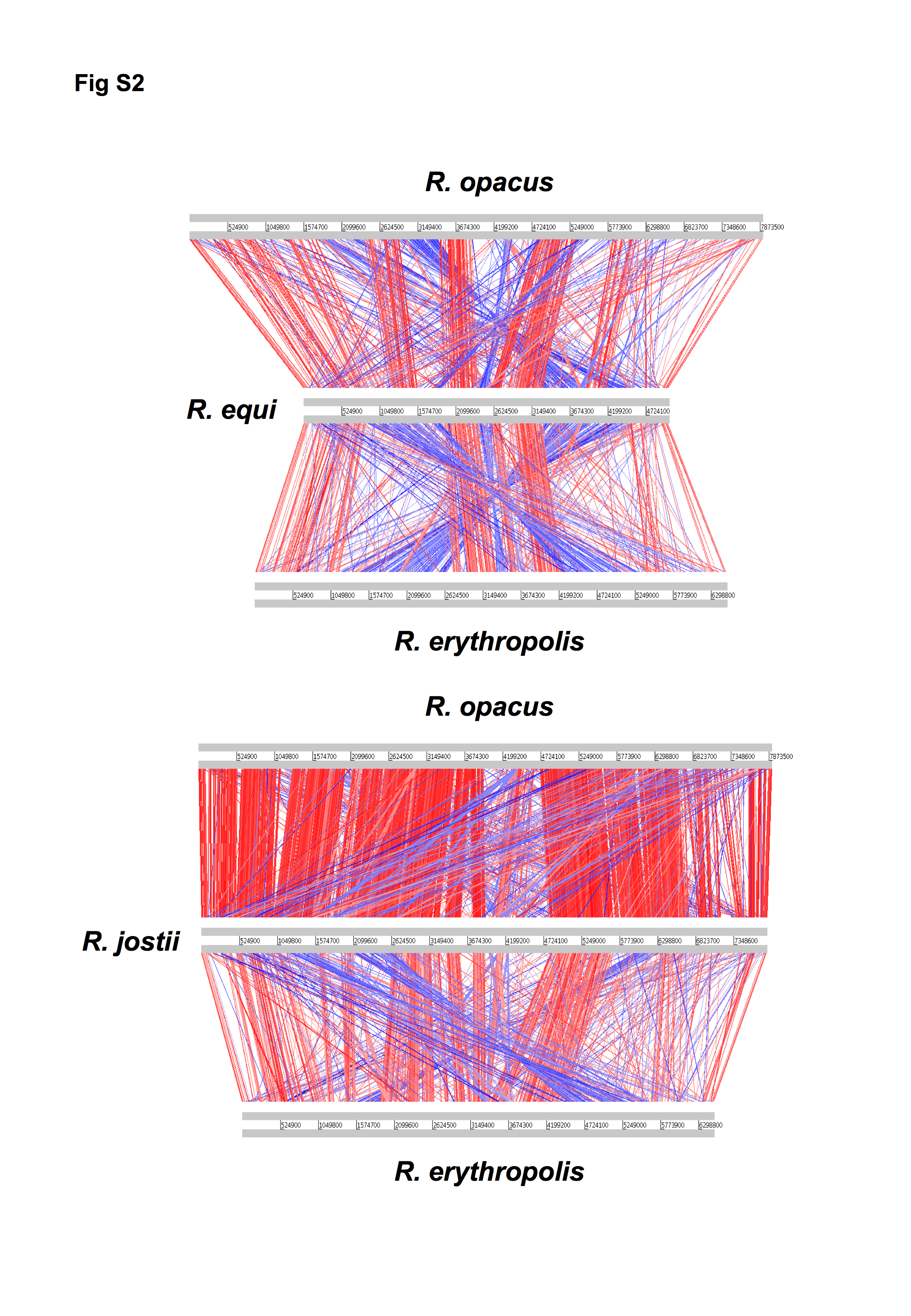

Supplement: Figure S2 — Pairwise ACT alignments of rhodococcal chromosomes (R. equi 103S, R. jostii RHA1, R. opacus B4 and R. erythropolis PR4); see Figure 1A for interpretation. R. opacus has a large (7.25 Mb) linear chromosome like R. jostii (Table 1). The chromosome of R. erythropolis (6.52 Mb) is circular, as in R. equi. The four rhodococcal species sequenced to date share a common core of 2,674 orthologs. Mean identity of shared core orthologs between R. equi and: R. opacus, 75.08%; R. erythropolis, 73.8. Between R. jostii RHA1 and: R. erythropolis PR4, 76.88%; R. opacus, 94.87%. The chromosomes of R. jostii and R. opacus are highly homologous and syntenic and share 72% of the coding sequences (CDS). Based on the number of shared orthologs, average percent identity among shared core genes, and overall genome homology, R. equi appears to be phylogenetically equidistant to R. erythropolis, R. jostii and R. opacus, while the last two species are clearly very closely related (see also Figure 1B). R. jostii RHA1 genome published in [10], R. opacus B4 and R. erythropolis PR4 genomes published online by NITE, the Japanese National Institute for Technology and Evaluation (http://www.nite.go.jp/index-e.html; accession nos. in Table S13). (3.23 MB PNG) [file pgen.1001145.s005.png]

Fig S3

A

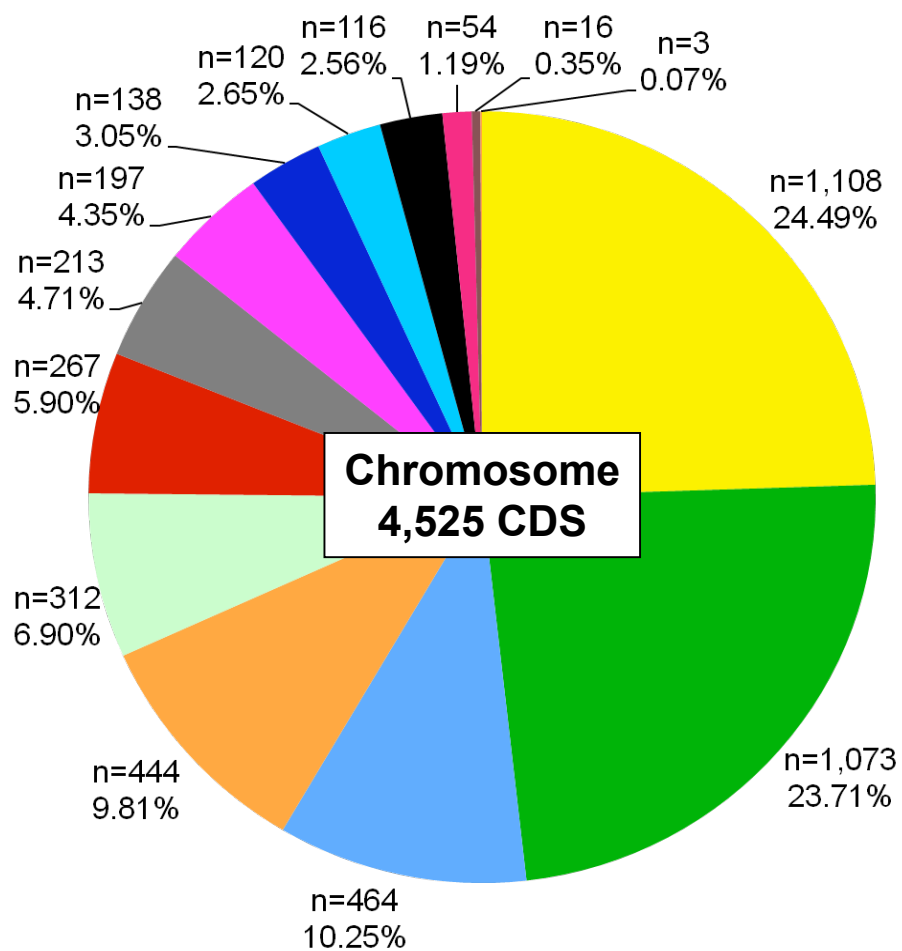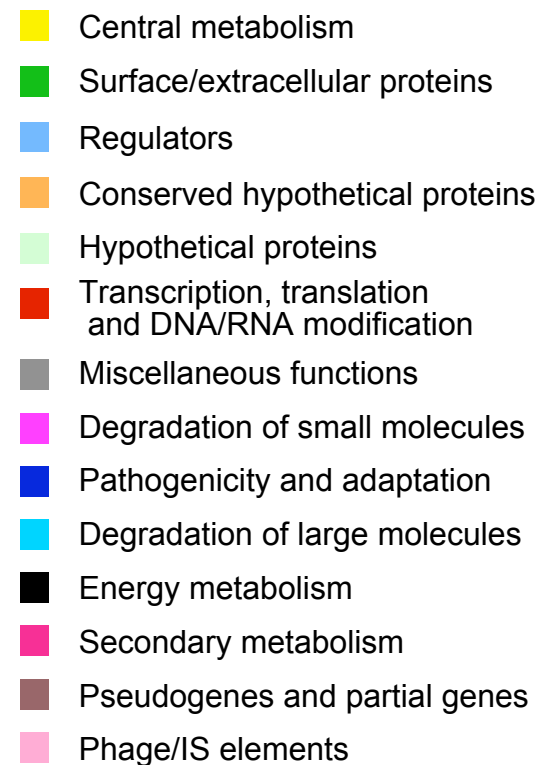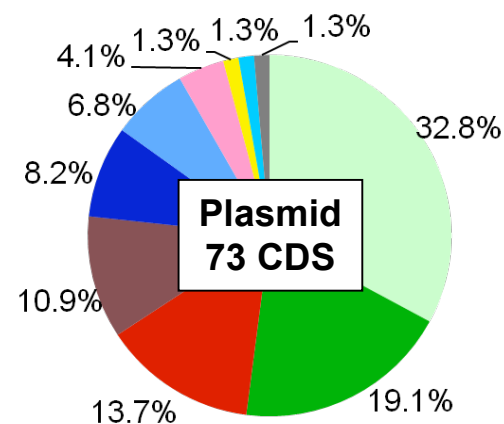

**B**

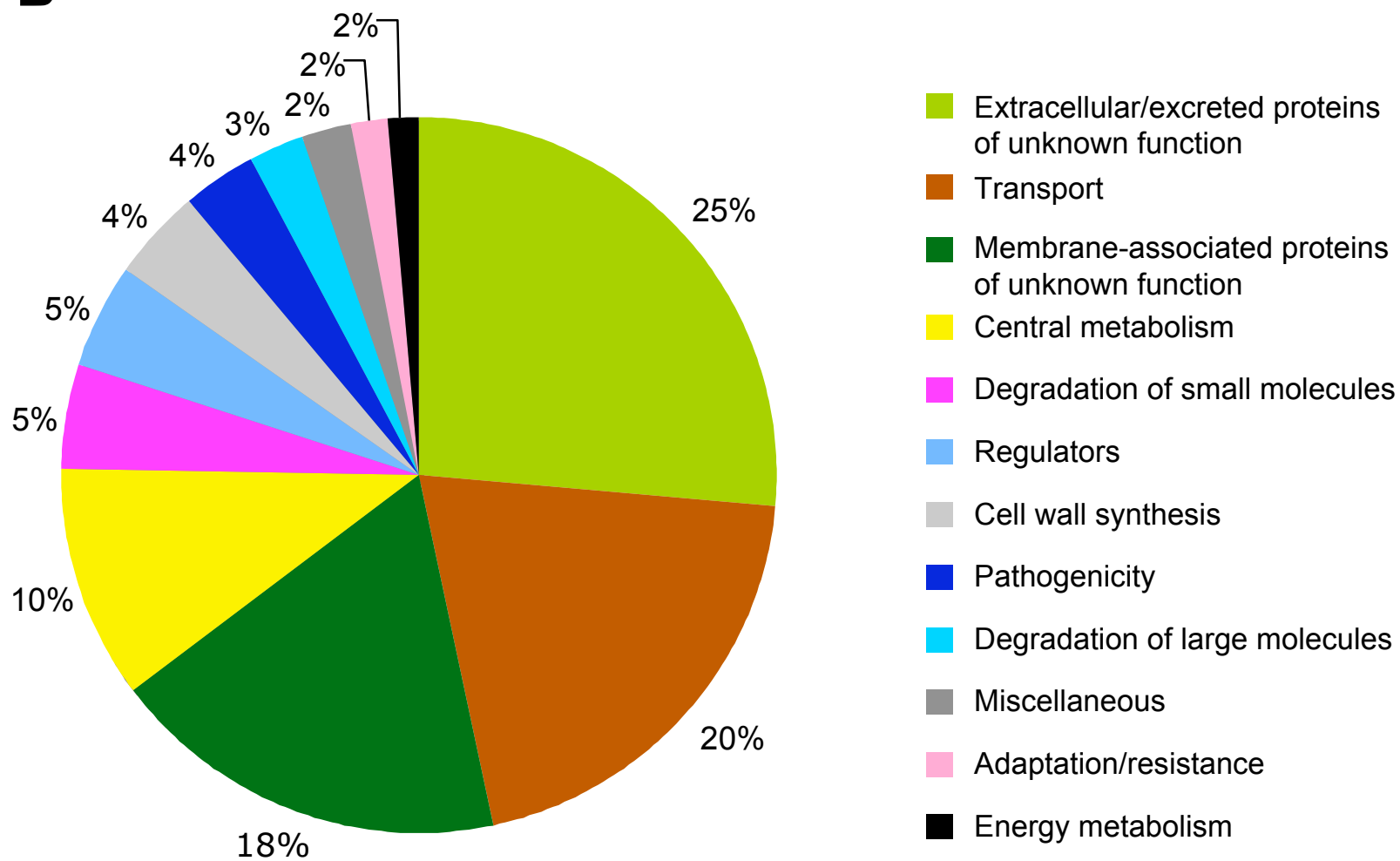

*R. equi* secretome

Supplement: Figure S3 — Functional classification of R. equi 103S genome. According to the Ecocyc classification scheme [93]. (A) Functional categories of R. equi 103S genes. “Surface/extracellular proteins” includes products with a signal sequence and/or transmembrane domain not allocated to another main functional category (e.g. central metabolism, degradation of small molecules, regulators, etc.). About 17% of R. equi CDSs correspond to “hypothetical proteins” or “conserved hypothetical” proteins. In addition to the 517 annotation entries as “putative membrane protein”, “integral membrane protein” or “secreted protein”, 28.5% of the R. equi genome products are of unknown function. (B) Functional categories of R. equi 103S secretome. The R. equi secretome comprises 736 CDSs, of which 44.5% encode proteins of unknown function, 20.3% correspond to transporters, 17.1% to lipoproteins, and 10.3% to extracellular enzymes possibly involved in nutrient breakdown and assimilation. (0.17 MB PDF) [file pgen.1001145.s006.pdf]

Fig S4

**A**

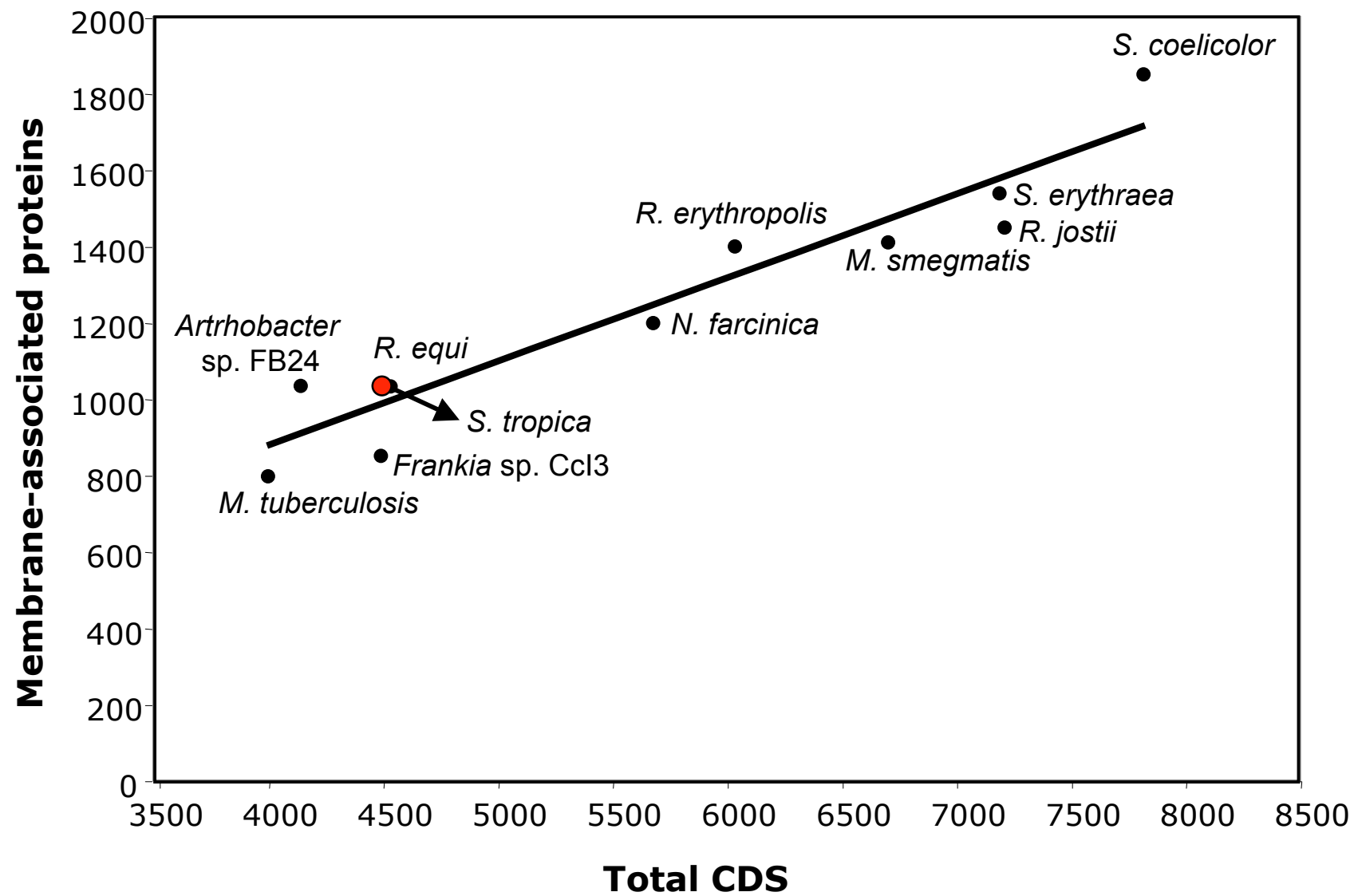

**B**

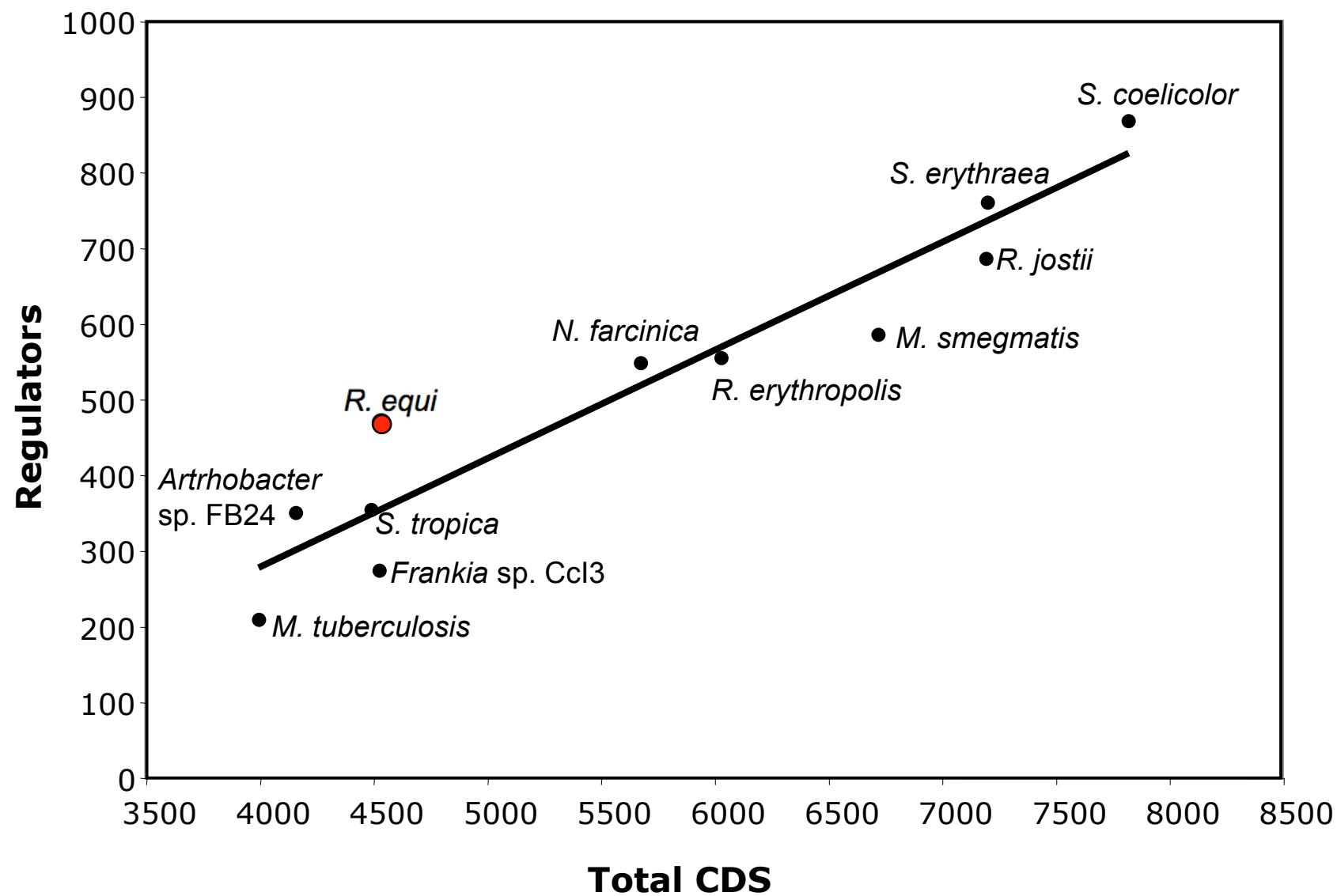

**C**

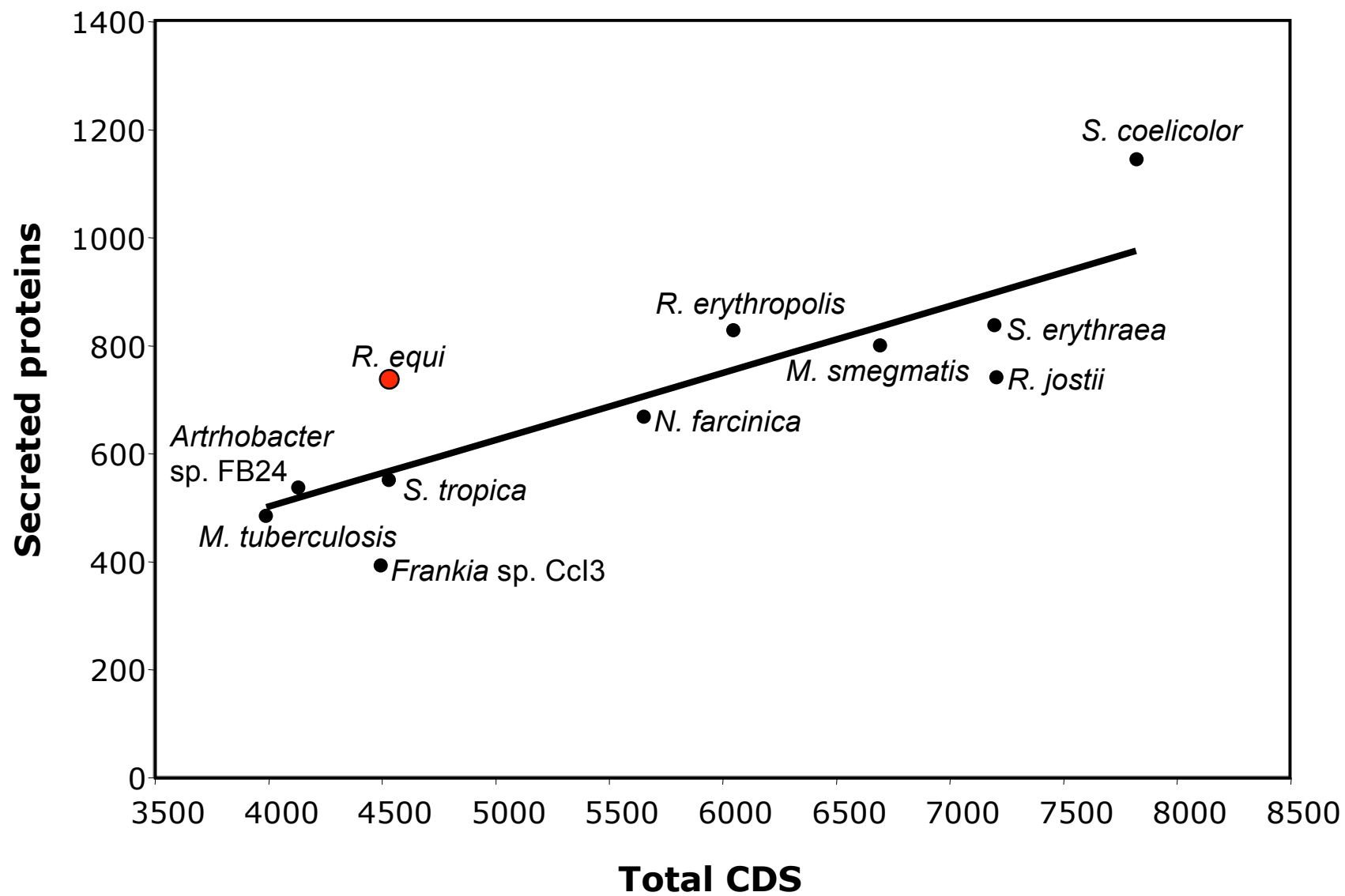

**D**

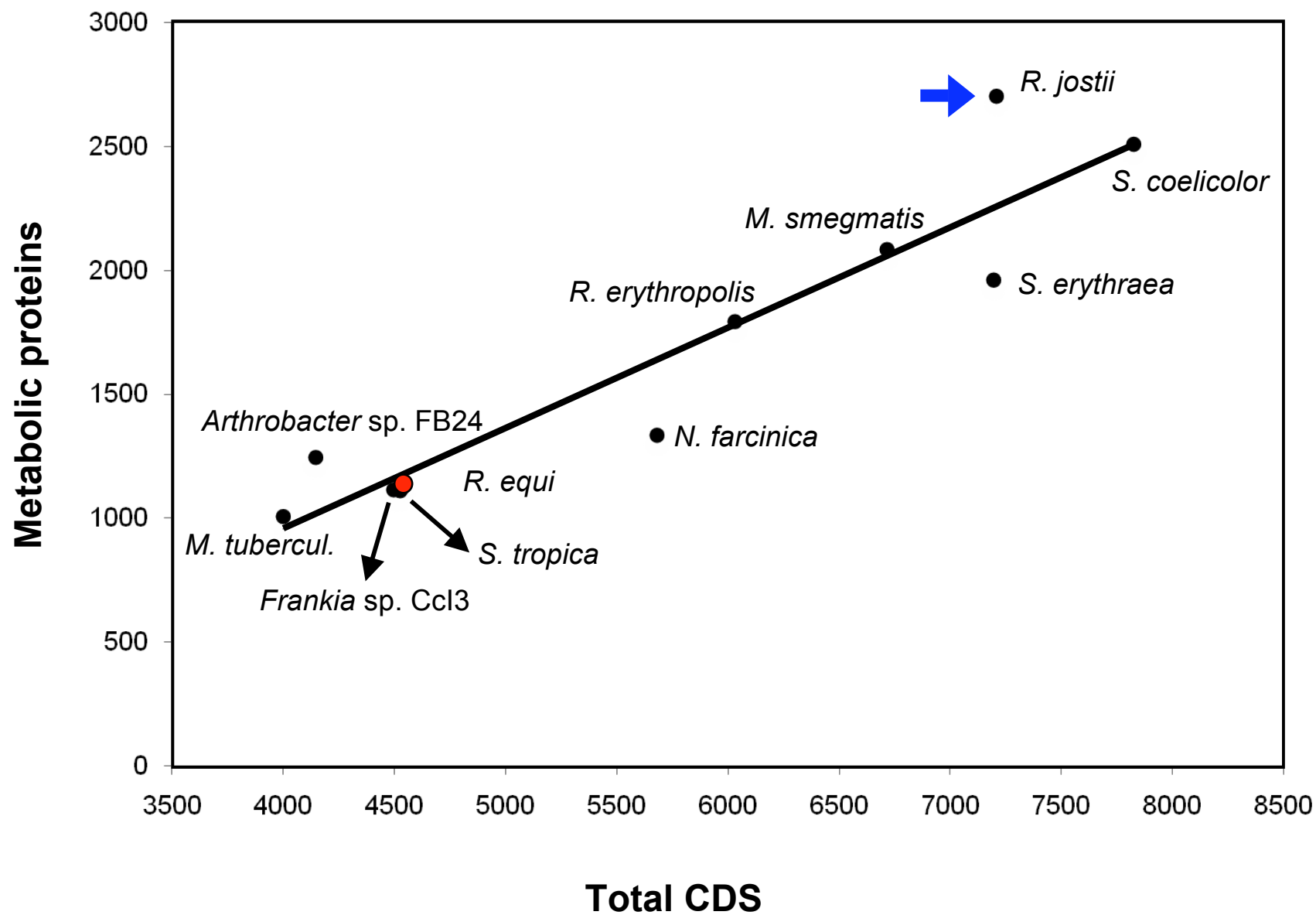

Supplement: Figure S4 — Scatter plots of selected functional categories vs genome size (≥4 Mb) of R. equi 103S and 10 other representative Actinobacteria. Data were inferred using the Comprehensive Microbial Resource (http://cmr.jcvi.org/) and the available genomes (Data Release 23.0). See Table S13 for accession nos. Membrane-associated and secreted proteins, as determined from TMHMM and SignalP outputs (see Materials and Methods). The number of regulators per genome has been calculated from keyword parsing of protein annotation. (A) Membrane-associated proteins. (B) Regulators. (C) Secreted proteins. (D) Metabolic proteins. (0.11 MB PDF) [file pgen.1001145.s007.pdf]

Fig S5

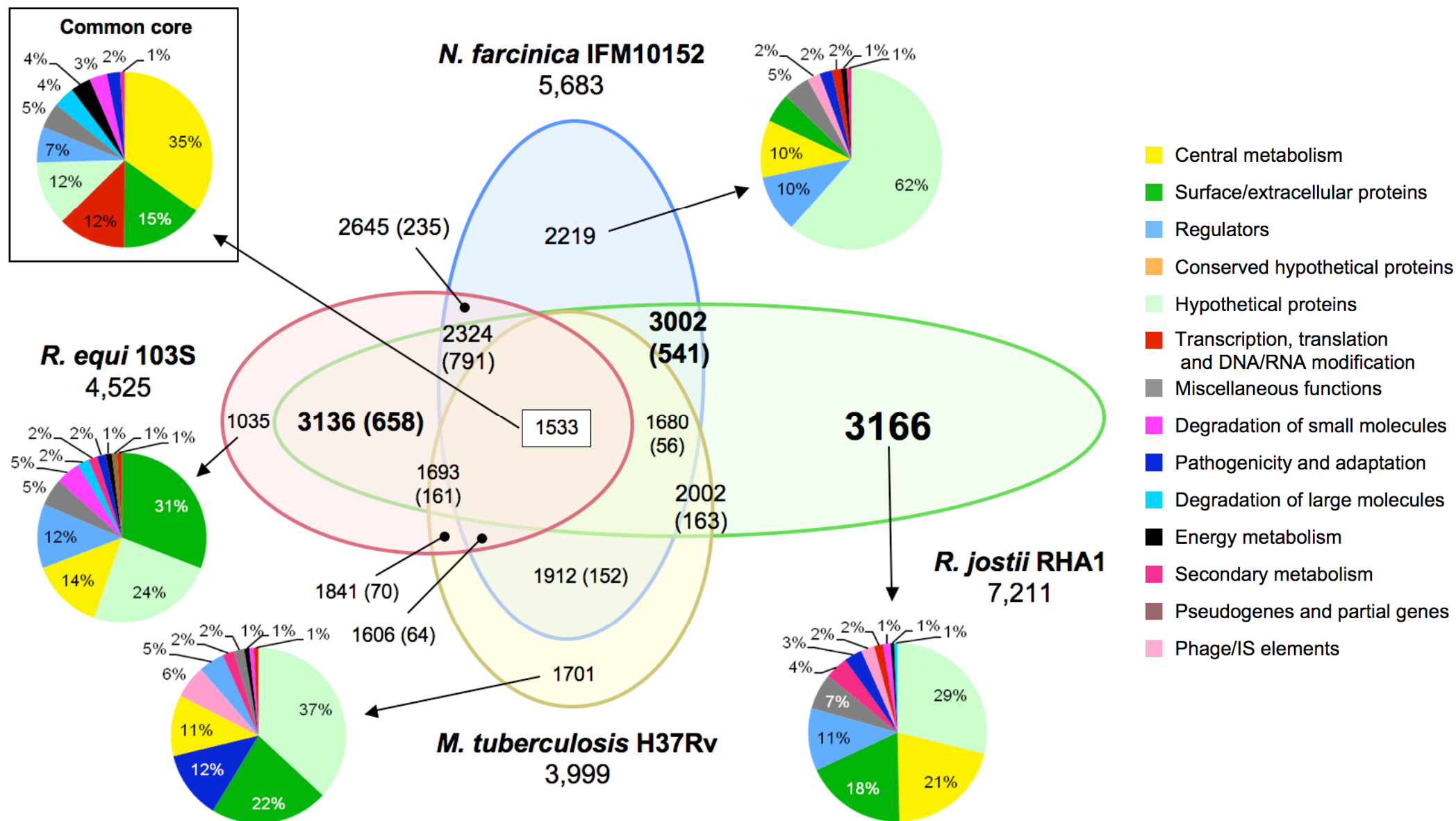

Supplement: Figure S5 — Species-specific gene complements of R. equi 103S, R. jostii RHA1, N. farcinica IFM10152, and M. tuberculosis H37Rv. The Venn diagram shows the number of chromosomal CDSs shared within a particular relationship (in brackets those unique to that relationship) as determined by ortholog comparisons (reciprocal FASTA best hits). Below the name of each species, the total number of genes in the genome is shown. The pie charts show the functional classification of the CDSs unique to each species and the shared common core. (0.35 MB PDF) [file pgen.1001145.s008.pdf]

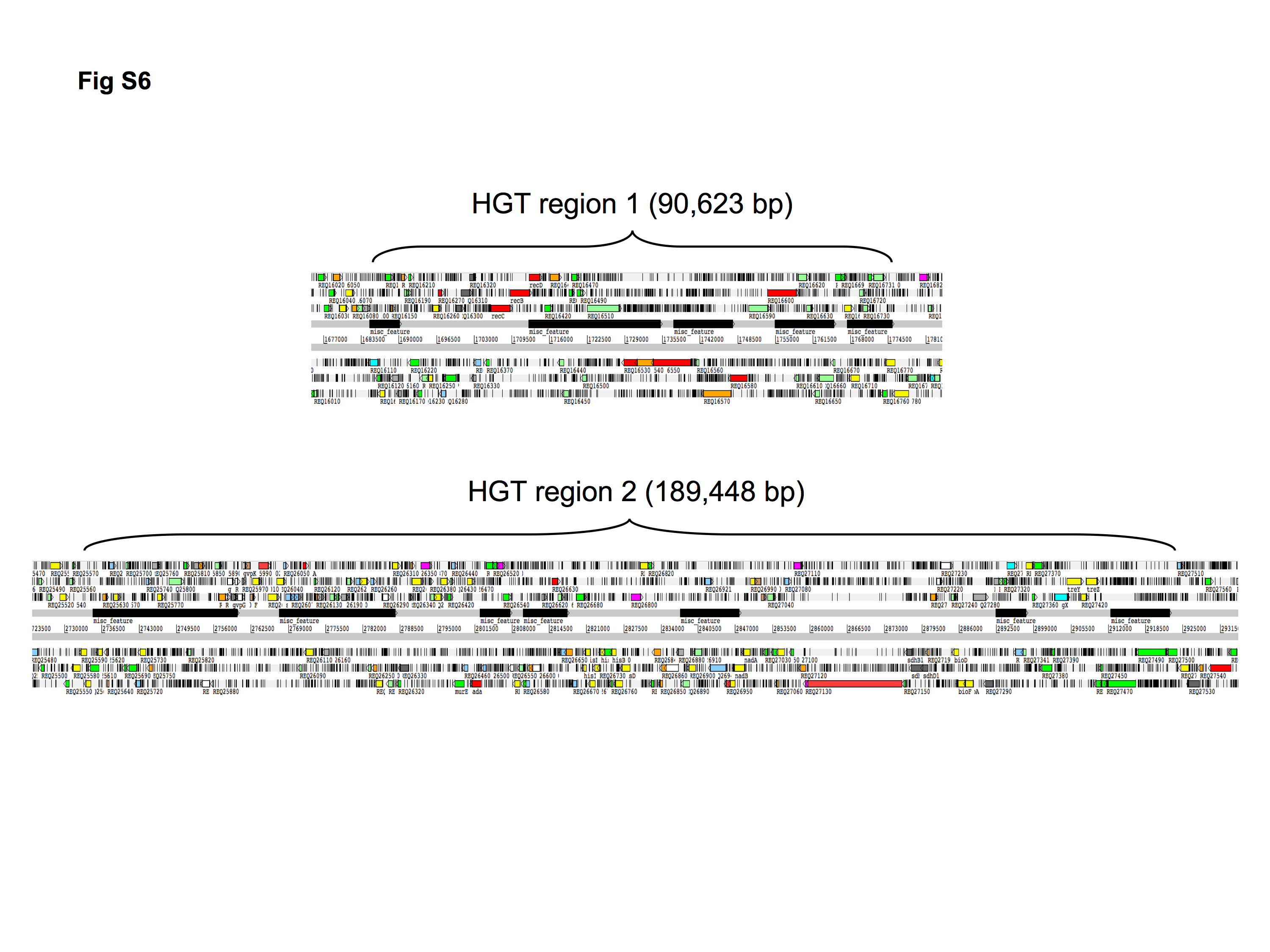

Supplement: Figure S6 — Genetic structure of the two large chromosomal HGT regions in R. equi 103S. The position of these regions on the chromosome is indicated in Figure S1. Functional categories of the genes are indicated in color code as in Figure S3. Alien Hunter [92] HGT hits are indicated as black bars in the center. HGT region 1 (positions 1,684,996-1,775,619, REQ16110-770) encompasses 68 CDSs and is rich in genes encoding nucleases, helicases and restriction enzymes. HGT region 2 (positions 2,734,493-2,848,474, REQ25610-26970) encompasses 132 CDSs with a diversity of functional categories but mostly involved in metabolism. It also includes three of the 14 pseudogenes found on the R. equi 103S chromosome. The mosaic structure of these regions and the diversity of source species, as indicated by reciprocal BLASTP best-hit analysis, suggest they are a composite of several independent HGT events rather than the result of a single “en block” acquisition. (1.14 MB PNG) [file pgen.1001145.s009.png]

**Fig S7**

**A**

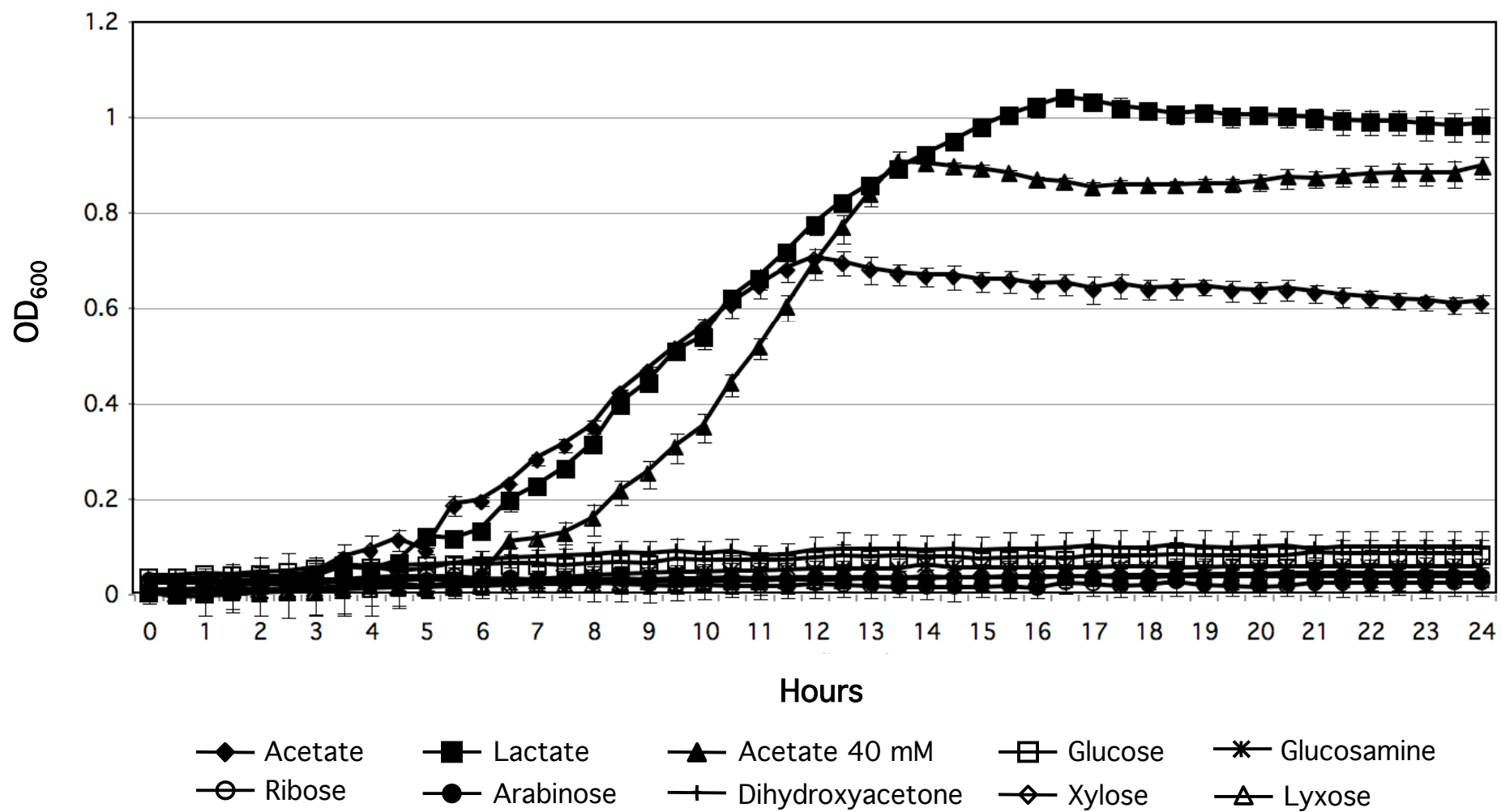

**B**

*R. erythropolis* PR4

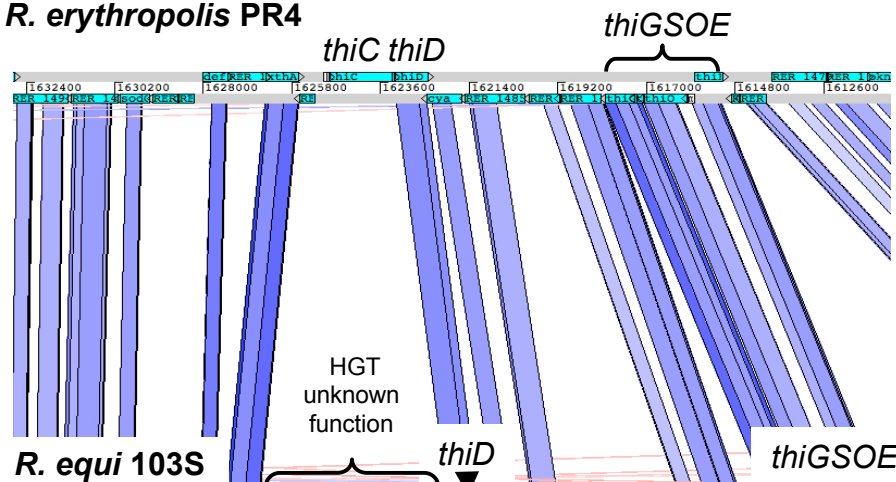

*R. equi* 103S

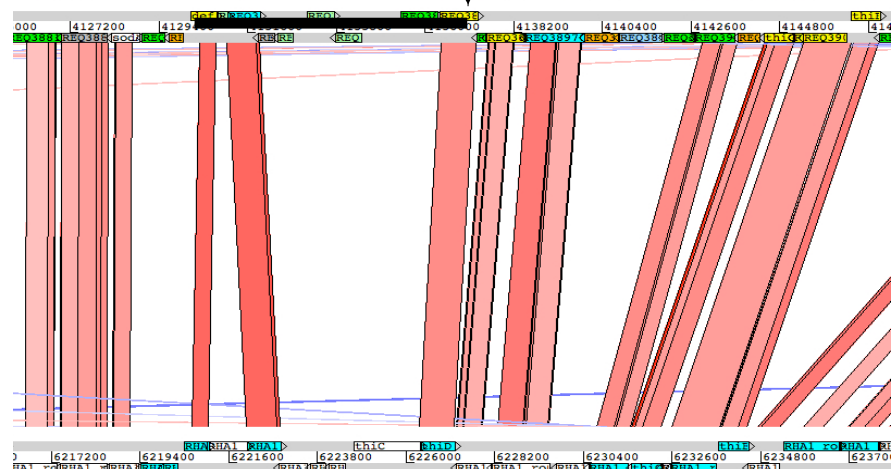

*R. jostii* RHA1

*thiC* *thiD* *thiGSOE*

**C**

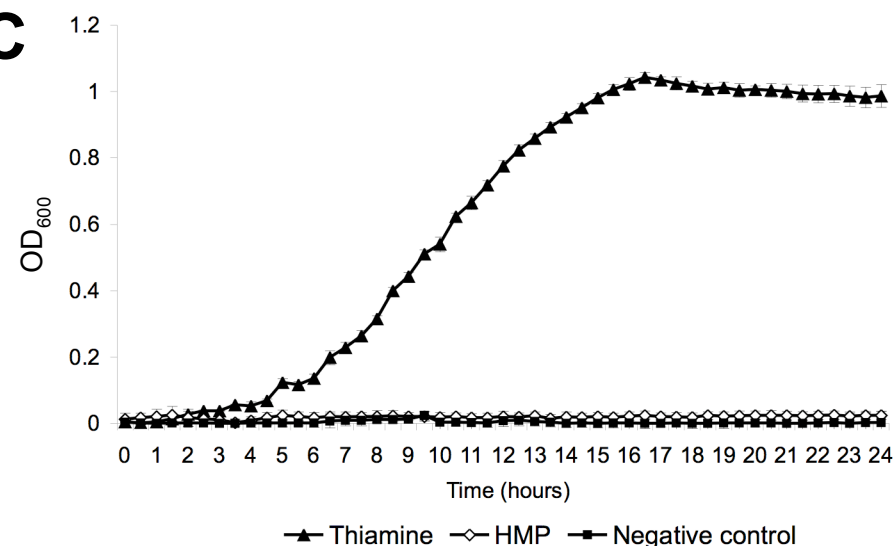

**D**

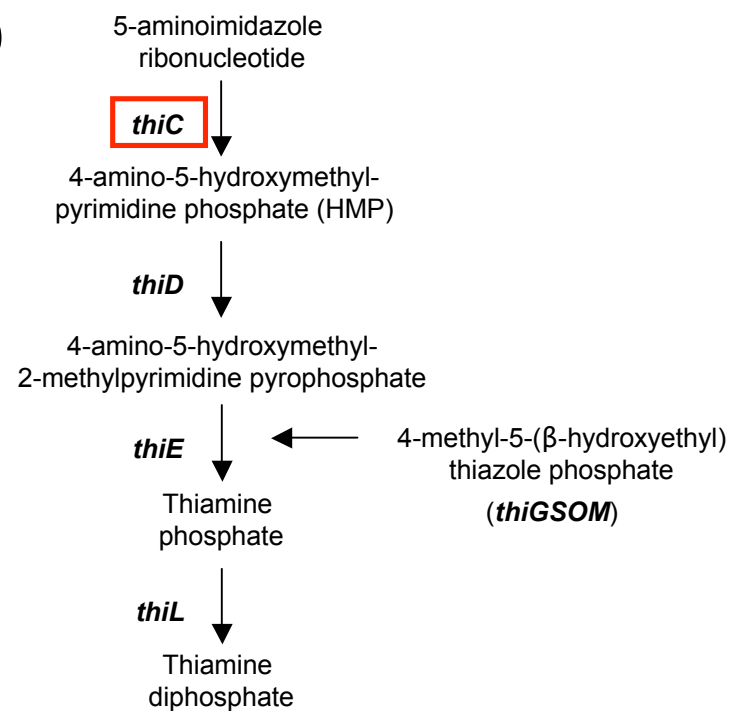

Supplement: Figure S7 — R. equi nutrition and metabolism. (A) Carbon source utilization. Growth assays of R. equi 103S in mineral medium (MM) [19] at 37°C. MM was supplemented (unless otherwise stated) with 20 mM of the indicated carbon sources and bacterial growth was monitored at OD600 every 30 min in a Fluostar Omega plate reader (BMG Labtech). Growth was detected only with lactate and acetate (mean of three experiments ±SD). Chemicals were purchased from Sigma. The nutritional and metabolic profile of R. equi (and its susceptibility to various chemicals and antibiotics) was initially investigated with Phenotype MicroArray (PMA) screens [15]. In the PMA plates PM1 and PM2 (carbon sources), certain substrates (e.g. glucose, arabinose, ribose, xylose, D-glucosamine, dihydroxyacetone and lyxose) sometimes give false positive results due to abiotic dye reduction (source: Michael Ziman, Biolog Inc). Experiments in MM confirmed that R. equi 103S does not utilize these substrates as sole carbon source. (B) ACT pairwise comparison of the thiamine biosynthesis gene clusters thiCD and thiGSOE in R. equi 103S and environmental rhodococci. In R. equi, the thiC gene has been replaced by an HGT region (black bar in the center) encoding proteins of unknown function. (C) Thiamine auxotrophy. Growth assay of R. equi 103S in 20 mM lactate MM medium. HMP, 4-amino-5-hydroxymethyl-pyrimidine phosphate (5% v/v of the crude preparation described in [94]). Negative control: no supplement. Most (∼80%) of the R. equi strains displayed thiamine auxotrophy. Experimental conditions as described in the legend to (A). (D) Diagram of the rhodococcal thiamine biosynthesis pathway. The thiCD genes are required for the production of 4-amino-5-hydroxymethyl-2-methylpyrimidine pyrophosphate; thiGSOM are involved in the generation of 4-methyl-5-(β-hydroxyethyl) thiazole phosphate, the second substrate required for the thiE-mediated synthesis of thiamine phosphate. Thiamine phosphate is ultimately phosphorylated by the produ [file pgen.1001145.s010.pdf]

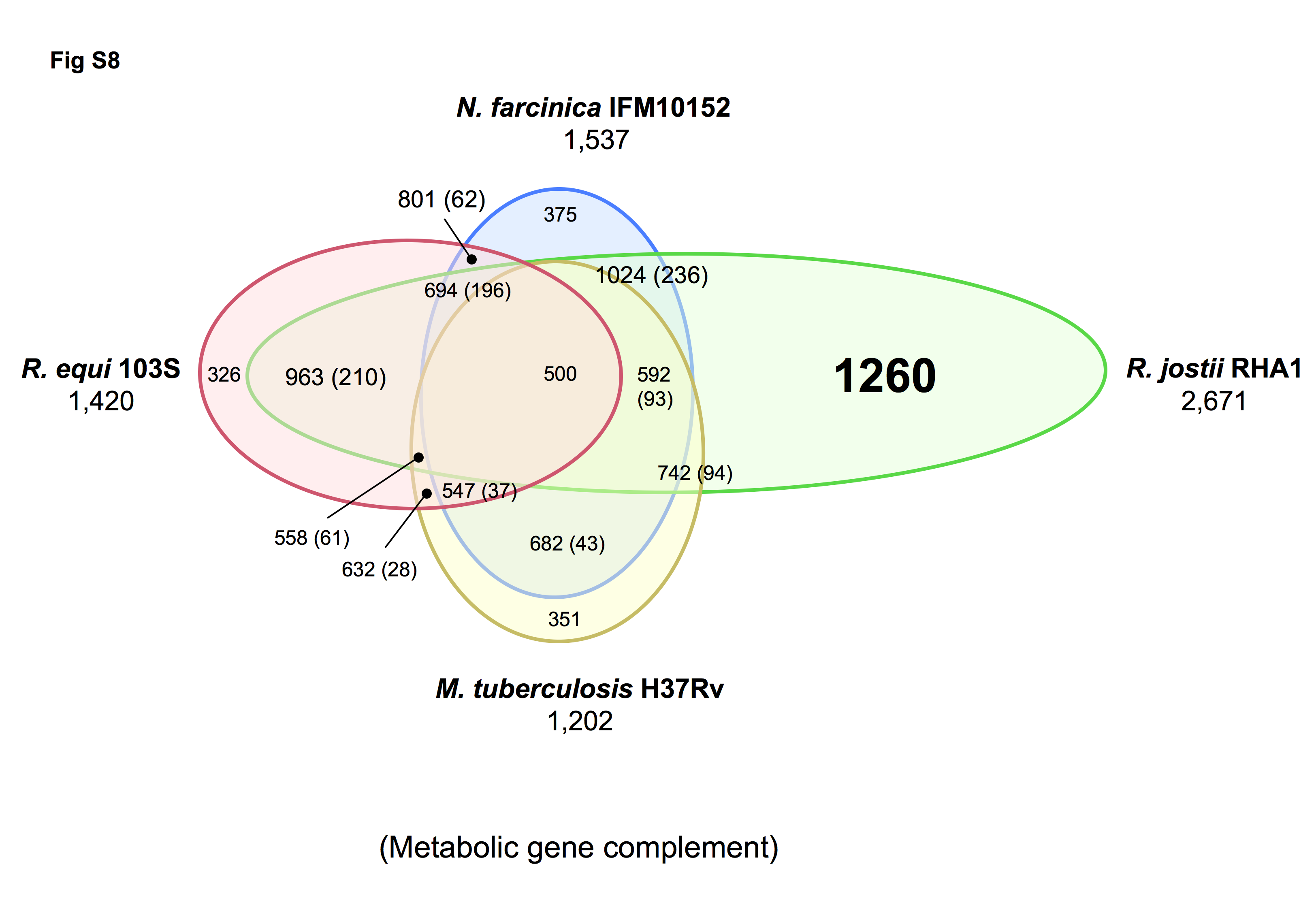

Supplement: Figure S8 — Species-specific metabolic gene complements of R. equi 103S, R. jostii RHA1, N. farcinica IFM10152, and M. tuberculosis H37Rv. Determined by ortholog comparison (reciprocal FASTA best hits). As the functional categories used for the annotation of the four genomes were not directly comparable, we first extracted the metabolism-related CDSs manually, on the basis of their predicted function. The Venn diagram shows the number of CDSs shared within a particular relationship (in brackets those unique to that relationship). Below the name of the species, the total number of metabolic genes present in the genome is shown. See Table S5 for paralogy analysis of the species-specific metabolic gene complements. (0.36 MB PNG) [file pgen.1001145.s011.png]

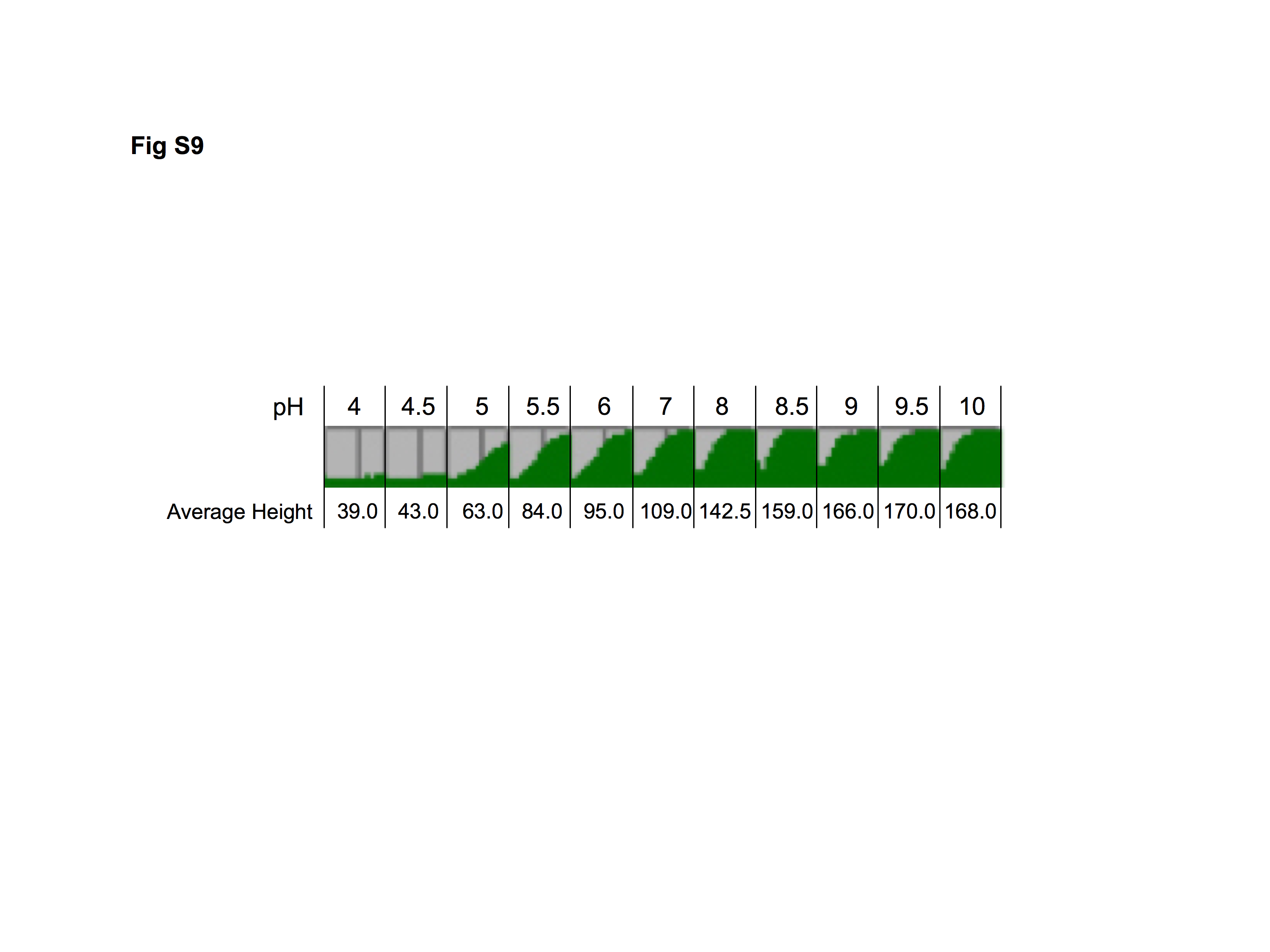

Supplement: Figure S9 — Optimal growth pH of R. equi 103S. Phenotype MicroArray [15] output of the relevant wells of plate PM10. Incubation was for 48 h at 37°C in an OmniLog instrument with readings taken every 15 minutes. Data were analyzed with OmniLog PM software. Consensus phenotypes for at least two replicas were determined based on the area difference under the kinetic curve of dye formation. Reported optimal pH values for other rhodococcal species: R. imtechensis 7.0 [95], R. koreensis 7.0–7.8 [96], R. kroppenstedtii 8.0 [97], R. kunmingensis 7.0–7.5 [98], R. kyotonensis 7.0 [99], R. percolatus 7.0–7.5 [100], R. pyridinivorans 7.5–8.5 [101], R. tukisamuensis 5.5–8.5 [102], R. yunnanensis 7.0–8.0 [103]. (0.23 MB PNG) [file pgen.1001145.s012.png]

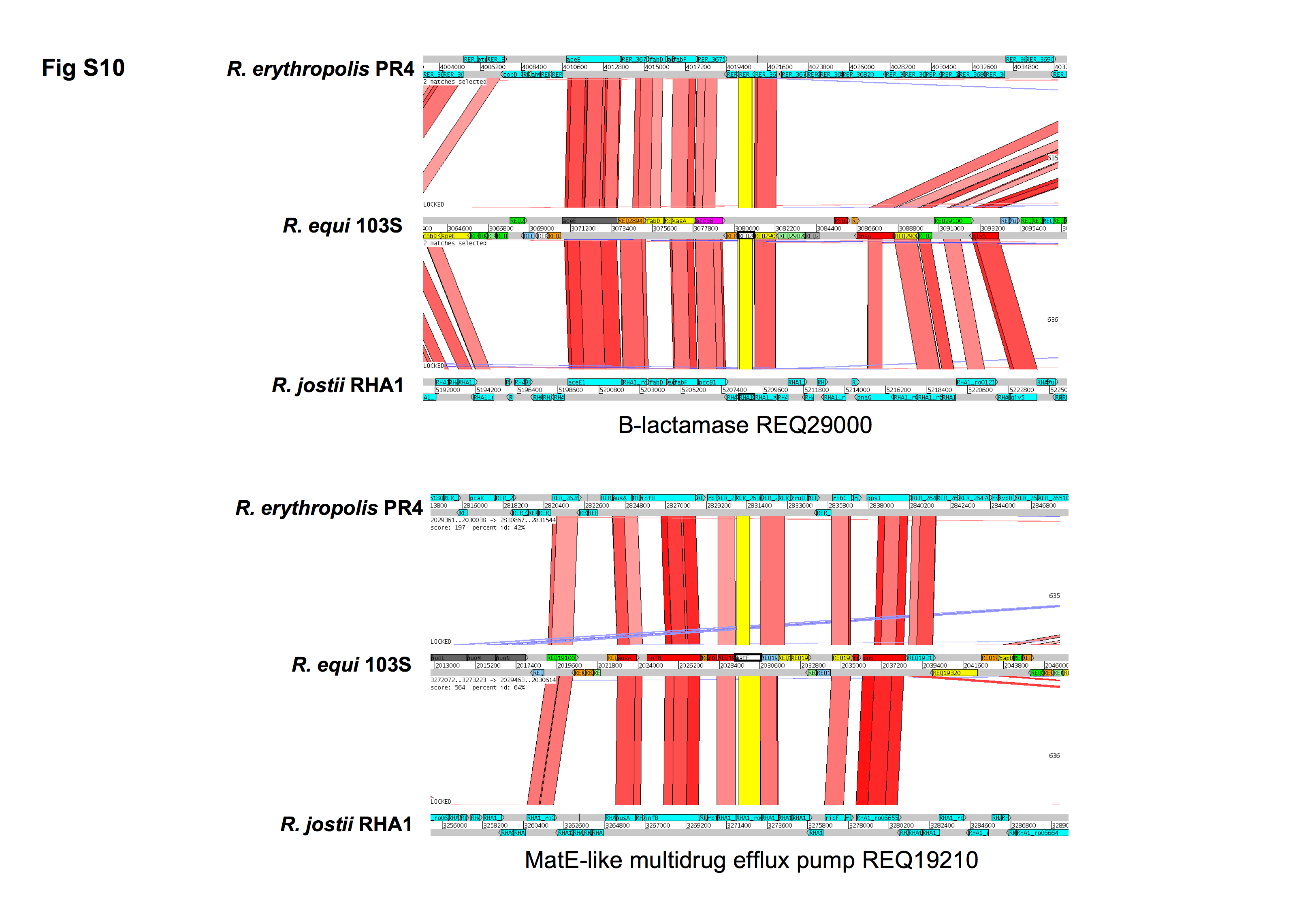

Supplement: Figure S10 — Examples of antibiotic resistance determinants located at the same chromosomal position in R. equi and two environmental Rhodococcus spp. Homologous resistance determinants indicated by yellow stripes in the ACT alignments. (0.49 MB PNG) [file pgen.1001145.s013.png]

Fig S11

**A**

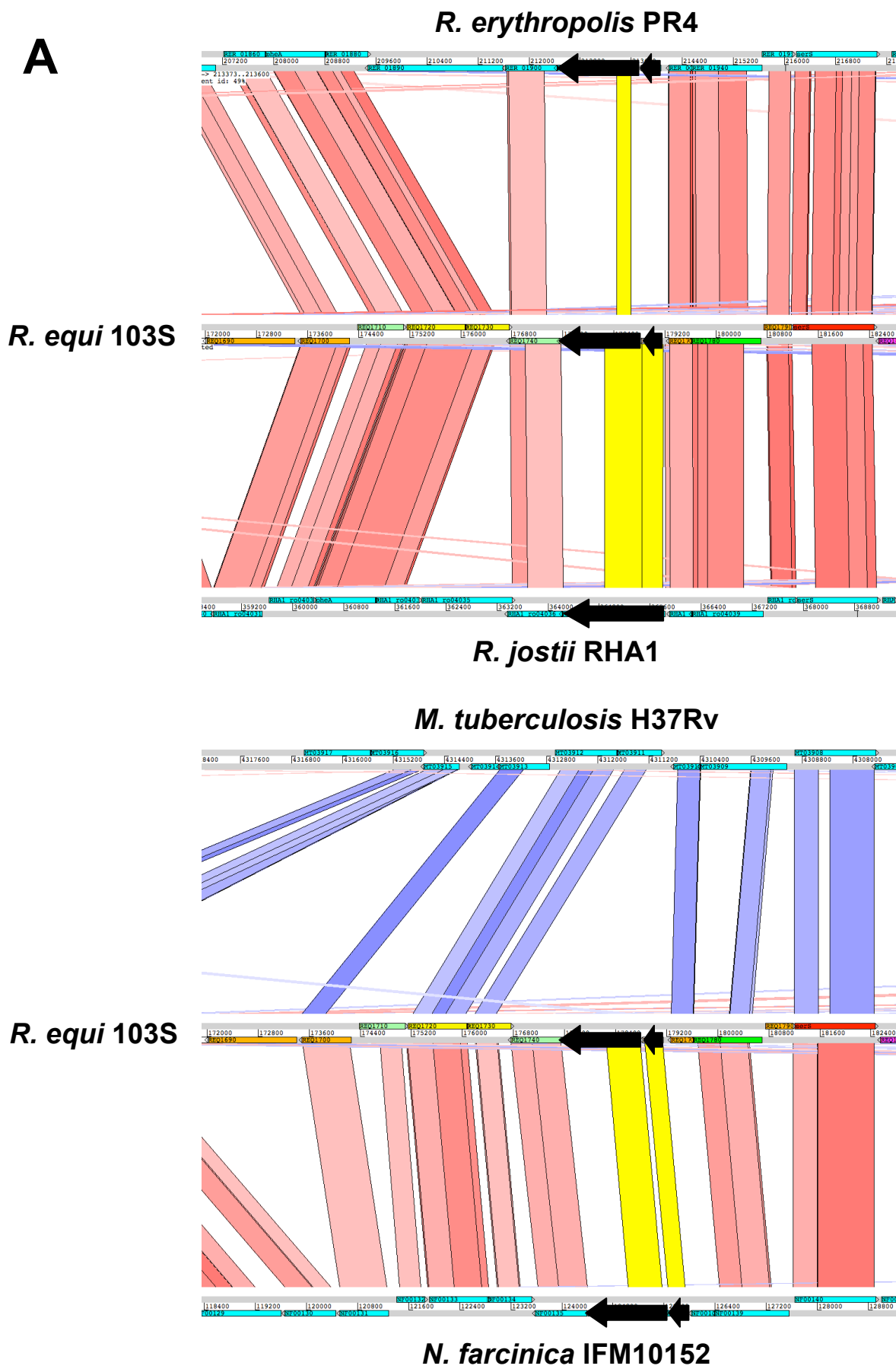

**B**

*R. erythropolis* PR4

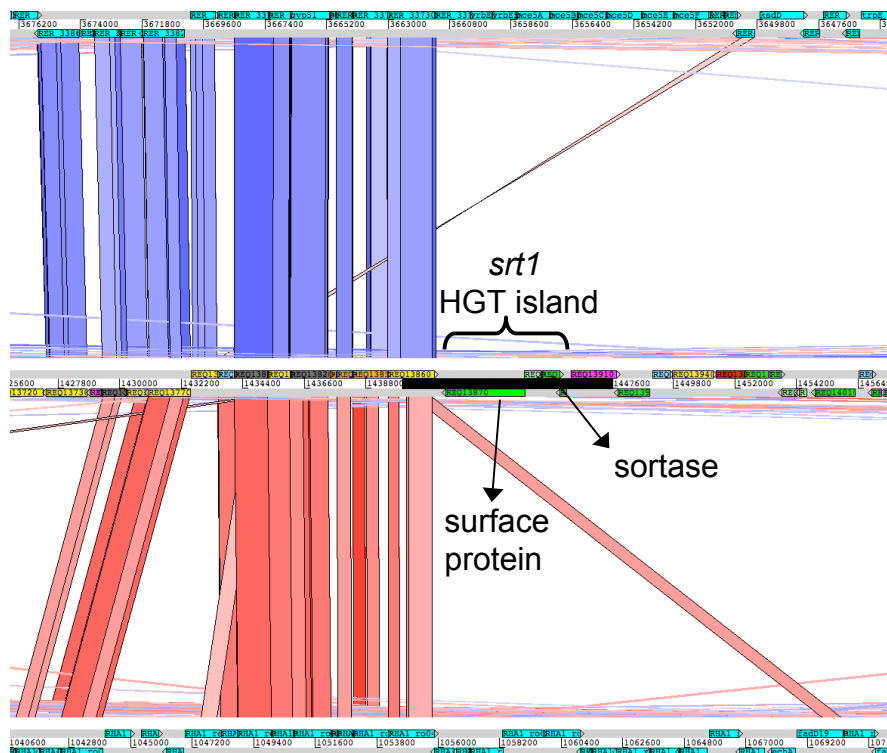

*R. equi* 103S

*R. jostii* RHA1

*R. erythropolis* PR4

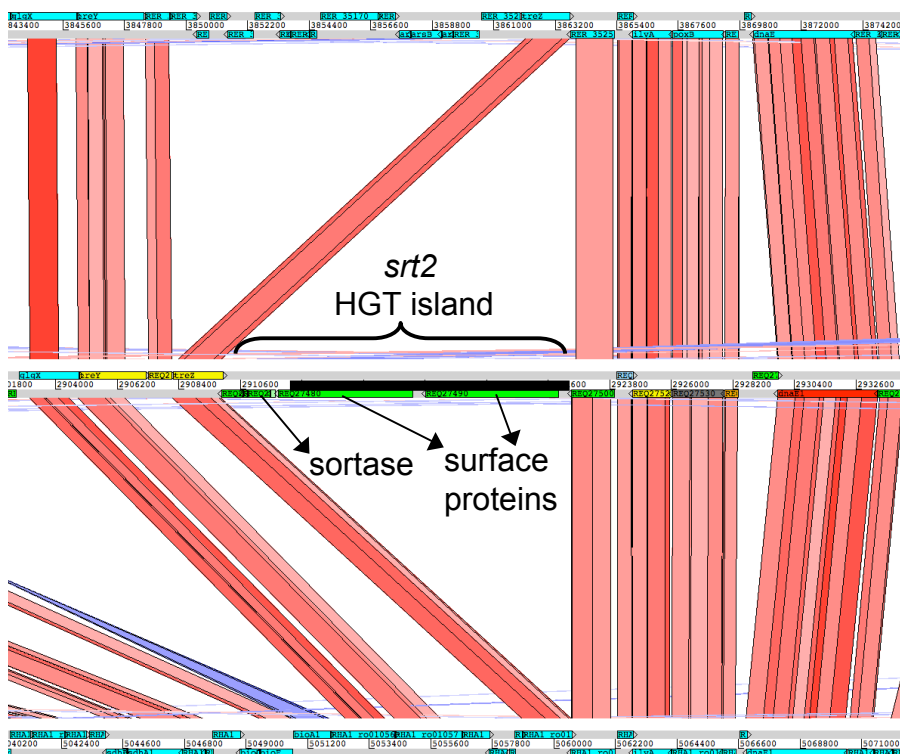

*R. equi* 103S

*R. jostii* RHA1

Supplement: Figure S11 — Virulence-related loci of R. equi 103S. (A) PE/PPE locus and corresponding chromosomal regions in R. jostii RHA1, R. erythropolis PR4, N. farcinica IFM10152 and M. tuberculosis H37Rv. Arrows in ACT alignments indicate PE and PPE genes. The PE gene is of the “short” subclass (only a conserved N-terminal PE module of 99 to 102 residues); the PPE gene is of the “unique C-terminal domain” subclass [104]. The R. equi PE/PPE locus is inserted at the same chromosomal position in the nonpathogenic Rhodococcus spp. and in N. farcinica; no PE/PPE genes are present at the corresponding chromosomal region of Mtb, other mycobacteria and corynebacteria, indicating this PE/PPE locus is specific to the Nocardiaceae within the Corynebacterinae. The PE/PPE genes are fused in R. jostii RHA1. (B) Sortase HGT islands srt1 and srt2 of R. equi 103S. ACT comparisons of srt1 (above) and srt2 (below) and corresponding regions of R. jostii RHA1 and R. erythropolis PR4. Alien Hunter [92] outputs indicated as black bars in the center. srt1 is unique to R. equi among the sequenced Rhodococcus spp., including R. opacus B4) (not shown). The srt2 island is conserved in R. erythropolis but at a different chromosomal location and encoding only one of the two putative sortase substrates (surface protein RER_38400, which like its R. equi homolog REQ27480 contains an LPVTG sorting motif). Apart from a serine peptidase encoded by the esx locus (REQ35490), no proteins with the typical hallmarks of sortase substrates, i.e. a C-terminal membrane-spanning region preceded by a sortase recognition motif LPXTG, or a variant thereof) [105], are encoded outside the two srt islands. (0.75 MB PDF) [file pgen.1001145.s014.pdf]

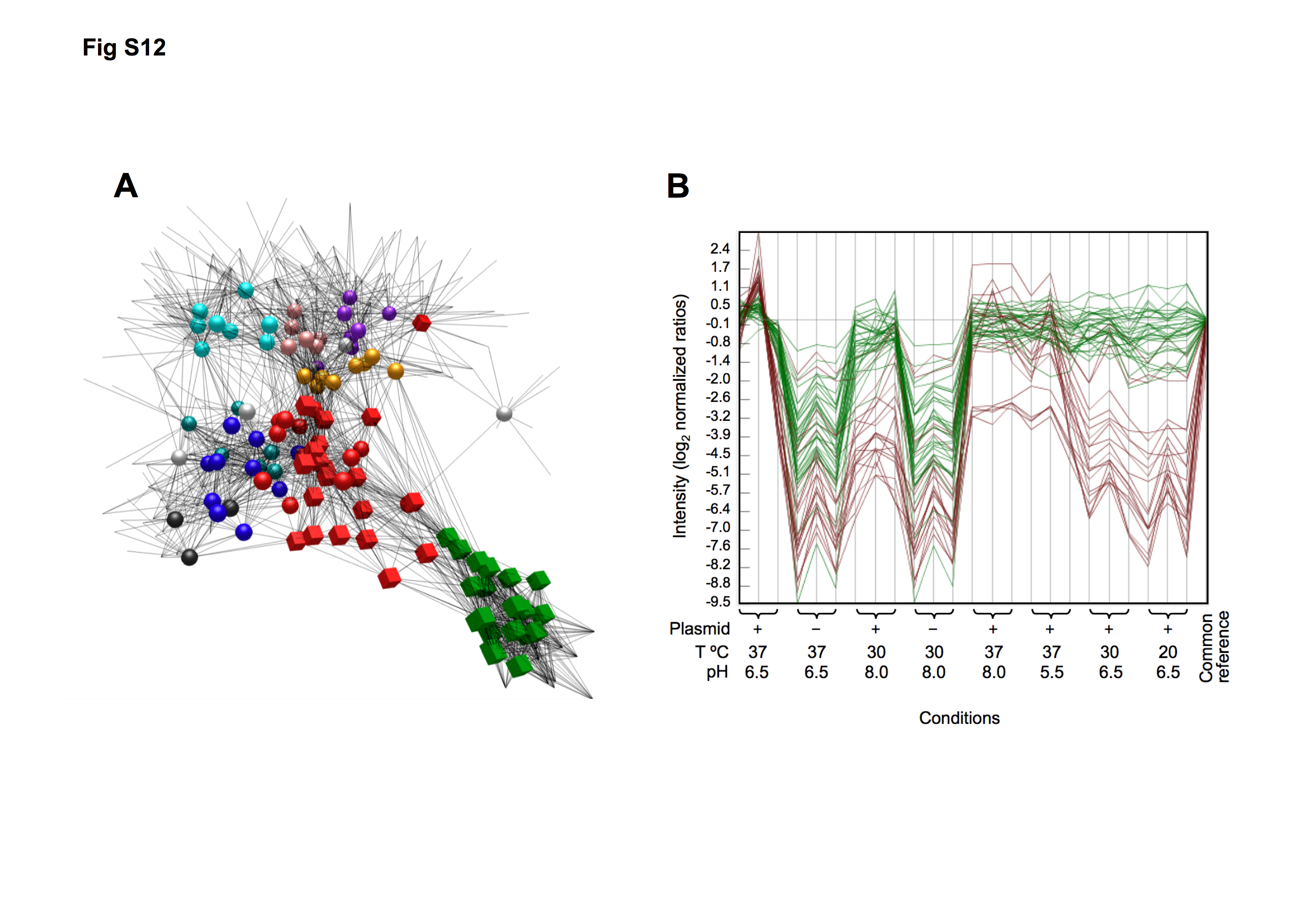

Supplement: Figure S12 — Network analysis of R. equi microarray expression data. (A) Detail of the network graph of Figure 5A showing the web of functional linkages (edges) between the vap PAI-coregulated cluster (red nodes) and direct neighbor clusters (green nodes, plasmid backbone cluster; other clusters represented in different colors; individual directly connected nodes are in gray regardless of whether they belong to a larger cluster; chromosomal nodes are represented as spheres, plasmid nodes as cubes). All other nodes have been removed. Predominant functional classes among neighbor clusters (n = 129 nodes): Central and energy metabolism 27.1%, Membrane-associated/surface proteins/transporters 23.3%, Hypothetical proteins 18.6%, Regulators 9.3%, Degradation of small molecules 7.75%. Metabolism-related products encoded by direct neighbor nodes include enzymes of the shikimate pathway/biosynthesis of aromatic amino acids (prephenate dehydrogenase REQ02960, prephenate dehydratase REQ01720); porphyrin metabolism (magnesium chelatase REQ18110) and cobalamin biosynthesis (uroporphyrinogen-III C-methyltransferase REQ02960, CobB homolog REQ28830); synthesis of cysteine, activated sulfate (cysB, D, G, K/M, Q and N/C homologs); and mycothiol (mycothiol ligase MshC REQ22990), urease (UreA, C, D, F, and G homologs), and nitrite reductase NirB1 (REQ32930). (B) Representative expression profiles of the plasmid gene-containing clusters identified with r = 0.85 Pearson correlation threshold (see Table S11). Maroon lines, vap PAI-coregulated cluster (red and yellow nodes in Figure 5A); green lines, plasmid backbone cluster (green nodes in Figure 5A). The individual profiles of three biological replicates per test condition are plotted. Note that the vap PAI-coexpressed cluster, which includes chromosomal genes, is activated by both plasmid and temperature (37°C) whereas the plasmid backbone cluster is expressed constitutively in the same conditions. Common reference: average signal of 103S at 37°C pH [file pgen.1001145.s015.png]
