# Supplementary material for: The Genome of a Pathogenic Rhodococcus: Cooptive Virulence Underpinned by Key Gene Acquisitions
Source: PLoS Genet. 2010 Sep 30;6(9):e1001145. doi: 10.1371/journal.pgen.1001145 (PMC2947987; doi:10.1371/journal.pgen.1001145)
Supplement: Table S1 — Statistics of horizontal gene acquisition (HGT) in actinobacterial chromosomes. HGT DNA was identified with the Alien Hunter program (http://www.sanger.ac.uk/Software/analysis/), which identifies horizontally acquired DNA by reliably capturing local compositional biases based on a variable-order motif distributions method [92]. The thick gray line delimits the genomes with chromosomes of less than and more than 4 Mb in size. Accession nos. of the genomes used are shown in Table S13. (0.09 MB PDF) [file pgen.1001145.s016.pdf]

Table S1

Species	Total genes	HGT genes	HGT %	HGT events	Genes / HGT event	HGT frequency (1 every *n* genes)
Tropheryma whipplei	784	40	5.10	8	5.00	98.00
Bifidobacterium longum	1727	90	5.21	9	10.00	191.89
Leifsonia xyli	2030	402	19.80	47	8.55	43.19
Propionibacterium acnes	2297	138	6.01	12	11.50	191.42
Corynebacterium diphtheriae	2320	811	34.96	65	12.48	35.69
Clavibacter michiganensis	3008	246	8.18	22	11.18	136.73
Corynebacterium glutamicum	3058	512	16.74	39	13.13	78.41
Thermobifida fusca	3110	645	20.74	85	7.59	36.59
Rubrobacter xylanophilus	3140	265	8.44	19	13.95	165.26
Mycobacterium tuberculosis	3999	656	16.40	68	9.65	58.81
Arthrobacter sp. FB24	4146	542	13.07	53	10.23	78.23
Frankia sp. CcI3	4499	1572	34.94	158	9.95	28.47
Rhodococcus equi	4525	430	9.50	52	8.27	87.02
Salinispora tropica	4536	1236	27.25	121	10.21	37.49
Nocardia farcinica	5683	979	17.23	77	12.71	73.81
Rhodococcus erythropolis	6034	891	14.77	90	9.90	67.04
Mycobacterium smegmatis	6716	1650	24.57	138	11.96	48.67
Saccharopolyspora erythraea	7198	911	12.66	89	10.24	80.88
Rhodococcus jostii	7211	1407	19.51	133	10.58	54.22
Streptomyces coelicolor	7826	1643	20.99	173	9.50	45.24
					Mean ≥4 Mb	59.99±19.03
