# Supplementary material for: The Genome of a Pathogenic Rhodococcus: Cooptive Virulence Underpinned by Key Gene Acquisitions
Source: PLoS Genet. 2010 Sep 30;6(9):e1001145. doi: 10.1371/journal.pgen.1001145 (PMC2947987; doi:10.1371/journal.pgen.1001145)
Supplement: Table S2 — Chromosomal gene duplication and paralogous families in R. equi 103S and 19 other representative Actinobacteria. Paralogous families were identified by clustering of proteomes with BLASTClust (see Table S12). (0.07 MB PDF) [file pgen.1001145.s017.pdf]

Table S2

Species	Total no. genes	Duplicated genes	% duplicated genes	No. paralogous families	Maximum no. genes in a family	Non-duplicated genes
Streptomyces coelicolor	7826	2534	32.38	629	78	5292
Rhodococcus josti	7211	2426	33.64	623	101	4785
Saccharopolyspora erythraea	7198	2626	36.48	669	76	4572
Mycobacterium smegmatis	6716	1988	29.60	490	128	4728
Rhodococcus erythropolis	6034	2006	33.24	531	92	4028
Nocardia farcinica	5683	1695	29.83	453	82	3988
Salinispora tropica	4536	936	20.63	294	48	3600
Rhodococcus equi	4525	1452	32.09	386	89	3073
Frankia sp. CcI3	4499	603	13.40	226	19	3896
Arthrobacter sp. FB24	4146	1081	26.07	329	39	3065
Mycobacterium tuberculosis	3999	875	21.88	259	84	3124
Rubrobacter xylanophilus	3140	1031	32.83	321	42	2109
Thermobifida fusca	3110	453	14.57	143	26	2657
Corynebacterium glutamicum	3058	402	13.15	148	41	2656
Clavibacter michiganensis	3008	707	23.50	224	41	2301
Corynebacterium diphtheriae	2320	132	5.69	44	24	2188
Propionibacterium acnes	2297	270	11.75	95	35	2027
Leifsonia xyli	2030	204	10.05	73	19	1826
Bifidobacterium longum	1727	206	11.93	73	16	1521
Tropheryma whipplei	784	42	5.36	17	7	742
