# Supplementary material for: The Genome of a Pathogenic Rhodococcus: Cooptive Virulence Underpinned by Key Gene Acquisitions
Source: PLoS Genet. 2010 Sep 30;6(9):e1001145. doi: 10.1371/journal.pgen.1001145 (PMC2947987; doi:10.1371/journal.pgen.1001145)
Supplement: Table S3 — DNA mobility genes in R. equi 103S and environmental Rhodococcus spp genomes. Identified by keyword parsing of protein annotation; in brackets, genes associated with HGT regions. Plasmids from R. erythropolis PR4 published in [106]. (0.09 MB PDF) [file pgen.1001145.s018.pdf]

Table S3

	Replicon	Size (Kb)	Topology [b]	Transposases	Resolvases	Recombinases	Excisionases	Integrases	Other phage genes	Total
***R. equi* 103S**	Chromosome	5,043.2	C	1 (1)	0 (0)	0 (0)	0 (0)	4 (2)	0 (0)	5 (3)
	pVAPA1037	80.6	C	0 (0)	2 (2)	0 (0)	1 (0)	0 (0)	0 (0)	3 (2)
***R. jostii* RHA1**	Chromosome	7,804.8	L	82 (50)	7 (7)	4 (4)	4 (3)	1 (1)	2 (1)	100 (66)
	pRHL1	1,123.1	L	58 (32)	1 (0)	0 (0)	1 (0)	10 (3)	0 (0)	70 (35)
	pRHL2	442.5	L	40 (12)	0 (0)	0 (0)	0 (0)	5 (2)	0 (0)	45 (14)
	pRHL3	332.4	L	17 (8)	1 (0)	1 (0)	2 (2)	2 (0)	0 (0)	23 (10)
R. erythropolis	Chromosome	6,516.3	C	8 (8)	2 (1)	5 (5)	1 (1)	5 (5)	1 (1)	22 (21)
	pREL1	271.6	L	57 (23)	0 (0)	1 (0)	0 (0)	1 (0)	0 (0)	59 (23)
	pREC1	104.0	C	1 (1)	0 (0)	4 (2)	0 (0)	0 (0)	0 (0)	5 (2)
	pREC2[a]	3.6	C	0 (0)	0 (0)	0 (0)	0 (0)	0 (0)	0 (0)	0 (0)
***R. opacus* B4**	Chromosome	7,248.6	L	40 (0)	0 (0)	6 (0)	0 (0)	3 (0)	1 (1)	50 (1)
	pROB01	558.2	L	69 (21)	0 (0)	4 (4)	0 (0)	4 (0)	0 (0)	77 (25)
	pROB02	245.0	L	22 (4)	0 (0)	0 (0)	0 (0)	7 (2)	0 (0)	29 (6)
	pKNR	111.2	C	2 (1)	0 (0)	2 (1)	0 (0)	1 (0)	0 (0)	5 (2)
	pKNR01 [a]	4.4	C	0 (0)	0 (0)	0 (0)	0 (0)	0 (0)	0 (0)	0 (0)
	pKNR02 [a]	2.7	C	0 (0)	0 (0)	0 (0)	0 (0)	0 (0)	0 (0)	0 (0)

[a] Small-size replicons (< 4.4 Kb, less than 6 genes) in which DNA compositional biases are not reliably identified.
[b] C = circular, L = linear.
