# Supplementary material for: The Genome of a Pathogenic Rhodococcus: Cooptive Virulence Underpinned by Key Gene Acquisitions
Source: PLoS Genet. 2010 Sep 30;6(9):e1001145. doi: 10.1371/journal.pgen.1001145 (PMC2947987; doi:10.1371/journal.pgen.1001145)
Supplement: Table S4 — Phosphoenolpyruvate-sugar phosphotransferase system (PTS) components in a selection of actinobacterial genomes. Identified using motif search in Pfam database (Pfam motif identifiers indicated in footnotes). (0.10 MB PDF) [file pgen.1001145.s019.pdf]

Table S4

	EI [a]	HPr [b]	EIIA1 [c]	EIIA2 [d]	EIIB [e]	EIIC [f]	IIA [g]	IIB [h]	FruIIA [i]	FruIIB [j]	PRD [k]	GalacIIC [l]	SorbII [m]
Arthrobacter sp. FB24	X	X	X		X	X		X	X	X	X		
Bifidobacterium longum	X	X	X		X	X					X		
Clavibacter michiganensis	X	X				X		X	X	X	X		
Corynebacterium diphteriae	X	X	X	X	X	X		X	X		X		
Corynebacterium glutamicum	X	X	X	X	X	X		X		X	X		
Frankia sp. CcI3	X	X				X		X					
Leifsonia xyli	X	X				X		X	X	X	X		
Mycobacterium smegmatis	X	X	X	X	X	X			X	X			
Mycobacterium tuberculosis													
Nocardia farcinica	X	X		X		X				X			
Propionibacterium acnes	X	X	X		X	X	X	X	X	X	X	X	X
Rhodococcus equi													
Rhodococcus josti RHA1	X	X	X	X	X	X				X			
Saccharopolyspora erythraea	X	X	X		X	X			X	X			
Salinispora arenicola	X	X				X		X					
Streptomyces coelicolor	X	X	X	X	X	X		X	X	X			
Thermobifida fusca	X	X	X										
Tropheryma whipplei													

[a] EI component (PF05524)
[b] HPr phosphocarrier protein (PF00381)
[c] EIIA 1 sugar-specific permease complex component (PF00358)
[d] EIIA 2 sugar-specific permease complex component (PF00359)
[e] EIIB sugar-specific permease complex component (PF00367)
[f] EIIC sugar translocation channel component (PF02378)
[g] Lactose/Cellobiose specific IIA subunit (PF02255)
[h] Lactose/Cellobiose specific IIB subunit (PF02302)
[i] Fructose IIA component (PF03610)
[j] Fructose IIB component (PF03830)
[k] PRD domain (for PTS Regulation Domain) (PF00874)
[l] Galactitol-specific IIC component (PF03611)
[m] Sorbitol EII N-terminus (PF03612)
