# Supplementary material for: The Genome of a Pathogenic Rhodococcus: Cooptive Virulence Underpinned by Key Gene Acquisitions
Source: PLoS Genet. 2010 Sep 30;6(9):e1001145. doi: 10.1371/journal.pgen.1001145 (PMC2947987; doi:10.1371/journal.pgen.1001145)
Supplement: Table S5 — Ranking of the ten most populated paralogous metabolic gene families of R. equi 103S, R. jostii RHA1, N. farcinica IFM10152, and M. tuberculosis H37Rv. Determined by BLASTCLUST analysis. In brackets, number of paralogs within the family. (0.09 MB PDF) [file pgen.1001145.s020.pdf]

Table S5

	R. equi 103S	*R. jostii* RHA1	*M. tuberculosis* H37Rv	*N. farcinica* IFM10152	
	**Species-specific metab. genes**	**Species-specific metab. genes**	**Species-specific metab. genes**	**Species-specific metab. genes**	
**1**	Short chain dehydrogenases (25)	Short chain dehydrogenases (50)	Acyl-CoA ligase/synthetase (9)	Short chain dehydrogenases (14)	
**2**	Acyl-CoA ligase/synthetase (7)	Aldehyde dehydrogenases (36)	Secreted cutinases (6)	P450 monooxygenases (11)	
**3**	Aldehyde dehydrogenases (4)	Acyl-CoA dehydrogenases (27)	Aldehyde dehydrogenases (4)	Acyl-CoA ligase/synthetase (9)	
**4**	Monooxygenases (4)	Zinc-alcohol dehydrogenases (24)	P450 monooxygenases (3)	Esterases (7)	
**5**	Enoyl-CoA hydrat./isom.(4)	Acyl-CoA ligase/synthetase (23)	Esterases (3)	Aldehyde dehydrogenases (6)	
**6**	Amidases (3)	Enoyl-CoA hydratase/isomerases (18)	Acetyl hydrolases (3)	Oxygenases (5)	
**7**	Secreted lipases (3)	Formyl-CoA transferases (13)	Adenylate cyclase (2)	Monooxygenases (4)	
**8**	Oxidoreductases (3)	Acetyl-CoA C-acyltransferase (10)	Glycosyltransferases (2)	ATP/GTP binding proteins (4)	
**9**	Zinc-alcohol dehydrogenases (3)	Phosphoglycerate dehydrogenases (9)	Aminotransferases (2)	Acyl-CoA thiolases (3)	
**10**	Dehydrogenases (3)	Nitrilotriacetate monooxygenases (9)	Methyltransferases (2)	Glycosyltransferases (3)	
	No. paralogous families = 38	No. paralogous families = 163	No. paralogous families = 20	No. paralogous families = 38	
	**Total metabolic genes**	**Total metabolic genes**	**Total metabolic genes**	**Total metabolic genes**	**Core genome metabolic genes**
**1**	Short chain dehydrogenases (95)	Short chain dehydrogenases (127)	Short chain dehydrogenases (41)	Short chain dehydrogenases (86)	Short chain dehydrogenases (18)
**2**	Acyl-CoA ligase/synthetase (40)	Acyl-CoA dehydrogenases (71)	Acyl-CoA ligase/synthetase (20)	Acyl-CoA dehydrogenases (40)	Acyl-CoA dehydrogenases (15)
**3**	Zinc-alcohol dehydrogenases (25)	Acyl-CoA ligase/synthetase (57)	Acyl-CoA dehydrogenases (18)	Acyl-CoA ligase/synthetase (33)	Enoyl-CoA hydrat./isom.(12)
**4**	Acyl-CoA dehydrogenases (24)	Aldehyde dehydrogenases (56)	Enoyl-CoA hydrat./isom.(17)	Dehydrogenases (28)	Aldehyde dehydrogenases (7)
**5**	Enoyl-CoA hydrat./isom.(22)	Zinc-alcohol dehydrogenases (52)	P450 monooxygenases (10)	Enoyl-CoA hydrat./isom. (23)	Acyl-CoA ligase/synthetase (6)
**6**	Aldehyde dehydrogenases (18)	Enoyl-CoA hydrat./isom.(44)	Succinate dehydrogenases (9)	Aldehyde dehydrogenases (22)	Acetyl-CoA C-acyltransferase (6)
**7**	Acetyl-CoA C-acyltransferase (13)	Acetyl-CoA C-acyltransferase (24)	Lipases (8)	P450 monooxygenases (20)	Acyl-CoA carboxyltransferase (5)
**8**	Monooxygenases (12)	Formyl-CoA transferases (18)	Secreted cutinases (5)	Monooxygenases (12)	Aminotransferases (5)
**9**	Amidases (11)	Monooxygenases (16)	Acetyl/Propionyl-CoA carboxylase (5)	Acyl-CoA thiolases (11)	Succinate dehydrogenases (3)
**10**	Secreted lipases (9)	P450 monooxygenases (14)	Acyl-CoA thiolases (5)	Oxidoreductases (9)	Cystathionine gamma-synthases (3)
	No. paralogous families = 145	No. paralogous families = 330	No. paralogous families = 103	No. paralogous families = 173	No. paralogous families = 36
