# Supplementary material for: The Genome of a Pathogenic Rhodococcus: Cooptive Virulence Underpinned by Key Gene Acquisitions
Source: PLoS Genet. 2010 Sep 30;6(9):e1001145. doi: 10.1371/journal.pgen.1001145 (PMC2947987; doi:10.1371/journal.pgen.1001145)
Supplement: Table S6 — Putative DosR/DevR boxes and corresponding transcriptional units in R. equi 103S a. Identified with CLC Main Workbench (http://www.clcbio.com/) and the 20-bp consensus DosR/DevR box 5′-NNNGGGHCNWWNGNCCCBNN-3′ (N = any nucleotide, H = A/C/T, B = C/G/T, W = A/T) defined by Park et al. [70] and modified according to [107], [108]. Accuracy cutoff ≥85%, intergenic position relative to start codon ≤150 nt. The conserved DosR motif is boxed, the invariant G6 and C8 positions and matching nucleotides at the opposite half-site of the palindrome are shaded in black, deviations from the consensus motif are shown in lower case. (0.12 MB PDF) [file pgen.1001145.s021.pdf]

Table S6

Putative DosR box	Position start codon	Strand	CDS	Product
GAGGGGCCGATTGTCaCCGG	-21	+	REQ01890	Putative haloacid dehalogenase-like hydrolase
TTCGGGACCAAAGTCCCGTC	-94	−	REQ10890	Putative nitric oxide dioxygenase
			REQ10900	Putative transcriptional regulator
GACGGGACTTTGGTCCCGAA	-140	+	REQ10910	Putative universal stress family protein
			REQ10920	Oxidoreductase
			REQ10930	Putative universal stress family protein
GAAaGGACGgTGGACCCCAG	-55	+	REQ12550	TetR family transcriptional regulator
			REQ12560	Transcription repair coupling factor Mfd
			REQ12570	Putative NTP pyrophosphohydrolase
AATGGGGCTTTGGTCCCGAT	-35	+	REQ12620	Putative transglycosylase
CTCGGGACAgTAGGCCaCGG	-140	−	REQ13620	Putative lipase
			REQ13630	Putative short chain dehydrogenase
GAAGGGCCCTTCGGCCCTGT	-80	+	REQ15220	6-phosphofructokinase PfkB
			REQ15230	Putative cation transporter ATPase P-type
CCGaGGACCTTGGGCCCTGT	-68	+	REQ15250	Putative pyruvate water dikinase
TTGGtGACCAACGACCCTGC	-77	−	REQ15260	Acr/HspX heat shock protein
GCAGGGTCGTTGGTCaCCAA	-129	+	REQ15270	Acyl-CoA dehydrogenase
			REQ15280	Acyl-CoA dehydrogenase
			REQ15290	Putative transcriptional regulator
TTGcGGACGcgTGTCCCCGG	-84	−	REQ21040	Putative TetR family transcriptional regulator
AACGGGACCTTCGTCCgTCG	-127	+	REQ27330	Putative HNH endonuclease
GGAGGGCCATTGGTCCCGAC	-122	−	REQ28280	Cytochrome c oxidase subunit IV
			REQ28290	Cytochrome c oxidase subunit II
TCGGtGACATTCGTCaCTCG	-24	+	REQ32410	Putative secreted lipase
GTCGGGACCTTAGGCCCTCG	-35	−	REQ39170	Cytochrome c oxidase subunit I
CTCGGGACTTTGGTCCCTAG	-76	−	REQ43290	Putative IclR family transcriptional regulator
CAAGGGACCTTCGACCCTAC	-114	+	REQ44290	Putative IclR family transcriptional regulator

[a] Although the functions of virulence regulator orthologs are not necessarily conserved in different pathogens [109], as *R. equi* is associated with chronic granulomatous infections, we investigated the DosR (REQ11020) two-component response regulator in some detail. In Mtb, DosR coordinates a response to hypoxia and NO thought to be important for survival within granulomes [70]. Only about 20 of the ≈50 genes of the Mtb DosR regulon had orthologs in *R. equi*, of which only three, REQ15260, REQ15220 and REQ10910, were preceded by a putative DosR binding site. Interestingly, these are homologs of DosR-dependent Mtb genes highly upregulated during hypoxia/NO-induced non-replicative state, respectively: *rv2031c*/*acr* (α-crystallin stress chaperone and immunodominant Mtb antigen), *rv2029c*/*pfkB* (phosphofructokinase B), and Rv2623 (Usp required for establishing chronic infection) [110]. Other putative DosR box-associated *R. equi* gene products include an additional Usp (a total of six are DosR-regulated in Mtb) and NO dioxygenase REQ10890, an enzyme that detoxifies NO generating nitrate (which may be recycled via REQ4200-30/*nar*GHIJ; see text). Thus, while the Mtb DosR regulon is not conserved, the homologous regulator in *R. equi* appears to control reminiscent functions potentially relevant to infection.
