# Supplementary material for: The Genome of a Pathogenic Rhodococcus: Cooptive Virulence Underpinned by Key Gene Acquisitions
Source: PLoS Genet. 2010 Sep 30;6(9):e1001145. doi: 10.1371/journal.pgen.1001145 (PMC2947987; doi:10.1371/journal.pgen.1001145)
Supplement: Table S7 — Minimal inhibitory concentrations (MIC) of R. equi 103S to various antibiotics. Determined by the broth microdilution method. The data are consistent with previously reported antimicrobial susceptibility studies of R. equi isolates [111]–[116]. (0.06 MB PDF) [file pgen.1001145.s022.pdf]

Table S7

Antibiotic	MIC (μg/ml)	Susceptibility [a]
Ampicillin	4	R
Cefixime	>1	R
Cefepime	>2	R
Cefotaxime	4	R
Cefuroxime	4	R
Ciprofloxacin	4	R
Clindamycin	>1	R
Claritromycin	<0.25	S
Cotrimoxazole	>2	R
Erythromycin	0.5	S
Levofloxacin	1	R
Meropenem	0.5	S
Penicillin G	4	R
Rifampicin	<0.5	S
Tetracyclin	>4	R
Teicoplanin	<0.25	S
Vancomycin	<0.25	S

[a] R = resistant, S = sensitive
