# Supplementary material for: The Genome of a Pathogenic Rhodococcus: Cooptive Virulence Underpinned by Key Gene Acquisitions
Source: PLoS Genet. 2010 Sep 30;6(9):e1001145. doi: 10.1371/journal.pgen.1001145 (PMC2947987; doi:10.1371/journal.pgen.1001145)
Supplement: Table S10 — Virulence plasmid-chromosome crosstalk. Gobal microarray expression analysis of R. equi 103S and an isogenic plasmid-cured derivative (103SP−) during exponential growth in LB medium (OD600 = 0.8) in the indicated conditions (part A of table, 30°C-pH 8.0 = vap PAI gene-downregulating conditions; part B of table, 37°C-pH 6.5 = vap PAI gene-activating conditions [72], [73]). Chromosomal genes differentially expressed with P≤0.05 and fold-change cutoff ≥2 are listed. Expression data are presented as average fold-change of 103S relative to 103SP−; positive values indicate upregulation in the presence of the plasmid. (0.12 MB PDF) [file pgen.1001145.s025.pdf]

Table S10

Locus tag	Fold change	*P* value	Product
**(A) Genes differentially regulated at 30ºC pH 8.0 (*n* = 7, of which 5 upregulated by plasmid)** (*n* = 36, of which 19 upregulated by plasmid, with ≥ 1.5 cutoff)			
REQ02840	-2.84	0.0256	Putative glycerophosphodiester phosphodiesterase
REQ43300	2.14	0.0284	Putative alpha/beta hydrolase
REQ43350	2.16	0.0350	Putative ABC transporter ATPase subunit
REQ43360	2.26	0.0443	Putative ABC transporter substrate binding lipoprotein
REQ43450	2.03	0.0127	Putative alpha/beta hydrolase
REQ44840	2.42	0.0307	Conserved hypothetical protein
REQ45850	-2.15	0.0128	Putative secreted alkaline phosphatase
**(B) Genes differentially regulated at 37ºC pH 6.5 (*n* = 88, of which 60 upregulated by plasmid)** (*n* = 279, of which 168 upregulated by plasmid, with ≥ 1.5 cutoff)			
REQ00440	3.33	0.0106	Glutamate synthase large subunit GltA1
REQ01060	2.11	0.0069	Putative carboxylesterase
REQ01100	-3.48	0.0157	Putative methylmalonate-semialdehyde dehydrogenase
REQ01110	-2.05	0.0443	Putative MFS transporter
REQ01180	-2.16	0.0072	Putative copper transporter
REQ02960	2.71	0.0016	Secreted prephenate dehydrogenase
REQ03030	2.36	0.0041	Conserved hypothetical protein
REQ03860	-2.10	0.0297	Putative high affinity substrate binding lipoprotein
REQ04070	3.99	0.0012	Putative integral membrane protein YrbE1A
REQ05600	2.15	0.0258	Putative aminotransferase
REQ05610	2.03	0.0019	Hypothetical protein
REQ05780	2.35	0.0025	Putative D-alanyl-D-alanine carboxypeptidase
REQ06440	4.46	0.0016	Putative ABC transporter integral membrane subunit
REQ06630	2.88	0.0038	Putative glyoxalase family protein
REQ07260	2.16	0.0184	Oxidoreductase
REQ07300	2.27	0.0282	Putative FMN-dependent monooxygenase
REQ07860	-2.09	0.0031	Phosphoribosylamine-glycine ligase PurD
REQ08550	-2.14	0.0428	Conserved hypothetical protein
REQ08560	-2.24	0.0414	Thiosulfate sulfurtransferase
REQ08750	4.30	0.0407	Putative MFS transporter
REQ09520	-2.74	0.0013	Putative transglycosylase
REQ10050	-2.27	0.0470	Oxidoreductase
REQ11370	2.39	0.0091	Conserved hypothetical protein
REQ11870	3.31	0.0050	MerR family transcriptional regulator
REQ13530	2.87	0.0176	Zinc binding alcohol dehydrogenase
REQ13620	2.18	0.0010	Putative lipase
REQ13740	2.83	0.0091	Putative 4-carboxymuconolactone decarboxylase
REQ13990	3.52	0.0201	Hypothetical protein
REQ14020	2.83	0.0048	Putative MarR family transcriptional regulator
REQ15030	2.29	0.0256	Putative phosphoribosyltransferase
REQ16680	2.02	0.0025	Putative integral membrane protein
REQ17020	2.45	0.0004	Hypothetical protein
REQ18110	2.29	0.0019	Magnesium chelatase
REQ18690	2.31	0.0327	Putative integral membrane protein
REQ18840	-2.57	0.0094	Putative short chain dehydrogenase
REQ18850	-2.05	0.0107	Putative acyl-CoA ligase/synthetase
REQ18860	-2.23	0.0245	Putative aldehyde dehydrogenase

(B) Genes differentially regulated at 37ºC pH 6.5 (cont.)

REQ18880	-2.07	0.0335	Putative cytochrome P450 monooxygenase
REQ22390	2.15	0.0232	Conserved hypothetical protein
REQ23850	5.33	0.0066	Putative anthranilate synthase
REQ23860	5.30	0.0069	Putative chorismate mutase
REQ25270	-2.02	0.0117	Acetyl-CoA C-acyltransferase
REQ26050	2.18	0.0040	Putative anti sigma factor
REQ27260	-2.70	0.0074	8-amino-7-oxononanoate synthase BioF
REQ28180	-2.04	0.0326	Putative secreted peptidase
REQ28740	2.26	0.0454	Putative integral membrane protein
REQ29370	-2.54	0.0220	Ferredoxin-nitrite/sulphite reductase CysI
REQ29380	-2.56	0.0288	Phosphoadenylyl-sulfate reductase (thioredoxin) CysH
REQ29390	-2.31	0.0271	Sulfate adenylyltransferase subunit 2 CysD
REQ30360	2.26	0.0124	Putative secreted lipase
REQ30520	2.13	0.0304	Hypothetical protein
REQ30830	2.64	0.0062	Hypothetical protein
REQ31260	2.22	0.0030	Putative AsnC family transcriptional regulator
REQ31910	3.01	0.0037	Putative NUDIX superfamily hydrolase
REQ31980	2.33	0.0242	Putative siderophore binding protein
REQ32820	3.08	0.0081	Putative secreted protein
REQ32850	-2.11	0.0465	Putative integral membrane protein
REQ33100	-2.40	0.0217	Putative diacylglycerol kinase family protein
REQ34390	5.34	0.0020	Putative secreted ribonuclease
REQ36110	-2.92	0.0476	Putative MFS transporter
REQ36820	-2.45	0.0496	50S ribosomal protein L7/L12 RplL
REQ38080	4.09	0.0003	Conserved hypothetical protein
REQ38550	2.25	0.0290	Putative DeoR family transcriptional regulator
REQ38720	2.94	0.0042	Putative MFS transporter
REQ39080	-2.05	0.0123	Putative integral membrane protein
REQ39440	-2.42	0.0294	Putative membrane protein
REQ39520	3.02	0.0499	Hypothetical protein
REQ40020	-2.48	0.0197	Branched-chain amino acid ABC transporter substrate binding lipoprotein
REQ40030	-2.04	0.0114	Putative polyketide cyclase
REQ41040	2.16	0.0411	Putative MerR family transcriptional regulator
REQ41570	2.14	0.0072	Conserved hypothetical protein
REQ41590	3.36	0.0006	Putative ABC transporter ATPase subunit
REQ41600	2.45	0.0158	Putative ABC transporter integral membrane subunit
REQ41610	2.38	0.0313	Cobalamin biosynthesis protein CbiM
REQ41630	2.02	0.0079	Putative ABC transporter integral membrane subunit
REQ41750	2.28	0.0203	Putative secreted protein
REQ41800	2.04	0.0072	Hydrogenase large subunit
REQ41810	2.03	0.0146	Hydrogenase small subunit
REQ41840	2.22	0.0140	Putative TetR family transcriptional regulator
REQ41980	2.24	0.0333	Histidine ammonia-lyase HutH
REQ42100	3.90	0.0378	Putative AsnC family transcriptional regulator
REQ42210	2.90	0.0032	Putative LuxR family transcriptional regulator
REQ43440	2.02	0.0193	Hypothetical protein
REQ44840	4.80	0.0024	Conserved hypothetical protein
REQ45250	-2.02	0.0041	Putative iron binding alcohol dehydrogenase
REQ45370	2.12	0.0331	Urease accessory protein UreG
REQ45460	2.95	0.0062	Putative oligopeptide/dipeptide ABC transporter ATPase subunit
REQ46630	2.03	0.0086	Putative ABC transporter ATPase subunit
