# Supplementary material for: The Genome of a Pathogenic Rhodococcus: Cooptive Virulence Underpinned by Key Gene Acquisitions
Source: PLoS Genet. 2010 Sep 30;6(9):e1001145. doi: 10.1371/journal.pgen.1001145 (PMC2947987; doi:10.1371/journal.pgen.1001145)
Supplement: Table S11 — Plasmid gene-containing coregulated clusters. Gene allocation defined by graph clustering of the transcription network shown in Figure 5A. (A) Plasmid backbone cluster. Shown for each gene, average pairwise comparison ratios of normalized microarray expression data from exponential cultures of R. equi 103S in LB medium (OD600 = 0.8) at 37°C relative to 20°C (pH 6.5). This cluster contains only plasmid genes, virtually all from the housekeeping backbone and mostly constitutively expressed in the experimental conditions tested (see Figure S12B). (B) Same information as in (A) but for the plasmid vap PAI-coexpressed cluster a, in the indicated conditions. P versus NP, pairwise comparison of R. equi 103S and its isogenic plasmidless derivative 103SP− in vap gene-activating conditions [72], [73]. In bold, fold change differences ≥1.5 and P≤0.05. (C) Short list of vap PAI-coexpressed chromosomal genes and putative functions. Genes from part B not showing significant differential regulation by both temperature (at least one experimental condition) and plasmid in pairwise comparisons have been excluded (fold-change ≥1.5, P≤0.05 two-tailed Student's t test). (0.16 MB PDF) [file pgen.1001145.s026.pdf]

Table S11A

Locus tag	Location	Fold change 37ºC vs 20ºC (pH 6.5)	*P* value	Product
pVAPA_0020	Plasmid backbone	3.14	0.0576	Hypothetical protein
pVAPA_0030	Plasmid backbone	2.92	0.0614	Putative methylase/helicase
pVAPA_0040	Plasmid backbone	1.32	0.4121	Putative cell filamentation protein Fic-like
pVAPA_0050	Plasmid backbone	1.31	0.2229	Hypothetical protein
pVAPA_0060	Plasmid backbone	1.24d	0.5092	Hypothetical protein
pVAPA_0070	Plasmid backbone	-1.02	0.9453	Hypothetical protein
pVAPA_0080	Plasmid backbone	1.66	0.0854	TrwC-type relaxase / conjugal transfer protein TraA
pVAPA_0090	Plasmid backbone	1.27	0.5434	Hypothetical protein
pVAPA_0100	Plasmid backbone	2.16	0.0026	Putative type I DNA topoisomerase TopA
pVAPA_0110	Plasmid backbone	1.37d	0.2120	Hypothetical protein
pVAPA_0120	Plasmid backbone	1.38	0.1535	Putative conjugative transfer protein TraG/TraD
pVAPA_0130	Plasmid backbone	1.33	0.2020	Putative membrane protein
pVAPA_0160	Plasmid backbone	1.31	0.0370	VirB4-like conjugal transfer protein
pVAPA_0180	Plasmid backbone	-1.34	0.3330	TrbL (VirB6)-like conjugal transfer protein
pVAPA_0190	Plasmid backbone	1.64	0.3056	Putative membrane protein
pVAPA_0280	Plasmid backbone	-1.27	0.0539	Putative helix turn helix protein
pVAPA_0290	Plasmid backbone	1.23d	0.5654	Putative partitioning ATPase ParA
pVAPA_0310	Plasmid backbone	2.10	0.0391	Putative bacteriophage excisionase
pVAPA_0320	Plasmid backbone	-2.11	0.0208	Hypothetical protein
pVAPA_0340	Plasmid backbone	1.25	0.2695	Hypothetical protein
pVAPA_0350	Plasmid backbone	-2.07	0.0000	Putative helix-turn-helix protein
pVAPA_0361	Plasmid backbone	-1.06	0.6669	Putative integral membrane protein
pVAPA_0370	Plasmid backbone	1.21	0.4969	Putative membrane protein
pVAPA_0380	Plasmid backbone	1.10	0.6188	Hypothetical protein
pVAPA_0390	Plasmid backbone	-1.22	0.1059	Putative DNA helicase DnaB
pVAPA_0400	Plasmid backbone	1.40	0.8189	Transposon resolvase-like protein ResA
pVAPA_0420	Plasmid backbone	-1.20	0.5533	RepA/CopG-like regulator of plasmid copy number
pVAPA_0440	*vap* PAI	1.95	0.1765	Lsr2-like nucleoid-associated protein
pVAPA_0480	*vap* PAI	3.48	0.0083	LysR family transcriptional regulator (VirR)
pVAPA_0560	*vap* PAI	11.78	0.0474	Hypothetical protein
pVAPA_0740	*vap* PAI	1.65	0.0403	Hypothetical protein
pVAPA_0800	*vap* PAI	2.46	0.1308	Putative secreted chorismate mutase
pVAPA_0810	Plasmid backbone	1.12	0.1369	InvA-like invertase/resolvase
pVAPA_0830	Plasmid backbone	2.29	0.4529	Putative integral membrane protein
pVAPA_0840	Plasmid backbone	1.12d	0.0419	Putative integral membrane protein
pVAPA_0850	Plasmid backbone	2.06	0.6642	WhiB family transcriptional regulator

[a] Biolayout Express 3D [74,75] network and clustering settings: Pearson correlation threshold, 0.85; Markov clustering (MCL) algorithm inflation, 2.2; smallest cluster allowed, 3; edges/node filter, 10; rest of settings, default. Corresponding Biolayout Express layout file viewable in Supplementary Dataset file S1.

Table S11B

Locus tag	Location	Fold change 37ºC pH 6.5 vs 30ºC pH 8.0	*P* value	Fold change 37ºC vs 30ºC (pH 6.5)	*P* value	Fold change 37ºC vs 20ºC (pH 6.5)	*P* value	Fold change P vs NP (37ºC pH 6.5)	*P* value	Product
pVAPA_0170	Plasmid backbone	1.98	0.0781	1.63	0.0683	**2.44**	0.0184	–	–	Putative integral membrane protein
pVAPA_vapG	*vap* PAI	**19.70**	0.0195	**23.95**	0.0160	**42.72**	0.0143	–	–	Virulence associated protein VapG
pVAPA_0490	*vap* PAI	10.63	0.0751	11.36	0.0682	**15.80**	0.0488	–	–	Putative MFS transporter
pVAPA_vapH	*vap* PAI	12.79	0.0855	**30.48**	0.0394	**39.22**	0.0359	–	–	Virulence associated protein VapH
pVAPA_0520	*vap* PAI	10.81	0.0795	**21.87**	0.0401	**39.99**	0.0353	–	–	Hypothetical protein
pVAPA_0530	*vap* PAI	11.84	0.0916	**31.24**	0.0375	**50.96**	0.0265	–	–	Two-component system response regulator (Orf8)
pVAPA_vapX	*vap* PAI	5.80	0.1737	17.73	0.0543	**23.37**	0.0409	–	–	Virulence associated protein VapX
pVAPA_0570	*vap* PAI	**14.78**	0.0376	10.32	0.0541	**16.75**	0.0296	–	–	Hypothetical protein
pVAPA_0580	*vap* PAI	4.50	0.1138	5.69	0.0573	**10.03**	0.0233	–	–	Hypothetical protein
pVAPA_vapA	*vap* PAI	**30.28**	0.0469	**42.56**	0.0370	**66.79**	0.0306	–	–	Virulence associated protein VapA
pVAPA_vapI	*vap* PAI	27.50	0.0797	45.48	0.0591	**75.47**	0.0499	–	–	Virulence associated protein VapI
pVAPA_vapC	*vap* PAI	**35.78**	0.0338	**53.31**	0.0255	**92.82**	0.0217	–	–	Virulence associated protein VapC
pVAPA_vapD	*vap* PAI	**19.93**	0.0269	**24.79**	0.0216	**55.73**	0.0132	–	–	Virulence associated protein VapD
pVAPA_0710	*vap* PAI	6.85	0.0596	**7.59**	0.0461	**12.86**	0.0247	–	–	Hypothetical protein
pVAPA_vapE	*vap* PAI	**31.79**	0.0193	**40.35**	0.0157	**76.96**	0.0111	–	–	Virulence associated protein VapE
pVAPA_vapF	*vap* PAI	**13.70**	0.0432	**24.54**	0.0185	**66.33**	0.0071	–	–	Virulence associated protein VapF
REQ01880	Chromosome	1.31	0.1992	1.12	0.4373	1.28	0.1233	1.24	0.1197	Putative acyltransferase
REQ07190	Chromosome	1.43	0.2400	1.41	0.2422	1.45	0.2258	1.48	0.1974	Putative short chain dehydrogenase
REQ08750	Chromosome	2.58	0.1926	2.51	0.1576	**3.69**	0.0500	**4.29**	0.0406	Putative MFS transporter
REQ17220	Chromosome	**1.56**	0.0442	1.52	0.0712	**1.82**	0.0107	**1.63**	0.0242	MarR family transcriptional regulator
REQ17820	Chromosome	1.40	0.0580	1.31	0.0498	1.43	0.0114	1.34	0.0293	TetR family transcriptional regulator
REQ18520	Chromosome	1.80	0.1393	1.74	0.1926	2.31	0.0610	2.02	0.0904	Haloacid dehalogenase-like hydrolase
REQ20700	Chromosome	**1.62**	0.0155	1.41	0.0086	1.39	0.0066	**1.62**	0.0009	GNAT acetyltransferase
REQ23540	Chromosome	1.51	0.2227	1.76	0.1192	**2.31**	0.0394	2.00	0.0645	Hypothetical protein
REQ23850	Chromosome	**3.39**	0.0237	**4.20**	0.0120	**3.73**	0.0177	**5.33**	0.0065	Anthranilate synthase
REQ23860	Chromosome	**3.81**	0.0188	**3.71**	0.0169	**3.24**	0.0240	**5.30**	0.0068	Chorismate mutase
REQ28410	Chromosome	1.43	0.2892	1.63	0.1537	1.85	0.0924	1.62	0.1561	Conserved hypothetical protein
REQ29670	Chromosome	1.91	0.1471	1.56	0.1098	**2.53**	0.0126	**1.95**	0.0368	Nicotinate-nucleotide adenylyltransferase
REQ32960	Chromosome	2.83	0.0622	1.73	0.1412	**3.48**	0.01946	**2.32**	0.0500	GntR family transcriptional regulator
REQ37460	Chromosome	1.21	0.1823	1.20	0.1025	**1.50**	0.00353	**1.50**	0.0050	Glutamate-1-semialdehyde 2,1-aminomutase HemL
REQ37540	Chromosome	**1.52**	0.0235	**1.50**	0.0338	1.14	0.01093	**1.51**	0.0168	Putative metal ion transporter related protein
REQ41600	Chromosome	1.92	0.1007	1.52	0.2271	**2.97**	0.00701	**2.45**	0.0158	Putative ABC transporter integral membrane subunit
REQ42210	Chromosome	**2.53**	0.0078	**1.70**	0.0436	**1.92**	0.01998	**2.90**	0.0032	LuxR family transcriptional regulator
REQ46120	Chromosome	1.60	0.1553	1.48	0.2387	1.91	0.07371	1.72	0.0970	Putative MFS transporter

[a] Biolayout Express 3D [73,74] network and clustering settings: Pearson correlation threshold, 0.85; Markov clustering (MCL) algorithm inflation, 2.2; smallest cluster allowed, 3; edges/node filter, 10; rest of settings, default. Corresponding Biolayout Express layout file viewable in Supplementary Dataset file S1.

Table S11C

Locus tag	Product	Putative function / comments
REQ08750	Putative MFS transpoter	Unknown [a]
REQ17220	MarR family transcriptional regulator	Unknown
REQ20700	GNAT acetyltransferase	Unknown [b]
REQ23850	Anthranilate synthase	Shikimate pathway; biosynthesis of tryptophan, metabolism of glutamine/glutamate
REQ23860	Chorismate mutase	Shikimate pathway; biosynthesis of tyrosine and phenylalanine
REQ29670	NadD-like nicotinate-nucleotide adenylyltransferase	NAD(P) biosynthesis; cell redox balance and general metabolism
REQ32960	GntR family transcriptional regulator	Unknown [c]
REQ37460	Glutamate-1-semialdehyde 2,1-aminomutase HemL	Porphyrin metabolism; biosynthesis of tetrapyrroles and heme group
REQ37540	Putative metal ion transporter protein	Part of a P-type ATPase driven metal ion transporter (REQ37530-600) [d]
REQ41600	Putative ABC transporter integral membrane subunit	Part of CbiMNQO-like cobalt ABC transporter (REQ41590-640) [e]
REQ42210	Forkhead (phosphopeptide recognition) domain-containing LuxR transcriptional regulator	Unknown

[a] Cotranscribed with genes encoding hypothetical proteins in HGT island.

[b] Cotranscribed with upstream REQ20710 encoding MFS transporter, part of HGT island.

[c] Possibly regulating divergently transcribed operon REQ32950-32900 (putative MFS transporter, nitrate/nitrite transporter NarK, NirBD nitrite reductase).

[d] Cotranscribed in an operon that includes a pyrroloquinoline-quinone (PQQ)-dependent dehydrogenase and *hemACDB* (REQ37610-640) and *hemK* (REQ37480) homologs. Possibly involved in tetrapyrrole/heme biosynthesis.

[e] Part of the cobalamin (vitamin B12) biosynthesis pathway.
