# Supplementary material for: The Genome of a Pathogenic Rhodococcus: Cooptive Virulence Underpinned by Key Gene Acquisitions
Source: PLoS Genet. 2010 Sep 30;6(9):e1001145. doi: 10.1371/journal.pgen.1001145 (PMC2947987; doi:10.1371/journal.pgen.1001145)
Supplement: Table S12 — Software and databases used to annotate and analyze the R. equi 103S genome. (0.06 MB PDF) [file pgen.1001145.s027.pdf]

Table S12

Software	Analysis	Reference
Alien Hunter	Detection of local compositional biases using variable-order motif distributions method to identify horizontally acquired (HGT) DNA regions	[92]
Artemis	Genome viewer and annotation tool	[117]
Artemis Comparison Tool (ACT)	Genome sequence multiple alignment and comparison viewer	[118]
BLASTCLUST	Clustering of proteomes	[119]
BioLayout Express^{3D}	Network analysis of microarray expression data	[74,75]
BLAST	Homology prediction	[119]
GLIMMER, RBS finder & TransTermHP	Automated detection of potential coding sequences (CDSs), Shine-Dalgarno sequences and rho-independent transcription terminators	[120]
Pathway Tools	Metabolic pathway reconstruction	[121]
PFAM database	Domain-based identification of protein families	[122]
PROSITE	Prediction of lipoproteins and proteins recognized by sortases	[123]
Reciprocal best-match BLASTP/FASTA	Determination of orthologous gene sets	[119]
REPuter	Sequence repeats	[124]
RFAM database	Stable non-coding RNAs	[125]
SignalP	Secreted proteins	[126]
TMHMM	Membrane/transmembrane proteins	[127]
