# Supplementary material for: The Genome of a Pathogenic Rhodococcus: Cooptive Virulence Underpinned by Key Gene Acquisitions
Source: PLoS Genet. 2010 Sep 30;6(9):e1001145. doi: 10.1371/journal.pgen.1001145 (PMC2947987; doi:10.1371/journal.pgen.1001145)
Supplement: Table S13 — GenBank accession nos. of the genomes used in this study. R. erythropolis PR4 and R. opacus B4 genomes published online by NITE, the Japanese National Institute for Technology and Evaluation (http://www.nite.go.jp/index-e.html). (0.08 MB PDF) [file pgen.1001145.s028.pdf]

Table S13

**Species**	**Accession no.**
Arthrobacter sp. FB24	CP000454
Bifidobacterium longum NCC2705	AE014295
Clavibacter michiganensis subsp. *michiganensis* NCPPB 382	AM711867
Corynebacterium diphtheriae NCTC 13129	BX248353
Corynebacterium glutamicum ATCC 13032	BX927147
Frankia sp. CcI3	CP000249
Leifsonia xyli subsp. *xyli* str. CTCB07	AE016822
Mycobacterium smegmatis str. MC2 155	CP000480
Mycobacterium tuberculosis H37Rv	AL123456
Nocardia farcinica IFM 10152	AP006618
Propionibacterium acnes KPA171202	AE017283
***Rhodococcus equi* 103S**	FN563149
Rhodococcus erythropolis PR4	AP008957
Rhodococcus jostii RHA1	CP000431
Rhodococcus opacus B4	AP011115
Rubrobacter xylanophilus DSM 9941	CP000386
Saccharopolyspora erythraea NRRL2338	AM420293
Salinispora tropica CNB-440	CP000667
Streptomyces coelicolor A3(2)	AL645882
Thermobifida fusca YX	CP000088
Tropheryma whipplei TW08/27	BX072543
