# Supplementary material for: The Genome of a Pathogenic Rhodococcus: Cooptive Virulence Underpinned by Key Gene Acquisitions
Source: PLoS Genet. 2010 Sep 30;6(9):e1001145. doi: 10.1371/journal.pgen.1001145 (PMC2947987; doi:10.1371/journal.pgen.1001145)
Supplement: Table S14 — Oligonucleotide primers used for mutant construction and complementation. SpeI, XbaI and EcoRV restriction sites used for the cloning of PCR products are underlined. (0.05 MB PDF) [file pgen.1001145.s029.pdf]

Table S14

Primer	Sequence 5'→3'	Use
CMDEL1	TATATAACTAGTCTCAGCACCGATACTCCCGAGAACTGC	Construction of ΔREQ23860
CMDEL2	GACACCAGCGGCCCGACGAATCGCGCGACG	
CMDEL3	CGTCGCGCGATTCGTCGGGCCGCTGGTGTC	
CMDEL4	TATATATCTAGAGTGCCACTCGAACAGCGGCGC	
ASDEL1	TATATAACTAGTGCGTCCACGGCCTGCTGATGGCGG	Construction of ΔREQ23850
ASDEL2	CTCCTGATCGACAACCCGACCGGGGACGGG	
ASDEL3	CCCGTCCCCGGTCGGGTTGTCGATCAGGAG	
ASDEL4	TATATATCTAGAGCCGCCGCGAACGCCGTGGCC	
CACOMP1	TATATATCTAGACGCCACCGGGGCGGGTG	Complementation of ΔREQ23860 and ΔREQ23850
CACOMP2	TATATAGATATCTCATCGATCACCCGCCCCGTCC	
